# EP300 knockdown reduces cancer stem cell phenotype, tumor growth and metastasis in triple negative breast cancer

**DOI:** 10.1186/s12885-020-07573-y

**Published:** 2020-11-10

**Authors:** Alexander Ring, Pushpinder Kaur, Julie E. Lang

**Affiliations:** 1 Division of Surgical Oncology, Department of Surgery and University of Southern California Norris Cancer Center, University of Southern California, Los Angeles, CA USA; 2 Present Address: Department of Medical Oncology and Hematology, Universitätsspital Zürich, Rämistrasse 100, 8091 Zurich, Switzerland

**Keywords:** Triple negative breast cancer (TNBC), Basal-like BC, E1A-associated protein p300 (EP300), Cancer stem cells (CSC), ATP-binding cassette super-family G member 2 (ABCG2)/breast Cancer resistance protein (BCRP), Metastasis

## Abstract

**Background:**

Triple negative breast cancer (TNBC) is an aggressive breast cancer subtype with basal features, lacking the expression of receptors targeted successfully in other breast cancer subtypes. Treatment response to adjuvant and neoadjuvant chemotherapy is often short-lived and metastatic spread occurs at higher rates than other subtypes within the first five years after diagnosis. TNBCs exhibit stem cell features and are enriched for cancer stem cell (CSC) populations. E1A Binding Protein P300 (EP300) is a large protein with multiple cellular functions, including as an effector in stem cell biology.

**Methods:**

We used a genetic knockdown (KD) model of EP300 in TNBC cell lines to investigate the effect on CSC phenotype, tumor growth and metastasis. Side population assay and tumorsphere suspension culture were used in vitro*.* Xenograft mouse models were used for in vivo studies. We performed in silico analysis of publicly available gene expression data sets to investigate CSC gene expression and molecular pathways as well as survival outcomes associated with EP300 expression in patients with TNBC and basal-like BC.

**Results:**

EP300 KD abolished the CSC phenotype by reducing ABCG2 expression, side population cells and tumorsphere formation capacity in vitro as well as tumor formation in a xenograft mouse model in vivo. Metastatic capacity was markedly reduced in EP300 KD cells in vivo, with no detection of circulating tumor cells. TCGA data analysis demonstrated that genes positively correlated with EP300 expression in TNBC and basal-like BC were associated with CSC biology. Survival analysis demonstrated that EP300 expression predicts poor recurrence free survival in TNBC and basal BC.

**Conclusion:**

We report a novel oncogenic role for EP300 in driving CSC phenotype representing a potential target to address tumor initiation and metastatic spread in TNBC and basal-like BC. EP300 might serve as a prognostic marker and potential therapeutic target in TNBC.

**Supplementary Information:**

The online version contains supplementary material available at 10.1186/s12885-020-07573-y.

## Background

Breast cancer (BC) is a heterogeneous disease with distinct subtypes each with different pathological and molecular features, variable responsiveness to therapy and clinical outcomes. Approximately 10–20% of all breast cancers are triple negative (TN) and lack the expression of estrogen and progesterone receptors as well as the human epidermal growth factor receptor (HER) 2.

Early disease progression and metastasis often occurs within the first 5 years after diagnosis. The lack of targeted therapy options contributes to worse survival for TNBC, despite receipt of cytotoxic chemotherapy [1–3]. Non-targeted neoadjuvant and adjuvant cytotoxic chemotherapy remains as the pharmacological backbone in the clinical management of TNBC. More recently, the Food and Drug Administration (FDA) approved poly-ADP-ribose polymerase (PARP) inhibitors for a subset (10–15%) of TNBCs harboring germline mutations in BRCA1/2 [4] that work via synthetic lethality in this subgroup [5]. Immunotherapy using immune checkpoint inhibitors alone or in combination with chemotherapy and targeted therapies are approved [6] or under active investigation (e.g. TOPACIO/KEYNOTE-162, KEYNOTE-086, KEYNOTE-173). Nevertheless, targeted therapies for TNBC are an urgent unmet need in the breast cancer research community and for patients affected by the disease. Molecular studies led to a more detailed understanding of TNBC by defining distinct subtypes with clinical and therapeutic implications, showing that these cancers often exhibit basal-like features and are enriched for cancer stem cell (CSC) populations [7–9]. CSCs are thought to be responsible for tumor initiation, drug resistance and metastasis [10] and were first described in BC by Al-Hajj et al. [11]. CSCs can be identified by the expression of ATP-binding cassette transporters (e.g. MDR1 or ABCG2) as a side population in FACS assays [12].

EP300 (E1A-associated protein p300) is a large (~300KDa), multi-domain protein that functions as a histone/ lysine acetyl transferase and chromatin remodeler as well as acetyltransferase for non-histone targets [13]. As part of various transcriptional complexes EP300 has important and diverse biological functions in cellular proliferation, cell cycle regulation, apoptosis, DNA damage repair, cell fate determination and stem cell pluripotency [14]. EP300 has been implicated in cancer biology in general [15] and breast cancer in particular [16], and has been described as both a tumor suppressor [16, 17] and more recently as an oncogene promoting tumor growth, metastatic potential and CSC phenotype in BC [18–20]. In TNBC specific EP300 mutations have been described and EP300 was included in the triple negative breast cancer team project biomarker panel [21]. We hypothesized that EP300 plays an oncogenic role in TNBC biology by regulating CSC populations. We show that knockdown of EP300 abrogates the CSC phenotype of TNBC cells and reduces tumor growth and metastasis*.*

## Methods

### Cell culture

MDA-MB-231 and BT20 cell lines were obtained from the American Type Culture Collection (ATCC) (Manassas, VA) and cultured in Dulbecco’s Modified Eagle Medium (DMEM) (Thermo Fisher Scientific, Canoga Park, CA) + 10% fetal bovine serum (FBS) (Atlanta Biologicals, Flowery Branch, GA) + 1% Antibiotic-Antimycotic mix (Thermo Fisher Scientific). CAL51 was obtained from The Leibniz Institute DSMZ - German Collection of Microorganisms and Cell Cultures GmbH (Braunschweig, Germany) and cultured in DMEM + 20% FBS. Cell lines were authenticated at the University of Arizona Genetics Core (UAGC, Tucson, AZ) using short tandem repeat (STR) profiling. Cell lines were also routinely tested to exclude mycoplasma contamination (MycoAlert™ PLUS Mycoplasma Detection Kit, Lonza, Basel, Switzerland). No ethics approval was required for cell line use. Cell cultures were maintained and propagated in cell culture flasks (Corning Inc., Corning, NY) in a humidified incubator at 37 °C and 5% CO_2_. To mimic in vivo conditions more closely, cells were also cultured in low glucose (1000 mg/L) DMEM (Thermo Fisher Scientific) and under low oxygen conditions (2%). For tumorsphere suspension culture, 20,000 cells per well were cultured in MammoCult culture medium (STEMCELL Technologies, Vancouver, Canada) in ultra-low adherence 6-well culture plates (Corning Inc.) for up to 14 days. Spheres of ≥50 μm were counted.

### Cell migration assay

To measure cell migration, confluent cell monolayers of 1 × 106cells per well seeded overnight in 6-well plates were scratched using 200 μL plastic micropipette tips. Gap area was recorded immediately after scratching and 72 h later using an inverted microscope (AxioVert.A1, Zeiss, Oberkochen, Germany). Gap area was quantified using ImageJ software.

### Stable and transient transfection

Lipofectamine 2000 reagent (Thermo Fisher Scientific) was used for stable and transient gene knockdown (KD) according to the manufacturer’s protocol. Before transfection, cells were seeded at 3 × 10^5^ cells per well in 6-well plates or 1 × 10^5^ cells per well in 24-well plates and incubated with transfection medium for up to 48 h. For stable knockdown EP300 and scramble shRNA vectors (sense 5′- CCGGGCCTTCACAATTCCGAGACATCTCGAGATGTCTCGGAATTGTGAAGGCTTTTTG.

− 3′) were used (Sigma Aldrich, St. Louis, MO). For selection of cell clones with successful DNA integration, KD cells were further cultured as single cells in 96-well plates under the addition of G418 (geneticin) at 50 μg/ml. Knockdown efficiency was validated via western blotting as described below. siRNA transient gene KD for EP300 (siRNA ID 106443) and negative control were performed in duplicate using Silencer Select siRNAs (Thermo Fisher Scientific). As a control for transfection efficiency, the cells were simultaneously transfected with BLOCK-iT Alexa Fluor Red Fluorescent Oligo (Thermo Fisher Scientific) and red fluorescence expression was validated after 24 h transfection using a Zeiss Axiovert 200 inverted microscope. Gene knockdown was validated via qRT-PCR.

### Quantitative reverse transcription polymerase chain reaction (qRT-PCR)

Total RNA extraction was performed using TRIzol reagent (Thermo Fisher Scientific) according to the manufacturer’s protocol. First strand synthesis was performed using the qScript cDNA Supermix (Quantabio, Beverly, MA) and 1 μg of total RNA per reaction. The following reaction conditions were used on a T100 thermal cycler (Bio-Rad, Irvine, CA): 25 °C for 5 min, 42 °C for 30 min, 85 °C for 5 min. Samples were prepared as triplicates.

For gene expression quantification, SYBR Green RT-PCR master mix (Quantabio) or TaqMan assay (Thermo Fisher Scientific) was used. Reaction were performed in 96-well plates (Bio-Rad), using 2 μL cDNA, 12.5 μL SYBR Green master mix, 1 μL each forward and reverse primer probes and 8.5 μL nuclease free deionized water. The following reaction conditions were used on a MyiQ or CFX96 Real-Time system (Bio-Rad): 95 °C for 3 min, 40 cycles of 95 °C for 20 s, 60 °C for 20 s, 72 °C for 30 s. Primer sequences were obtained via the Harvard primer bank and synthesized by ValueGene (San Diego, CA). To ensure gene-specific priming, the primer sequences were validated using the NCBI BLAST tool and only exon-junction spanning primers used. Primer sequences and corresponding genes are listed in supplementary Table S2.

### Fluorescence activated cell sorting (FACS)

Cells were harvested using non-enzymatic cell dissociation buffer (Sigma Aldrich). FACS was performed on a BD LSRFortessa (BD Biosciences, San Jose, CA) equipped with a 405 nm excitation wavelength.

#### Side population assay

The cell number was adjusted to 1 × 10^6^ cells per mL and re-suspended in complete culture medium (DMEM + 10% FBS + 1% pen strep). Five μg of Hoechst 33342 dye (Sigma Aldrich) was added to each sample. For negative controls, samples were prepared in the same way, plus the addition of 100 μM verapamil (Sigma-Aldrich) to block dye efflux. The samples were incubated for 2 h at 37 °C in a cell culture incubator and gently agitated every 30 min. Following incubation, samples were immediately placed on ice and centrifuged at 1000 rpm at 4 °C for 5 min. Cell pellets were re-suspended in 300 μL cold PBS containing 5 μg/ml propidium iodide (PI) (Thermo Fisher Scientific) for dead cell exclusion. Our gating strategy included exclusion of cell debris (using forward area vs side scatter are), doublet exclusion (side scatter area vs. height), dead cell exclusion (using PI) and identification of SP cell using Hoechst red vs. Hoechst blue.

#### Antibody staining

Cells were re-suspended in FACS buffer (PBS + 2% FBS) at 1 × 10^6^ cells per 100 μL and incubated for 15 min on ice with 10 μL of allophycocyanin (APC)-conjugated mouse anti-human CD44 antibody (G44–26) (BD Biosciences) or APC-conjugated mouse anti-human CD24 antibody (32D12) (Miltenyi Biotech, Bergisch Gladbach, Germany). For dead cell exclusion,4′,6-diamidino-2-phenylindole (DAPI) (Sigma-Aldrich) nuclear dye solution was added.

#### Cell cycle analysis

Cells were washed twice in ice-cold FACS buffer (PBS + 2% FBS) and centrifuged at 1000 rpm for 5 min between washes. After the second wash, the cell pellets were re-suspended in ice cold 80% ethanol. The ethanol was added dropwise under constant vortex agitation, to a final volume of 500 μL ethanol per 1 × 10^6^ cells. Samples were stored at 4 °C until further use. For analysis, 10 μL of a 1 μg/ml 4′,6-diamidino-2-phenylindole (DAPI) (Sigma-Aldrich) nuclear dye solution was added to a 100 μL ethanol cell suspension.

### Protein quantification

Cells were washed twice with ice-cold PBS and subsequently scraped into ice-cold PBS plus protease inhibitor (Merck Millipore, Billerica, MA) and 1 μM Dithiothreitol (DTT) (Sigma-Aldrich), pelleted at 1000 rpm for 5 min and stored and − 80 °C until further use.

For protein extraction, the cell pellets were thawed on ice and processed using the Pierce M-PER Extraction Kit (Thermo Fisher Scientific) according to the manufacturer’s instructions. To ensure loading of equal amounts of protein for each sample, colorimetric protein quantification was performed using a Bio-Rad Protein Assay (Bio-Rad, Hercules, CA) and a SpectraMax M3 spectrophotometer (Molecular Devices, Sunnyvale, CA). For standard curve values, an albumin standard (Thermo Fisher Scientific) dilution serious was used. Protein extracts were mixed 1:1 with 2x Laemmli Sample Buffer (Bio-Rad) containing 5% of 2-mercaptoethanol (Sigma-Aldrich) and boiled for 5 min. The samples were loaded onto pre-cast 4–20% gradient gels (PAGEr Gold Precast gels, Lonza, Basel, Switzerland) including a Precision Plus Protein Dual Color Standard (Bio-Rad) for size determination. After gel electrophoresis (running buffer: 25 mM Tris HCl, 193 mM glycine, 0.1% sodium dodecyl sulfate (SDS), pH 8.3) the separated protein fractions were transferred onto nitrocellulose membranes (Bio-Rad) (transfer buffer: 80% Tris-Borate buffer [0.089 M Tris, 0.089 M Borate, pH 8.3], 20% methanol, 25 mM Tris HCl, 193 mM glycine) at 4 °C overnight. The membranes were then blocked in 5% skim milk for 60 min. Primary antibody incubation was performed in blocking solution over night at 4 °C. Primary antibodies: polyclonal rabbit anti-human p300 (N-15), monoclonal mouse anti-human ACTB (H-102) (both from Santa Cruz Biotechnologies). Secondary antibody incubation was performed for 60 min at room temperature. Secondary antibodies: goat anti-mouse IgG HRP conjugated; and goat anti-rabbit IgG HRP conjugated (both from Santa Cruz Biotechnologies). Subsequently the membranes were incubated with HRP substrate (GE Healthcare Life Sciences, Pittsburgh, PA) for 5 min. and chemiluminescence was recorded using a ChemiDoc MP gel imaging system (Bio-Rad). ImageJ software was used for quantification. Samples were prepared in technical duplicates.

### Animal experiments

Eight to ten weeks old female NOD scid gamma (NSG) mice (NOD scid IL2Rgamma^null^, The Jackson Laboratory, Bar Harbor, Maine) were used for all xenograft experiments. The animals were housed at the USC Zilkha Neurogenetic Institute vivarium, according to IACUC requirements (protocol number 11204). For tumor cell injection, the mice were anesthetized with 2.5–4% isoflurane (Santa Cruz Biotechnology, San Diego, CA) under continuous infusion via a nose cone. At the experimental endpoint (tumor size ~1500mm^3^ or mice showing significant signs of sickness due to cumulative tumor burden) mice were sacrificed using carbon dioxide in an enclosed chamber, followed by cervical dislocation. All animal procedures adhered to the ARRIVE guidelines [22].

#### Tumor cell line subcutaneous (s.c.) and tail vein injection

For subcutaneous xenograft experiments, cells were prepared at a concentration of 1 × 10^6^ cells per 100 μL in a 1:1 mix of 75 μL cell culture medium (DMEM/ F12) and 75 μL Matrigel basement membrane (BD Biosciences, San Jose, CA), for a final volume of 150 μL slurry. A 100 μL bolus of cell slurry was injected subcutaneously into the left hind flank region of each mouse and the puncture site sealed using degradable tissue adhesive (3 M Health Care, St. Paul, MN). Tumor growth was monitored weekly using a digital caliper (VWR, Radnor, PA). Tumor volume was calculated using the formula Volume = (Width × 2 × Length)/2. To investigate whether micro-environmental or paracrine factors affect tumor growth of MDA-MB-231^EP300KD^ cells, wild type cells expression mCherry and MDA-MB-231^EP300KD^ clone 1 (GFP positive) were injected as a 1:1 mix for a total of 1 × 10^6^ cells per mouse.

For tail vein injections, the mice were placed under a heating lamp for 2 min to dilate blood vessels. After subsequent immobilization in a rodent holder (Kent Scientific, Torrington, CT) 1 × 10^6^ cells in 100 μL culture medium were injected per mouse using hypodermic syringes.

#### In vivo imaging

Tumor growth and spread after tail vein injection was monitored intra-vitally once per week for a total of 5 weeks. Luciferin (Promega, Madison, WI) was prepared at a final concentration of 300 mg/ml. After induction of anesthesia as described above, 300 μL of luciferin was injected intraperitoneally per mouse and allowed to distribute through the body of the animal for 15 min before imaging. Imaging was performed using an IVIS Spectrum pre-clinical in vivo imaging system (PerkinElmer, Waltham, MA) under continued anesthesia. Images were taken after 10 s, 1 min and 3 min each for supine and prone position. Bioluminescence was quantified using the Living Image 4.2 software (PerkinElmer).

### Gene association and survival analysis

For gene association studies publicly available data sets from The Cancer Genome Atlas (TCGA) breast cancer cohort were used and analyzed via Ingenuity pathway (IPA) tool and cBioPortal [23–25]. The Kaplan-Meier (KM) plotter web interface was used to assesses the effect of EP300 gene expression on survival in breast cancer patients [26]. KM plotter is a meta-analysis-based biomarker assessment tool using a manually curated gene expression database downloaded from Gene Expression Omnibus (GEO), European Genome-phenome Archive (EGA) and The Cancer Genome Atlas (TCGA). The auto selected cut-off was used to define high vs. low expression, which computes the best threshold between the upper and lower quartiles based on sample selection. Recurrence free survival (RFS) was reported at 60-month survival.

### Statistical analysis

GraphPad PRISM (GraphPad Software Inc., La Jolla, CA) was used for statistical analysis of all experiments. Non-normal distribution was assumed, and appropriate non-parametric statistical tests were used; for experiments with single variables and groups of two Mann-Whitney test was used; for three or more groups, non-parametric one-way ANOVA (Kruskal-Wallis and Dunn’s multiple comparison) was used. For two variables and groups of two or more two-way ANOVA was used. Statistical hypothesis testing (Sidak test) was used to correct for multiple comparison. Data for repeats were presented as means and standard deviation. A two-tailed statistical significance level (alpha) of 0.05 for all statistical tests was considered meaningful to reject the null hypothesis. For survival analysis after tail vein injection, Kaplan-Meier curves were created and log-rank (Mantel-Cox) tests were used.

## Results

### Knockdown of EP300 in MDA-MB-231 cells leads to down-regulation of ABCG2 expression and elimination of the side population CSC phenotype

**Fig. 1:**
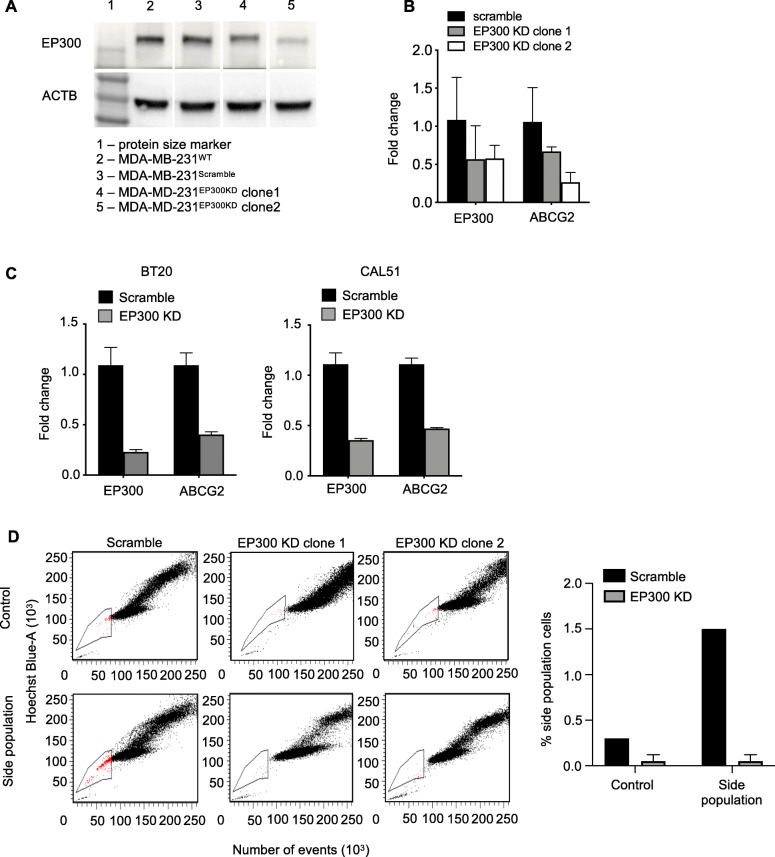
EP300 KD reduces ABCG2 levels and side population cells. **a,** Western plot protein quantification of EP300 and ACTB in MDA-MB-231^WT^, non-specific scramble shRNA and two stable shRNA EP300 KD clones (*n* = 1 each). **b,** EP300 and ABCG2 gene expression in MDA-MB-231 scramble and EP300 KD cells (*n* = 3 each). **c,** ABCG2 gene expression after transient siRNA KD of EP300 in three TNBC cell lines (BT20, CAL51) (*n* = 2 per cell line). **d,** FACS side population (SP) quantification in the MDA-MB-231^EP300KD^ (*n* = 2) and scramble (*n* = 1)

Reduction in protein and mRNA levels after EP300 knockdown in MDA-MB-231^EP300KD^ clones compared to wild type (WT) and a non-specific scramble control (MDA-MB-231^EP300KD^ clone 1: 27% KD, clone 2: 63% KD) are shown in Fig. 1a (for full membrane image see additional file 1) and Fig. 1b, respectively. Stable EP300 KD in MDA-MB-231 cells as well as transient KD in two other TNBC cell lines (BT20 and CAL51) reduced mRNA expression of ABCG2 (ATP Binding Cassette Subfamily G Member 2) (Fig. 1b and c). The Hoechst 33341 dye efflux assay (side population assay) demonstrated that both MDA-MB-231^EP300KD^ clones were depleted of cells with side population (SP) phenotype (scramble: 1.5%, MDA-MB-231^EP300KD^ clone 1: 0.1%, clone 2: 0%) (Fig. 1d). In summary, these results showed that EP300 KD led to a strong reduction in the expression of the CSC marker ABCG2 and abolished cells with CSC phenotype characterized by the side population in TNBC cells. no different in the distribution of CD44 and CD24 cell surface markers was observed between EP300 KD and scramble transfected MDA-MB-231 cells (supplementary Fig. 1).

### Tumorsphere formation capacity is reduced after EP300 KD in TNBC cells

**Fig. 2:**
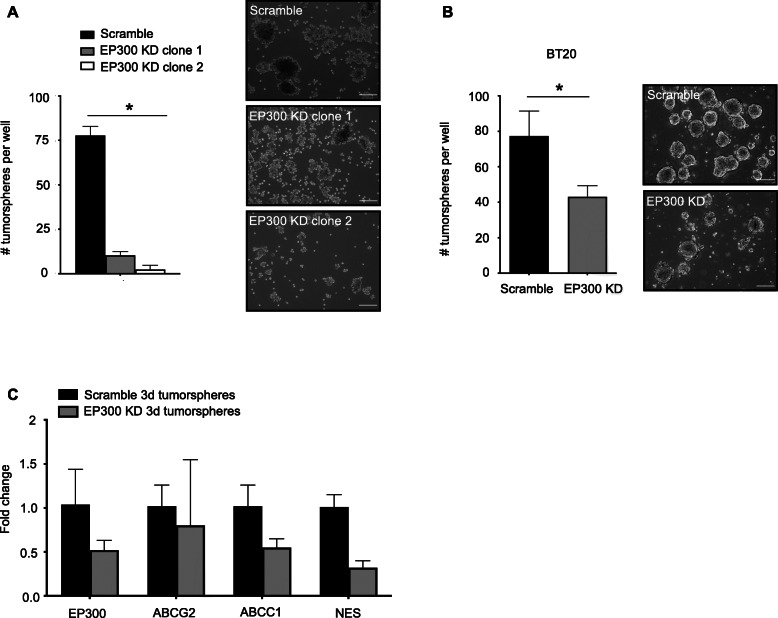
EP300 KD reduces tumorspheres formation. **a,** Tumorsphere assay (14 days in culture) of MDA-MB-231^EP300KD^ and scramble clones (*n* = 3 per condition). **b,** Tumorsphere assay (7 days in culture) after transient EP300 KD (48 h) in BT20 cells (*n* = 3 per cell line per condition) (scale bar 125 μm). (*p < 0.05, **p < 0.01). **C**, Gene expression analysis of CSC markers (EP300, ABCG2, ABCC1, NES) in scramble tumorspheres and EP300 KD (clone 2) tumorspheres (*n* = 3 per cell line)

MDA-MB-231^EP300KD^ clones showed significantly reduced tumorsphere formation capacity in vitro (scramble 78 ± 5, KD clone 1 10 ± 2 n.s., KD clone 2 2 ± 3 *p* = 0.02, *n* = 3 each) (Fig. 2a). Transient siRNA-mediated KD of EP300 in BT20 also showed reduction of tumorsphere formation, albeit to a lesser degree than stable KD (BT20 KD 43 ± 6 vs. scramble 77 ± 14, *p* = 0.018; *n* = 3 each) (Fig. 2b). Gene expression analysis showed reduced expression of CSC markers (EP300, ABCG2, ABCC1, NES) in EP300 KD tumorspheres compared to scramble tumorspheres (Fig. 2c). These results demonstrated that anchorage independent sphere formation as a characteristic of cell with CSC phenotype is reduced by EP300 knockdown to a varying degree in TNBC cell lines.

### Knockdown of EP300 abolishes tumor formation and causes G2/M blockade in vivo

**Fig. 3:**
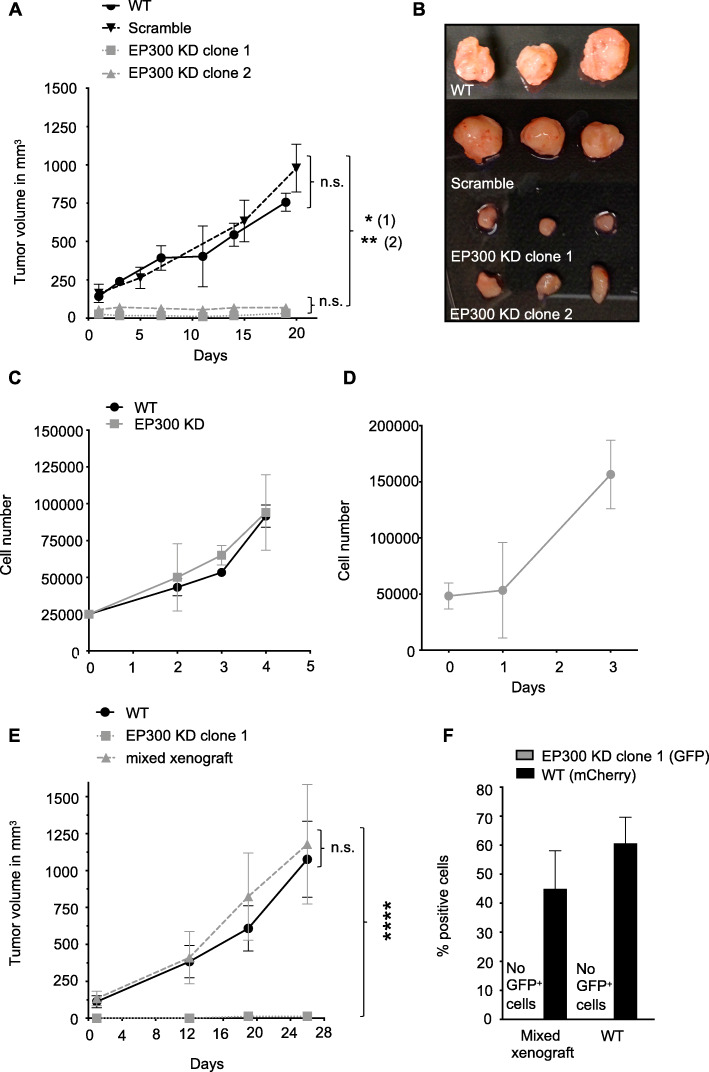
MDA-MB-231^EP300KD^ does not affect proliferation in vitro but abolishes tumor growth in vivo. **a,** Growth dynamic of cells in NSG mice (MDA-MB-231^WT^, MDA-MB-231^EP300KD^ clone 1 and 2, *n* = 5 per cell line), *p*-values (1) - WT vs. EP300 KD clones, (2) – scramble vs. EP300 KD clones. **b,** Representative images of explanted tumors post xenograft culture. **c,** Growth dynamics (4 days) in vitro of MDA-MB-231^WT^ (dotted line) and MDA-MB-231^EP300KD^ (*n* = 3 per cell line). (***p < 0.001, ****p < 0.0001). **d,** Growth curve in vitro of MDA-MB-231^EP300KD^ (dotted line) post xenograft (*n* = 3 per cell line). **e,** Growth dynamic of MDA-MB-231 xenograft in female NSG mice (MDA-MB-231^WT^, EP300 KD clone 1, mixed xenograft (WT and EP300 KD clone 1 mixed 1:1), *n* = 5 mice per cell line. **f,** FACS analysis of cell composition after mixed xenograft experiment (WT – mCherry, EP300 KD – GFP)

While mice bearing xenografts of MDA-MB-231^WT^ and scramble cells developed large tumors within three weeks of cell injection, tumors in MDA-MB-231^EP300KD^ cell xenograft bearing mice barely grew (tumor volume: WT 756.34 ± 58.34 mm^3^ scramble 978.18 ± 155.87 mm^3^, EP300 KD clone 1 31.54 ± 10.67 mm^3^, EP300 KD clone 2 68.17 ± 10.28 mm^3^) (Fig. 3a and b). In contrast, MDA-MB-231^WT^ and EP300 KD cells showed similar growth dynamics in standard 2D monolayer culture in vitro (Fig. 3c). MDA-MB-231^EP300KD^ cells from dissociated tumors after xenograft culture resumed regular growth dynamics in 2D culture (Fig. 3d). FACS based cell cycle analysis demonstrated that both MDA-MB-231^EP300KD^ and scramble transfected cells showed similar cell cycle phase profiles under in vitro conditions. MDA-MB-231^Scramble^ cells isolated after xenograft culture in vivo showed a similar cell cycle profile to in vitro cultured cells, while MDA-MB-231^EP300KD^ cells displayed a cycle profile indicative of a G2/M block after in vivo culture (Supplementary Fig. S2). Altered metabolic conditions more closely mimicking in vivo settings (low glucose, 2% low oxygen) demonstrated no statistically significant difference in growth behavior of MDA-MB-231^EP300KD^ compared to MDA-MB-231^WT^ or scramble cells in vitro (Supplementary Fig. S3). Similar tumor formation was observed in the mixed xenograft (MDA-MB-231^EP300KD^ plus MDA-MB-231^WT^) and MDA-MB-231^WT^ cells only xenografts, without any detection of EP300 KD (GFP positive) cells in the mixed xenograft (tumor volume: WT 1076.04 ± 257.3 mm^3^, mixed xenograft 1178.82 ± 405.16 mm^3^, EP300 KD clone 1 13.5 mm^3^) (Fig. 3e). Post xenograft FACS analysis of dissociated tumors revealed that only mCherry positive (WT) cells were present in the mixed xenograft, indicating that tumor growth was due to wild type cell growth only (Fig. 3f). Taken together, the data showed that EP300 KD inhibited tumor initiation/growth in vivo but did not affect the growth behavior of KD cells in vitro even under conditions partially mimicking in vivo condition.

### EP300 KD reduces migratory and invasive phenotype and circulating tumor cells

**Fig. 4:**
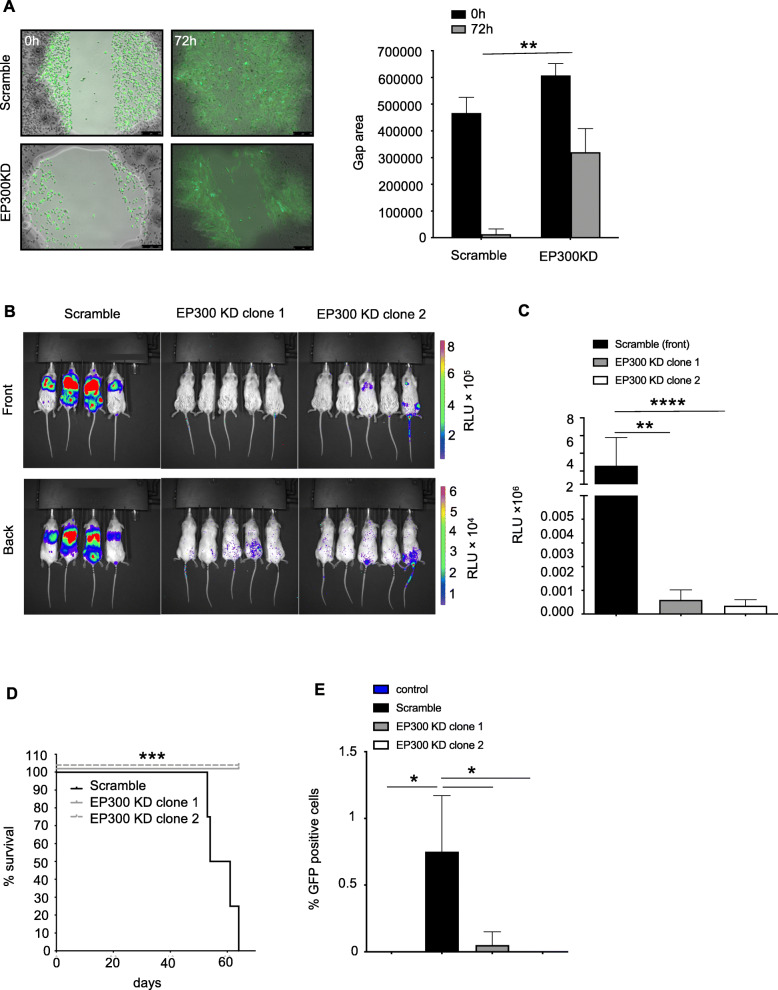
EP300 KD reduces cell metastatic colonization and circulating tumor cells in vivo. **a,** Scratch or wound healing assay comparing MDA-MB-231 scramble transfected and EP300 KD clones (*n* = 6 per cell line). **b**, In vivo bioluminescence images of mice bearing xenografts of MDA-MB-231^Scramble^ and EP300 KD (clone 1 and 2) 5 weeks after tail vein injection (*n* = 4 for scramble, *n* = 5 for EP300 KD clones each). **c,** Quantification of bioluminescence measurements (post 5 weeks). **d,** Survival curve for mice injected via the tail vein with MDA-MB-231^Scramble^ or EP300 KD. **e,** Circulating tumor cell quantification based on GFP expression via FACS in peripheral blood of mice post tail vein injection with MDA-MB-231^Scramble^ or EP300 KD cells (same mice as shown in **d**). (*p < 0.05, **p < 0.01, ***p < 0.001, ****p < 0.0001)

The wound healing assay showed a reduction in gap closure after 72 h in MDA-MBA-231 ^EP300KD^ compared to scramble transfected controls as a surrogate for reduced migratory potential (gap area 0 h vs. 72 h: scramble 466,457 ± 58,776 vs. 12,898 ± 20,148, MDA-MBA-231 ^EP300KD^ 607,539 ± 44,278 vs. 320,399 ± 88,116, *p* = 0.0017) (Fig. 4a). After tail vein injection, MDA-MB-231^EP300KD^ cells expressing GFP/luciferase showed no tumor formation in the lungs or other parts of the mouse body in vivo compared to large tumor masses (as determined by fluorescence signal intensity) in the control (scramble) group (RLU × 10^5^: scramble 2.6 ± 3.19, EP300 KD clone 1 0.006 ± 0.004, EP300 KD clone 2 0.004 ± 0.003) (Fig. 4b and c) (MDA-MB-231^EP300KD^ clone 1 vs. scramble *p* = 0.005, clone 2 vs. scramble *p* > 0.0001). While all mice in the control group show significant signs of sickness, including low body weight and were sacrificed after 57.5 ± 5.4 days, mice injected with EP300 KD clones showed no sign of disease (Fig. 4d and supplementary Fig. S4) (*p* = 0.0009). Post necropsy, blood samples of all mice were investigated for the presence of circulating tumor cells based on the presence of GFP-positive cancer cells in FACS analysis. While scrambled xenografted mice had 0.75 ± 0.42% GFP-positive cells in circulation, only 0.05 ± 0.1 and 0% were found in MDA-MB-231^p300KD^ xenograft mice from clone 1 and 2, respectively (Fig. 4e) (*p* < 0.05). The results showed the EP300 KD abolishes metastatic colonization and spread in the form of circulating tumor cells in TNBC cells.

### EP300 correlated genes show CSC related transcription in TNBC and basal-like BC

**Fig. 5:**
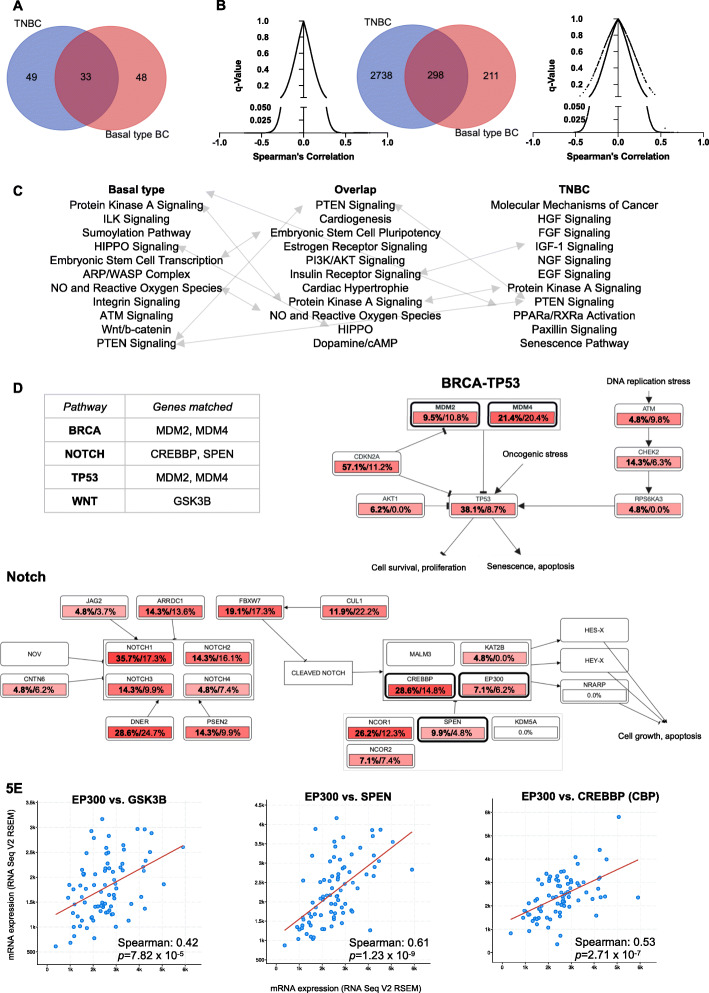
EP300 expression is correlated with CSC gene expression in the TCGA BC cohort. **a,** Overlap between TNBC (*n* = 82) and basal-like BC (*n* = 81). **b,** Number of genes positively correlated with EP300 in basal-like BC (*n* = 509) and TNBC (*n* = 3036) and overlap of genes (*n* = 298) (*q*-value < 0.05**). c,** Biological functions and pathway associated with the significantly correlated genes (IPA analysis), ranked by *p-*value (range 1.74 × 10^− 4^ to 1.34 × 10^− 3^). Grey arrows indicate overlapping functions between gene lists. **d,** Affected biological functions and pathways (BRCA/TP53, Notch) associated with EP300 correlated genes that overlap in TNBC and basal BC (*n* = 298) (cBioPortal). Affected genes are outlined in bold black. Percentages represent the proportion of samples which show alterations in pathway genes (bold – TNBC cases, regular font – basal-like BC). **e,** Bivariate plots of genes in affected pathways **d** that are positively correlated with EP300 expression (RSEM – RNA-Seq by Expectation-Maximization)

The TCGA breast cancer cohort was filtered for TNBC (*n* = 82) or basal-like breast cancers (*n* = 81), which based on gene expression showed an overlap of *n* = 33 cases between both sample groups (Fig. 5a). Gene expression demonstrated that *n* = 3037 and *n* = 509 genes in TNBC and basal-like BC, respectively, correlated positively with EP300 expression (FDR < 0.05), with *n* = 298 genes correlating in both subgroups (Fig. 5b and supplementary Table S1). The top associated pathways and biological functions in Ingenuity Pathway Analysis (IPA) (Qiagen, Hilden, Germany) are shown in Fig. 5b. While genes correlating with EP300 in basal breast cancer were associated with stem cell biology and cancer metastasis, EP300 correlated genes in TNBC were associated with growth factor signaling pathways (Fig. 5c). All three sets of genes positively correlated with EP300 (TNBC, basal-like BC and the overlap between both) were linked to PTEN and Protein kinase A (PKA) signaling (Fig. 5c).

Using the overlap gene signature (*n* = 298 genes) to analyze associated biological function via cBioPortal showed that the top associated pathways were TP53/BRCA, Notch and Wnt signaling (Fig. 5d and supplementary Fig. S5). The expression of affected genes in these pathways (Notch – CREBBP, SPEN; Wnt – GSK3B) showed strong positive correlation with EP300 expression on a per sample basis (Fig. 5e). The graphs for Fig. 5d and e were created and directly exported form cBioPortal [25]. These results demonstrated that EP300 expression correlated with CSC related genes and pathways (Notch, Wnt, BRCA/TP53) suggesting at complex role of EP300 in CSC biology.

### EP300 is prognostic in TNBC and basal-like BC

**Fig. 6:**
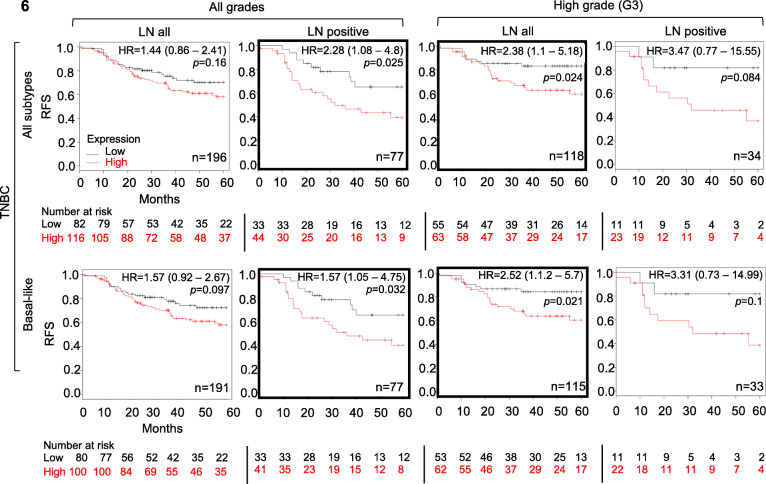
EP300 expression predicts recurrence free survival: Kaplan-Meier analysis showing RFS in EP300 high vs. low expressing TNBC and basal BC patients in association with tumor grade (G3 – high grade vs. all grades), LN metastasis (LN all vs. LN metastasis) and PAM50 subtypes (all vs. basal-like TNBC) (bold black framed Kaplan-Meier plots with *p* < 0.05)

The association between EP300 expression and RFS was assessed in 6234 breast cancer patients, of which 198 TNBC cases were available for analysis. High EP300 gene expression was statistically significantly correlated with worse RFS in TNBC and basal-like BC patients with lymph node metastasis or high-grade tumors (G3) (Fig. 6). Although not all results reached statistical significance, there was a clear trend towards increasing hazard ratios (HR) associated with higher histological grade (G3) and lymph node (LN) metastasis, with the highest HR in TNBC G3 and positive LN metastasis (Fig. 6). Comparing all PAM50 subtypes to basal-like BC had no significant effect on recurrence free survival. Of note, while higher EP300 RNA expression was associated with a better prognosis in BC in general (Supplementary Fig. S6A), high EP300 protein expression demonstrated worse RFS (HR 3.32, 1.53–7.19) in all BC patients (Supplementary Fig. S6B). The results demonstrated that high EP300 RNA expression might function as prognostic marker for poor RFS in TNBC/basal-like breast cancer.

## Discussion

We demonstrated that knockdown of EP300 reduced the expression of ABCG2, eliminated side population cells and decreased tumor initiating as well as metastatic potential. EP300 gene expression in large published breast cancer data sets positively corresponded with genes and molecular pathways associated with CSC function and predicted poor RFS in TNBC and basal-like BC. Collectively these data demonstrated a novel oncogenic role for EP300 in TNBC and basal-like breast cancer biology.

ABCG2 (also known as the breast cancer resistance protein – BCRP) plays a pivotal role in drug resistance (e.g. anthracyclines and topoisomerase inhibitors) and as important marker of side population cells, which are enriched for cells with CSC characteristics in breast cancer [27, 28]. Our study showed a correlation between reduction in ABCG2 expression and side population phenotype. Patrawala et al. showed that while the side population was enriched for tumorigenic CSC-like cells, ABCG2 levels did not affect their tumorigenic potential [28]. Other BC-CSC markers and phenotypes have been described (e.g. EPCAM^+^CD44^+^CD24^−^ phenotype [11], SOX2 [29], and ALDH1 [30]) and it would be of interest to investigate the effect of EP300 KD on these cell populations. Nevertheless, functional characterization (i.e. tumor initiation) of CSCs should be considered the gold standard as limited marker profiles may identify distinct subpopulations and not fully capture functional CSC [31].

We observed a stark difference between regular 2D culture and 3D tumorsphere as well as in vivo tumor formation. The microenvironment plays a critical role in tumor development via paracrine signaling or physical factors. Stromal cells can function as both tumor suppressors or promoters, depending on context [32–34]. Although xenograft co-culture with MDA-MB-231^WT^ or oxygen and glucose levels did not affect MDA-MB-231^EP300KD^ growth in vivo or in vitro, respectively, it is conceivable that the complex interplay between cancer cells, in particular CSC and the tumor environment may affect growth and tip the balance towards the suppression of tumor outgrowth after EP300 knockdown. EP300 has been shown to affect tumor-propagating physical properties [35], in concordance with the observed difference in 2D adherent culture and 3D suspension culture in the current study. Differences we observed in tumorsphere formation capacity after EP300 KD between different TNBC cell lines could be due to technical differences in gene knock down (stable vs. transient) or have distinct biological reasons such as the claudin-low versus basal-like subtypes [36].

Since we used an immunocompromised (NSG) mouse model, immunological factors are unlikely to play a role in the absence of in vivo tumor formation through EP300 KD. Nevertheless, Xiong et al. showed that EP300 expression might be predictive of hyper progressive disease after PD-L1 blockade [37] and it would be of interest to investigate the role of EP300 in TNBC cells in the context of a functional immune system [38].

In agreement with findings that EP300 increases migratory potential and invasiveness of cancer cells [39, 40], we demonstrated reduced lung colonization and distant metastasis as well as circulating tumor cells via EP300 KD in TNBC cells.

Based on our findings, future studies should investigate whether EP300 targeted drugs (flufenamic acid [18], VV59 [19], A-485 [41]) can inhibit tumor initiation and metastasis in TNBC pre-clinical models.

TCGA analysis of TNBC and basal-like BC samples showed that pathways associated with CSC biology (integrin and integrin-linked kinase (ILK) signaling [42], Wnt/ß-catenin [43], actin-ARP-WASP [44], Notch signaling [45], BRCA [46] and embryonic stem cells (ES) transcriptional networks) were positively correlated with EP300 expression. Specific genes, such as PTEN, protein kinase A (PKA), GSK3B, CREBBP, SPEN have been linked to CSC biology and metastasis in breast cancer [47–51]. Since EP300 is an important epigenetic regulator via histone modifications, further analysis of the relationship between CSC gene expression and histone acetylation through EP300 are warranted.

Our survival analysis in-silico demonstrated worse RFS correlated with high EP300 expression in TNBC and basal-like BC patients with high grade (G3) or LN metastasized tumors. Although not statistically significant due to small patient numbers, there was a trend toward increased hazard ratios for patients with both high grade and LN metastasized tumors.

Naturally there are limitations to our study. The fact that only a single cell line was used for in vivo studies, as well as the use of immunocompromised mice limits the extrapolation of our results to the complex conditions in individual BC patients. The effect of the immune system on tumor growth is one of the most exciting and promising area of cancer research and the effect of EP300 expression in this context should be explored. The role of the tumor microenvironment more generally seems to play a critical role in the findings described here and invites further detailed experimentation. EP300 has been found to have tumor suppressor [16, 17] as well as an oncogenic properties [18, 19]. Another famous tumor suppressor, TP53, has been shown to potentially have pro-tumorigenic effects via increased inflammation [52] or anti-apoptotic mechanisms [53]. Collectively, these findings raise the possibility that context matters.

## Conclusion

The data presented here is to our knowledge the first study describing a context-depended oncogenic role of EP300 in CSC phenotype in TNBC and basal-like breast cancer. Knockdown of EP300 eliminated CSCs in vitro and decreased tumor initiating as well as metastatic potential in vivo. Large breast cancer gene expression data sets revealed that EP300 expression is positively correlated with the expression of CSC markers and poor prognosis in TNBC and basal-like BC. Our results warrant further exploration of EP300 as a prognostic factor and potential therapeutic target in TNBC and basal-like BC.

## Supplementary Information

**Additional file 1 Supplementary Table S1:** Genes positively correlated with EP300 (*q* < 0.05) in TNBC and basal-like breast cancer in the TCGA BC cohort (pdf file attachment). **Supplementary Table S2.** Genes and primer sequences for qPCR. **Supplementary Figure S1**. FACS analysis of CD44 and cD24 expression in MDA-MB-231 scramble transfected as well as 2 EP300 KD clones. **Supplementary Figure S2.** Cell cycle analysis of MDA-MB-231^Scramble^ and EP300 KD after regular in vitro 2D monolayer culture (top panel) and after xenograft in mice (bottom panel) (*n* = 1 per condition per cell type). **Supplementary Figure S3.** Cell count of MDA-MB-231^WT^, scramble and EP300 KD cells (clone 1 and 2) for 24, 48 and 72 h culture under low glucose and low (2%) oxygen conditions (*n* = 3 per cell type per time point). **Supplementary Figure S4.** Mouse weight at the end of tail vein injection xenograft experiment. **Supplementary Figure S5.** Affected biological pathway (WNT signaling) associated with EP300 correlated genes that overlap in TNBC and basal BC (*n* = 298) (cBioPortal). Affected genes are outlined in bold black. Percentages represent the proportion of samples which show alterations in pathway genes (bold – TNBC cases, regular font – basal-like BC). **Supplementary Figure S6.** Prognostic value (PFS and HR) of EP300 gene and protein expression in BC. **A,** EP300 gene expression in BC patients irrespective of subtype, receptor expression, grade and LN status. **B,** EP300 protein expression in BC patients irrespective of subtype, receptor expression, grade and LN status.

## Data Availability

The gene expression datasets analyzed during the current study are publicly available in the cBioPortal and KM plotter as referenced in the manuscript. All data generated or analyzed during this study are included in this published article (and its Supplementary Information files).

## Abbreviations

ABCG2: ATP-binding cassette super-family G member 2
CSC: Cancer Stem Cells
EP300: E1A Binding Protein P300
FACS: Fluorescence Activated Cell Sorting
GFP: Green Fluorescent Protein
KD: Knockdown
NOD: Non-Obese Diabetic
RFS: Recurrence Free Survival
RLU: Relative Light Unit
TCGA: The Cancer Genome Atlas
SCID: Severe Combined Immunodeficiency
TNBC: Triple Negative Breast Cancer
