# Supplementary material for: EP300 knockdown reduces cancer stem cell phenotype, tumor growth and metastasis in triple negative breast cancer
Source: BMC Cancer. 2020 Nov 10;20:1076. doi: 10.1186/s12885-020-07573-y (PMC7653866; doi:10.1186/s12885-020-07573-y)
Supplement: Supplementary file 1 — Additional file 1 Supplementary Table S1: Genes positively correlated with EP300 (q < 0.05) in TNBC and basal-like breast cancer in the TCGA BC cohort (pdf file attachment). Supplementary Table S2. Genes and primer sequences for qPCR. Supplementary Figure S1. FACS analysis of CD44 and cD24 expression in MDA-MB-231 scramble transfected as well as 2 EP300 KD clones. Supplementary Figure S2. Cell cycle analysis of MDA-MB-231Scramble and EP300 KD after regular in vitro 2D monolayer culture (top panel) and after xenograft in mice (bottom panel) (n = 1 per condition per cell type). Supplementary Figure S3. Cell count of MDA-MB-231WT, scramble and EP300 KD cells (clone 1 and 2) for 24, 48 and 72 h culture under low glucose and low (2%) oxygen conditions (n = 3 per cell type per time point). Supplementary Figure S4. Mouse weight at the end of tail vein injection xenograft experiment. Supplementary Figure S5. Affected biological pathway (WNT signaling) associated with EP300 correlated genes that overlap in TNBC and basal BC (n = 298) (cBioPortal). Affected genes are outlined in bold black. Percentages represent the proportion of samples which show alterations in pathway genes (bold – TNBC cases, regular font – basal-like BC). Supplementary Figure S6. Prognostic value (PFS and HR) of EP300 gene and protein expression in BC. A, EP300 gene expression in BC patients irrespective of subtype, receptor expression, grade and LN status. B, EP300 protein expression in BC patients irrespective of subtype, receptor expression, grade and LN status. [file 12885_2020_7573_MOESM1_ESM.zip › Additional figure 1R3.pdf]

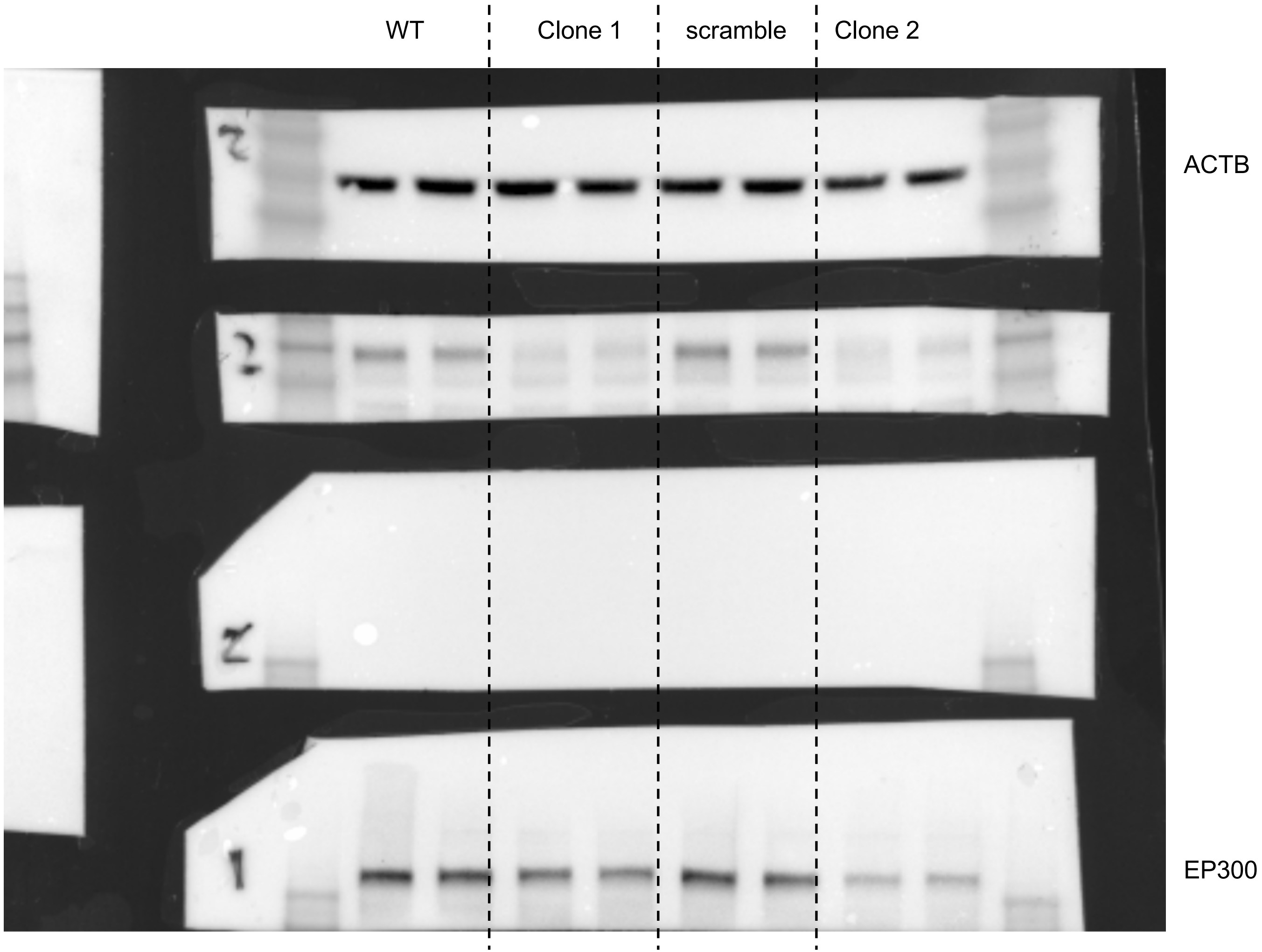

Original WB for Figure 1A: Samples were prepared in technical duplicates (separeated by dotted lines)
