# Supplementary material for: EP300 knockdown reduces cancer stem cell phenotype, tumor growth and metastasis in triple negative breast cancer
Source: BMC Cancer. 2020 Nov 10;20:1076. doi: 10.1186/s12885-020-07573-y (PMC7653866; doi:10.1186/s12885-020-07573-y)
Supplement: Supplementary file 1 — Additional file 1 Supplementary Table S1: Genes positively correlated with EP300 (q < 0.05) in TNBC and basal-like breast cancer in the TCGA BC cohort (pdf file attachment). Supplementary Table S2. Genes and primer sequences for qPCR. Supplementary Figure S1. FACS analysis of CD44 and cD24 expression in MDA-MB-231 scramble transfected as well as 2 EP300 KD clones. Supplementary Figure S2. Cell cycle analysis of MDA-MB-231Scramble and EP300 KD after regular in vitro 2D monolayer culture (top panel) and after xenograft in mice (bottom panel) (n = 1 per condition per cell type). Supplementary Figure S3. Cell count of MDA-MB-231WT, scramble and EP300 KD cells (clone 1 and 2) for 24, 48 and 72 h culture under low glucose and low (2%) oxygen conditions (n = 3 per cell type per time point). Supplementary Figure S4. Mouse weight at the end of tail vein injection xenograft experiment. Supplementary Figure S5. Affected biological pathway (WNT signaling) associated with EP300 correlated genes that overlap in TNBC and basal BC (n = 298) (cBioPortal). Affected genes are outlined in bold black. Percentages represent the proportion of samples which show alterations in pathway genes (bold – TNBC cases, regular font – basal-like BC). Supplementary Figure S6. Prognostic value (PFS and HR) of EP300 gene and protein expression in BC. A, EP300 gene expression in BC patients irrespective of subtype, receptor expression, grade and LN status. B, EP300 protein expression in BC patients irrespective of subtype, receptor expression, grade and LN status. [file 12885_2020_7573_MOESM1_ESM.zip › Supplementary table S1R3.pdf]

TCGA basal-like BC

Correlated G	Cytoband	Spearman's (	p-Value	q-Value
ZNF654	3p11.1	0.6995901	3.71E-13	6.34E-09
JUN	1p32.1	0.6454607	7.76E-11	6.63E-07
THAP6	4q21.1	0.62938121	3.11E-10	1.77E-06
RASA2	3q23	0.6098916	1.51E-09	5.6143E-06
ANKRD11	16q24.3	0.60751246	1.81E-09	5.6143E-06
ZBTB38	3q23	0.60643631	1.97E-09	5.6143E-06
NCOA3	20q13.12	0.60006775	3.21E-09	7.8321E-06
REST	4q12	0.59566396	4.47E-09	8.3513E-06
CLIP1	12q24.31	0.5941655	4.99E-09	8.3513E-06
SPATA13	13q12.12	0.59366866	5.18E-09	8.3513E-06
ARFGAP3	22q13.2	0.59268293	5.57E-09	8.3513E-06
AKAP13	15q25.3	0.59198284	5.87E-09	8.3513E-06
ZEB1	10p11.22	0.59019874	6.69E-09	8.7868E-06
MAP3K1	5q11.2	0.58824858	7.71E-09	9.0785E-06
NFIC	19p13.3	0.58778561	7.97E-09	9.0785E-06
PPP1R12A	12q21.2-q21	0.58401084	1.05E-08	1.0958E-05
RC3H1	1q25.1	0.58313008	1.11E-08	1.0958E-05
ACIN1	14q11.2	0.58263324	1.15E-08	1.0958E-05
PACS1	11q13.1-q13	0.57958446	1.43E-08	1.2887E-05
EFCAB14	1p33	0.57878601	1.52E-08	1.2952E-05
ZNF800	7q31.33	0.57565492	1.89E-08	1.5358E-05
RBAK	7p22.1	0.57328365	2.23E-08	1.728E-05
ZNF669	1q44	0.57190605	2.45E-08	1.8176E-05
CRCP	7q11.21	0.57093496	2.62E-08	1.8619E-05
TCF25	16q24.3	0.56932345	2.92E-08	1.885E-05
SYNE2	14q23.2	0.56901536	2.98E-08	1.885E-05
MACF1	1p34.3	0.56847335	3.09E-08	1.885E-05
LIMS1	2q12.3	0.56838623	3.11E-08	1.885E-05
PPP1R3D	20q13.33	0.56797972	3.20E-08	1.885E-05
CEP290	12q21.32	0.5668957	3.44E-08	1.9511E-05
MIA2	14q21.1	0.566486	3.54E-08	1.9511E-05
RORA	15q22.2	0.56583107	3.70E-08	1.9755E-05
SRSF4	1p35.3	0.56268385	4.57E-08	2.3657E-05
FRYL	4p11	0.56135141	4.99E-08	2.5091E-05
ZKSCAN1	7q22.1	0.55883017	5.90E-08	2.8796E-05
PRKG1	10q11.23-q2	0.55250989	8.91E-08	4.1363E-05
FICD	12q23.3	0.55230352	9.03E-08	4.1363E-05
YY1	14q32.2	0.55176152	9.35E-08	4.1363E-05
RAD23A	19p13.13	0.55160343	9.44E-08	4.1363E-05
ARHGAP5	14q12	0.55051942	1.01E-07	4.3235E-05
ZNF766	19q13.41	0.54995483	1.05E-07	4.3734E-05
DYNC1LI2	16q22.1	0.5467028	1.29E-07	5.2508E-05
CHD9	16q12.2	0.53882114	2.11E-07	8.2251E-05
SETD2	3p21.31	0.53877597	2.12E-07	8.2251E-05
BDP1	5q13.2	0.53796296	2.23E-07	8.4554E-05
WHAMMP3	15q11.2	0.53532069	2.62E-07	9.7251E-05

PLCB1	20p12.3	0.53383017	2.87E-07	0.00010233
FAM111B	11q12.1	0.533785	2.88E-07	0.00010233
C5ORF24	5q31.1	0.53105239	3.39E-07	0.00011824
SCAF11	12q12	0.52917796	3.80E-07	0.00012967
PPP1R12B	1q32.1	0.52850045	3.95E-07	0.00013237
ASH1L	1q22	0.52646793	4.46E-07	0.00014651
MAN1A2	1p12	0.52346432	5.32E-07	0.00017162
NFAT5	16q22.1	0.52225905	5.71E-07	0.00018078
DENND1B	1q31.3	0.51982001	6.59E-07	0.00020097
KAT6B	10q22.2	0.51955736	6.69E-07	0.00020097
CASC4	15q15.3	0.5195122	6.71E-07	0.00020097
STX6	1q25.3	0.51715513	7.68E-07	0.00022248
TAOK1	17q11.2	0.51556007	8.42E-07	0.00023975
CFAP44	3q13.2	0.51443089	8.98E-07	0.00025154
REL	2p16.1	0.51296296	9.76E-07	0.00026905
KLHL20	1q25.1	0.51133695	1.0707E-06	0.00028866
XRN1	3q23	0.51099819	1.0914E-06	0.00028866
CUEDC1	17q22	0.51088528	1.0983E-06	0.00028866
TUG1	22q12.2	0.50946251	1.1899E-06	0.00030799
FCHO2	5q13.2	0.50794941	1.2952E-06	0.00033023
CYB5R3	22q13.2	0.50740741	1.335E-06	0.00033537
MIER1	1p31.3	0.50426829	1.5891E-06	0.00038781
SMAD5	5q31.1	0.50374887	1.6353E-06	0.00039347
PAWR	12q21.2	0.50261969	1.7402E-06	0.00040723
DLGAP4	20q11.23	0.50160343	1.84E-06	0.00042476
KIAA1109	4q27	0.50092593	1.9095E-06	0.00043094
EIF2AK4	15q15.1	0.50079042	1.9237E-06	0.00043094
MTF1	1p34.3	0.50061258	1.9424E-06	0.00043094
ZC3H13	13q14.13	0.49997742	2.0109E-06	0.00043579
OSBPL8	12q21.2	0.49973182	2.038E-06	0.00043579
SLX4IP	20p12.2	0.49719964	2.338E-06	0.00049309
PURA	5q31.3	0.49679313	2.3899E-06	0.00049788
TAS2R50	12p13.2	0.49593776	2.5026E-06	0.00051462
RYBP	3p13	0.49573171	2.5305E-06	0.00051462
ZBTB7A	19p13.3	0.49467028	2.6788E-06	0.00053083
VANGL1	1p13.1	0.49448961	2.7049E-06	0.00053083
OXR1	8q23.1	0.49442186	2.7147E-06	0.00053083
GOLGA2P5	12q23.1	0.49428636	2.7345E-06	0.00053083
VASP	19q13.32	0.4938121	2.8047E-06	0.00053835
ZC3H7A	16p13.13	0.49342818	2.8628E-06	0.00054329
CARF	2q33.2	0.49322493	2.8941E-06	0.00054329
PRRC1	5q23.2	0.49257001	2.9968E-06	0.0005538
PPIG	2q31.1	0.491757	3.1292E-06	0.00056608
ITGA6	2q31.1	0.49051491	3.3421E-06	0.00059472
CCDC125	5q13.2	0.48827913	3.7601E-06	0.00065386
SLC35E4	22q12.2	0.48809846	3.7959E-06	0.00065386
UTP23	8q24.11	0.48794038	3.8275E-06	0.00065386
GOLIM4	3q26.2	0.48771454	3.8731E-06	0.0006551

SPTLC2	14q24.3	0.48641874	4.1448E-06	0.00069417
EZR	6q25.3	0.48615628	4.202E-06	0.00069692
CCDC141	2q31.2	0.48414634	4.665E-06	0.00075897
DAAM1	14q23.1	0.48299458	4.9514E-06	0.00079052
MPP5	14q23.3	0.48220416	5.1575E-06	0.00081579
NIPBL	5p13.2	0.48037489	5.6657E-06	0.00088538
L3MBTL2	22q13.2	0.47922313	6.0094E-06	0.00092326
RBX1	22q13.2	0.47901987	6.0721E-06	0.00092326
ITGA8	10p13	0.47890696	6.1071E-06	0.00092326
GNPTG	16p13.3	0.47825203	6.3143E-06	0.00094621
KDM4A	1p34.2-p34.1	0.47721319	6.6565E-06	0.00098881
CCDC186	10q25.3	0.47391599	7.8615E-06	0.00115774
SMG1	16p12.3	0.47323848	8.1331E-06	0.00118751
MFSD6	2q32.2	0.47227006	8.5368E-06	0.0012312
SESTD1	2q31.2	0.47217706	8.5765E-06	0.0012312
ALDH9A1	1q24.1	0.47199639	8.6542E-06	0.001232
IRF2BP2	1q42.3	0.47168022	8.7918E-06	0.00124124
ZNF644	1p22.2	0.47136405	8.9313E-06	0.00125061
MAGI3	1p13.2	0.471107	9.0464E-06	0.00125642
AP3D1	19p13.3	0.47057362	9.2895E-06	0.00126954
SCLT1	4q28.2	0.46948961	9.8025E-06	0.00132902
TECPR1	7q21.3	0.46917609	9.9558E-06	0.00133917
TOB2	22q13.2	0.46894761	1.0069E-05	0.0013438
ANKIB1	7q21.2	0.46847335	1.0307E-05	0.00136498
FOXN2	2p16.3	0.46616983	1.1544E-05	0.00146741
USP37	2q35	0.46601174	1.1633E-05	0.00146741
ZBTB37	1q25.1	0.46600308	1.1638E-05	0.00146741
USP10	16q24.1	0.46596658	1.1659E-05	0.00146741
ANKRD36	2q11.2	0.46587624	1.1711E-05	0.00146741
ZMAT3	3q26.32	0.46569557	1.1815E-05	0.00146741
ZNF441	19p13.2	0.46562782	1.1854E-05	0.00146741
XPNPEP3	22q13.2	0.46506323	1.2186E-05	0.00149761
PACSIN2	22q13.2	0.4633243	1.3262E-05	0.0016183
IGF2R	6q25.3	0.4629178	1.3527E-05	0.00162657
PALLD	4q32.3	0.46287263	1.3556E-05	0.00162657
LRRC58	3q13.33	0.46226287	1.3963E-05	0.00165643
F2R	5q13.3	0.46205962	1.4101E-05	0.00166126
RGPD4	2q12.3	0.46163053	1.4396E-05	0.00168446
HECTD4	12q24.13	0.46066204	1.5085E-05	0.00174116
PRKCI	3q26.2	0.4597561	1.5756E-05	0.00179268
DNAJC3	13q32.1	0.45964318	1.5842E-05	0.00179268
SYDE2	1p22.3	0.45941734	1.6015E-05	0.00179268
TAF2	8q24.12	0.45932701	1.6084E-05	0.00179268
UBR1	15q15.2	0.45932701	1.6084E-05	0.00179268
LPP	3q27.3-q28	0.45922798	1.6161E-05	0.00179268
ICK	6p12.1	0.45858175	1.6669E-05	0.00182533
KMT2A	11q23.3	0.45858175	1.6669E-05	0.00182533
STPG4	2p21	0.45785296	1.7259E-05	0.00187798

CCDC174	3p25.1	0.45727191	1.7744E-05	0.00191853
YTHDC2	5q22.2	0.45616531	1.8703E-05	0.00200553
TOR1AIP1	1q25.2	0.45607498	1.8784E-05	0.00200553
RIF1	2q23.3	0.45551039	1.9294E-05	0.00204544
NKTR	3p22.1	0.45539747	1.9397E-05	0.00204544
BOD1L1	4p15.33	0.4548103	1.9944E-05	0.002087
CALCRL	2q32.1	0.45460705	2.0136E-05	0.002087
HIST1H4C	6p22.2	0.45458446	2.0158E-05	0.002087
OTUD3	1p36.13	0.45381662	2.0902E-05	0.00215097
B3GALNT2	1q42.3	0.45275519	2.1972E-05	0.00223897
SEC63	6q21	0.45271003	2.2019E-05	0.00223897
DDX17	22q13.1	0.45252936	2.2206E-05	0.00224468
ZFP90	16q22.1	0.45219061	2.2562E-05	0.00226723
MTPN	7q33	0.45135501	2.3463E-05	0.0023303
MINDY2	15q21.3-q22	0.45099368	2.3862E-05	0.0023563
CTCF	16q22.1	0.45067751	2.4217E-05	0.00236402
ANKRD50	4q28.1	0.44827489	2.708E-05	0.00261358
MYO1C	17p13.3	0.44803523	2.7382E-05	0.00262417
SYNJ2BP	14q24.2	0.4479449	2.7497E-05	0.00262417
HIST3H3	1q42.13	0.44744806	2.8136E-05	0.00267024
POLH	6p21.1	0.44715447	2.852E-05	0.00269174
ILDR1	3q13.33	0.44688347	2.8879E-05	0.00269582
ZHX2	8q24.13	0.44674797	2.906E-05	0.00269798
AHCTF1	1q44	0.4465924	2.9269E-05	0.00270271
SLC25A36	3q23	0.44360885	3.3561E-05	0.00305507
RBM25	14q24.2	0.44336043	3.3944E-05	0.00305507
CSPP1	8q13.1-q13.2	0.44252484	3.5261E-05	0.00315348
MYCBP2	13q22.3	0.44241192	3.5443E-05	0.00315348
FAM126B	2q33.1	0.44218609	3.5809E-05	0.00316952
RANBP2	2q13	0.44126016	3.7345E-05	0.00327165
TMX4	20p12.3	0.44087624	3.8001E-05	0.00330875
ALKBH8	11q22.3	0.44078591	3.8156E-05	0.00330875
ZNF451	6p12.1	0.44040199	3.8825E-05	0.00333316
MIB1	18q11.2	0.4403794	3.8864E-05	0.00333316
ZBTB43	9q33.3	0.44028907	3.9023E-05	0.00333316
SF1	11q13.1	0.43974706	3.999E-05	0.00337899
HOOK1	1p32.1	0.43965673	4.0153E-05	0.00337899
TMF1	3p14.1	0.43929539	4.0812E-05	0.00341763
SRGAP1	12q14.2	0.43877597	4.1778E-05	0.0034645
CEP63	3q22.2	0.43861789	4.2076E-05	0.00347235
GTF2A1	14q31.1	0.43726287	4.4712E-05	0.00367219
MBNL1	3q25.1-q25.2	0.43635953	4.6554E-05	0.00377915
UACA	15q23	0.43631436	4.6648E-05	0.00377915
DCBLD1	6q22.1	0.4362692	4.6742E-05	0.00377915
MIS18BP1	14q21.2	0.43608853	4.712E-05	0.00377915
PDXDC2P-NP	16q22.1	0.43466576	5.02E-05	0.0040073
P2RY1	3q25.2	0.43240741	5.5474E-05	0.00438734
CNN2	19p13.3	0.43100723	5.8998E-05	0.00463825

ANKRD12	18p11.22	0.43084914	5.9409E-05	0.00463825
SLFN5	17q12	0.43037489	6.0657E-05	0.00471
GNPTAB	12q23.2	0.43012647	6.132E-05	0.00473996
NEXN	1p31.1	0.42947154	6.3101E-05	0.00485569
UBXN4	2q21.3	0.42931588	6.3532E-05	0.00486688
CCDC47	17q23.3	0.42920054	6.3853E-05	0.00486961
HIST1H4L	6p22.1	0.42824316	6.6573E-05	0.00497406
ZBTB41	1q31.3	0.42820687	6.6678E-05	0.00497406
RBFOX2	22q12.3	0.42734869	6.9211E-05	0.00514058
HMGB1P1	20q13.31	0.42721319	6.9619E-05	0.0051485
CDC42BPA	1q42.13	0.42653568	7.1693E-05	0.00525635
PDPK1	16p13.3	0.42592593	7.3608E-05	0.00537372
BAGE2	21p11.2	0.42475158	7.7431E-05	0.00560491
GPATCH11	2p22.2	0.42463866	7.7809E-05	0.00560845
GIGYF2	2q37.1	0.42420958	7.9257E-05	0.00566507
RALGAPA1	14q13.2	0.42366757	8.1123E-05	0.00575031
MYH9	22q12.3	0.4233514	8.223E-05	0.0058047
KIAA0319L	1p34.3	0.4230804	8.319E-05	0.0058483
SYTL2	11q14.1	0.42050587	9.2843E-05	0.00647983
CNOT6L	4q21.1	0.42048329	9.2932E-05	0.00647983
SPOP	17q21.33	0.42021229	9.4007E-05	0.00652815
TSPO	22q13.2	0.41933389	9.7572E-05	0.00674827
TCF12	15q21.3	0.41906052	9.8707E-05	0.00679783
HDGF	1q23.1	0.41897019	9.9084E-05	0.00679783
CD164	6q21	0.41829504	0.00010195	0.00696641
TNRC6A	16p12.1	0.41797651	0.00010333	0.00703245
TULP4	6q25.3	0.41768293	0.00010461	0.00709169
RBM22	5q33.1	0.41752484	0.00010531	0.00711081
DDX6	11q23.3	0.41725384	0.00010652	0.00715626
SORL1	11q24.1	0.41693767	0.00010794	0.00720309
RNF13	3q25.1	0.41657633	0.00010959	0.00728471
ZNRF3	22q12.1	0.41596658	0.00011243	0.00744311
SAFB2	19p13.3	0.41578591	0.00011328	0.00744311
MBTD1	17q21.33	0.41492773	0.00011742	0.00765599
SREBF2	22q13.2	0.41402439	0.00012192	0.007919
EXT1	8q24.11	0.41391147	0.0001225	0.007919
FRMD4B	3p14.1	0.41384372	0.00012284	0.007919
MED13	17q23.2	0.41368564	0.00012365	0.00794126
PDP1	8q22.1	0.4131888	0.00012623	0.00801236
ZC3H7B	22q13.2	0.41303305	0.00012705	0.00801236
PHC3	3q26.2	0.41299917	0.00012723	0.00801236
ZC3H18	16q24.2	0.41298555	0.0001273	0.00801236
FAM215A	17q21.31	0.41293375	0.00012757	0.00801236
MIR-140/5P		0.55043793	0.00013118	0.008201
PTPRE	10q26.2	0.41219512	0.00013154	0.008201
TACC1	8p11.22	0.41174345	0.00013402	0.00829504
RFTN2	2q33.1	0.41081752	0.00013924	0.00855606
TOR1AIP2	1q25.2	0.41052857	0.0001409	0.00862744

SRP72	4q12	0.40991418	0.00014451	0.00881658
CCDC191	3q13.31	0.40982385	0.00014505	0.00881786
KIAA0040	1q25.1	0.40941734	0.00014749	0.00893441
OAZ1	19p13.3	0.40907859	0.00014955	0.00902056
BBS4	15q24.1	0.40885276	0.00015094	0.00903481
IBTK	6q14.1	0.40790425	0.00015691	0.00930705
GSK3B	3q13.33	0.40688799	0.00016354	0.00963066
HELB	12q14.3|12q	0.40681124	0.00016405	0.00963066
FAM172A	5q15	0.40643631	0.00016657	0.00972075
CEP97	3q12.3	0.40641373	0.00016673	0.00972075
SRRM1	1p36.11	0.40605239	0.00016919	0.00983089
WWC1	5q34	0.40404246	0.00018352	0.01062755
RPS6KB1	17q23.1	0.40386179	0.00018486	0.01065253
MON2	12q14.1	0.40381662	0.0001852	0.01065253
SNAI2	8q11.21	0.4035682	0.00018706	0.01072356
RALGAPA2	20p11.23	0.40180668	0.00020078	0.0114711
BBX	3q13.12	0.4016486	0.00020205	0.01150545
GLG1	16q23.1	0.40121951	0.00020555	0.01166574
JAK1	1p31.3	0.40036134	0.00021271	0.01199261
SNRK	3p22.1	0.39993225	0.00021638	0.01211937
ZFC3H1	12q21.1	0.39993225	0.00021638	0.01211937
KIF3B	20q11.21	0.39983288	0.00021724	0.01212764
FAM161A	2p15	0.39916441	0.00022309	0.01233335
TSHZ2	20q13.2	0.39887082	0.0002257	0.01240879
INSIG1	7q36.3	0.39884824	0.0002259	0.01240879
RFC1	4p14	0.39863595	0.00022782	0.0124736
CHRM3	1q43	0.39855465	0.00022855	0.01247389
CGNL1	15q21.3	0.39780939	0.00023539	0.01280651
MON1B	16q23.1	0.39756098	0.00023772	0.01288581
SCRN3	2q31.1	0.39747064	0.00023857	0.01288581
DCAF11	14q12	0.39733514	0.00023985	0.01288581
PTPN1	20q13.13	0.39560973	0.00025671	0.01370429
VEZF1	17q22	0.39539295	0.0002589	0.01377836
CEP152	15q21.1	0.39521229	0.00026075	0.01383327
ZNF70	22q11.23	0.3946477	0.00026658	0.01409895
BMPR2	2q33.1-q33.2	0.39400629	0.00027335	0.01438844
HIPK1	1p13.2	0.39397019	0.00027374	0.01438844
ADAL	15q15.3	0.39339653	0.00027994	0.01463196
USP4	3p21.31	0.39320235	0.00028206	0.01467161
ITGA2	5q11.2	0.39315718	0.00028256	0.01467161
PPP2R5E	14q23.2	0.39297651	0.00028456	0.01472349
ZNF264	19q13.43	0.39291098	0.00028528	0.01472349
FAM120A	9q22.31	0.39272809	0.00028732	0.01478402
KBTBD6	13q14.11	0.39241192	0.00029088	0.014922
DCAF16	4p15.31	0.391757	0.00029837	0.01521502
TGFBRAP1	2q12.1-q12.2	0.39074074	0.00031035	0.01569912
HIST1H1D	6p22.2	0.39071816	0.00031062	0.01569912
CNNM3	2q11.2	0.39048323	0.00031345	0.0157956

LATS2	13q12.11	0.39021223	0.00031675	0.01589918
RAPGEF4	2q31.1	0.39008582	0.0003183	0.01589918
TAF8	6p21.1	0.39008582	0.0003183	0.01589918
KIR3DL3	19q13.42	0.38992773	0.00032025	0.01594981
THSD4	15q23	0.38963415	0.00032389	0.01608448
HMGXB4	22q12.3	0.38882114	0.00033419	0.01649979
CBX1	17q21.32	0.38846199	0.00033883	0.0165866
CAVIN2	2q32.3	0.38810066	0.00034356	0.01676872
CDC5L	6p21.1	0.38649503	0.00036532	0.01772961
SYNJ2	6q25.3	0.38604336	0.00037167	0.01798648
PTTG1IP	21q22.3	0.38584011	0.00037456	0.01806143
ZNF292	6q14.3	0.38578583	0.00037533	0.01806143
TTC9	14q24.2	0.38534327	0.00038171	0.01817903
SEL1L	14q31.1	0.38533415	0.00038184	0.01817903
ZNF518A	10q24.1	0.38532286	0.000382	0.01817903
PPM1A	14q23.1	0.38532069	0.00038203	0.01817903
ATP5F1E	20q13.32	0.38486902	0.00038864	0.01838758
TSPAN18	11p11.2	0.38461148	0.00039246	0.01846944
CSNK1A1L	13q13.3	0.38446251	0.00039468	0.01848811
PNISR	6q16.2	0.38423668	0.00039808	0.01858015
BCAS3	17q23.2	0.38383017	0.00040425	0.01881689
MDM4	1q32.1	0.38369467	0.00040633	0.01886219
IKZF2	2q34	0.38319783	0.00041403	0.0190617
CAP1	1p34.2	0.38313224	0.00041505	0.0190617
XBP1	22q12.1|22q	0.38313008	0.00041509	0.0190617
SLC9A2	2q12.1	0.38263324	0.00042294	0.01931844
KIF16B	20p12.1	0.38247516	0.00042547	0.01934536
FAM193A	4p16.3	0.38245473	0.0004258	0.01934536
KANK2	19p13.2	0.38236224	0.00042728	0.01936138
CEP85L	6q22.31	0.38148148	0.00044167	0.01981695
TPCN1	12q24.13	0.3813933	0.00044313	0.01981695
NBPF3	1p36.12	0.38116531	0.00044694	0.01993505
MPHOSPH8	13q12.11	0.38102981	0.00044922	0.01998444
PPP2R5A	1q32.3	0.38069106	0.00045496	0.02018721
ITPKB	1q42.12	0.38026197	0.00046233	0.02040807
RAX2	19p13.3	0.3800813	0.00046546	0.02044078
TYW1B	7q11.22-q11	0.3800813	0.00046546	0.02044078
PDCD7	15q22.31	0.37985761	0.00046937	0.02053292
PXDC1	6p25.2	0.37982373	0.00046996	0.02053292
TCF4	18q21.2	0.3796748	0.00047258	0.0205597
TMEM150C	4q21.22	0.37965221	0.00047298	0.0205597
MAP1LC3B	16q24.2	0.37922313	0.00048062	0.02069842
TMEM87A	15q15.1	0.37920054	0.00048102	0.02069842
CHD6	20q12	0.37908762	0.00048305	0.0207335
MPPED2	11p14.1	0.37863595	0.00049124	0.02101508
TEAD1	11p15.3	0.37859079	0.00049207	0.02101508
SLC38A7	16q21	0.3783445	0.0004966	0.02115558
CSRP1	1q32.1	0.3781617	0.00049998	0.02124683

PRICKLE1	12q12	0.37764441	0.00050968	0.02154278
KPNA3	13q14.2	0.37764228	0.00050972	0.02154278
DST	6p12.1	0.37757453	0.000511	0.02154278
NEIL1	15q24.2	0.37752233	0.00051199	0.02154278
EPHX1	1q42.12	0.37741644	0.00051401	0.02157439
KMT2E	7q22.3	0.37701207	0.00052176	0.02179609
NPAS3	14q13.1	0.37696477	0.00052268	0.02179609
LUC7L	16p13.3	0.37662602	0.00052927	0.02194549
N4BP2	4p14	0.3765131	0.00053149	0.02198394
PITPNB	22q12.1	0.37640018	0.00053371	0.02202255
ARPC5	1q25.3	0.37588076	0.00054404	0.02228745
EID1	15q21.1	0.37518067	0.00055826	0.02265261
PATJ	1p31.3	0.37506775	0.00056058	0.02269303
CBX5	12q13.13	0.37454833	0.00057139	0.02307586
ANKRD17	4q13.3	0.37445799	0.00057329	0.02309795
IPMK	10q21.1	0.37434508	0.00057567	0.02310416
ACTR2	2p14	0.37432249	0.00057615	0.02310416
TFAP2A	6p24.3	0.37420958	0.00057854	0.02312641
ATP6V1A	3q13.31	0.37416863	0.00057941	0.02312641
COL4A3BP	5q13.3	0.3737579	0.0005882	0.0234226
OTX1	2p15	0.37301265	0.00060447	0.02395854
SHROOM3	4q21.1	0.37267389	0.000612	0.02420075
NAPA	19q13.32-q1	0.37260614	0.00061351	0.02420466
AKAP1	17q22	0.37247064	0.00061655	0.02423278
FUT6	19p13.3	0.37244806	0.00061706	0.02423278
ZNF44	19p13.2	0.37199639	0.00062731	0.02452243
TNRC6B	22q13.1	0.3718383	0.00063093	0.0245921
SPEN	1p36.21-p36	0.37179313	0.00063197	0.0245921
LRIG3	12q14.1	0.37127371	0.00064403	0.0249476
SNTB2	16q22.1	0.37127371	0.00064403	0.0249476
ANKRD13A	12q24.11	0.37112901	0.00064742	0.0250224
KIF3A	5q31.1	0.37065476	0.00065867	0.02534232
RAPGEF3	12q13.11	0.37057362	0.00066061	0.0253599
ZNF281	1q32.1	0.3704607	0.00066332	0.02540687
GOLGA2P7	15q25.2	0.36942186	0.00068874	0.02620419
RBM43	2q23.3	0.36929974	0.00069178	0.02626158
ACSL3	2q36.1	0.36917344	0.00069494	0.02632314
AGGF1	5q13.3	0.36906052	0.00069778	0.02635695
NR2F1	5q15	0.36894761	0.00070063	0.02635695
TPR	1q31.1	0.36889323	0.00070201	0.02635695
ADSS	1q44	0.36874435	0.00070579	0.02644075
JMJD1C	10q21.3	0.3684056	0.00071446	0.02664865
CEP350	1q25.2	0.36824959	0.00071848	0.0266914
ZCCHC17	1p35.2	0.36797651	0.00072558	0.02674278
NOTCH2NLA	1q21.1	0.36793135	0.00072676	0.02674278
DDX24	14q32.12	0.36788618	0.00072794	0.02674278
PYGB	20p11.21	0.36768293	0.00073328	0.02688109
TMBIM4	12q14.3	0.36752484	0.00073745	0.02697631

TLN2	15q22.2	0.36623758	0.00077228	0.02789186
HERPUD1	16q13	0.36593477	0.00078069	0.02807676
CMAHP	6p22.3	0.36578591	0.00078485	0.02812667
ACAP2	3q29	0.36576745	0.00078537	0.02812667
LZTS2	10q24.31	0.36519874	0.00080147	0.02858347
CCNI	4q21.1	0.36485998	0.0008112	0.02879333
PRRC2C	1q24.3	0.36479223	0.00081316	0.02879333
MMUT	6p12.3	0.36473783	0.00081474	0.02879333
SERHL2	22q13.2	0.36447606	0.00082236	0.02883401
ARRDC3	5q14.3	0.36445348	0.00082302	0.02883401
KCNE4	2q36.1	0.36443089	0.00082368	0.02883401
DCAF5	14q24.1	0.36379855	0.0008424	0.02924938
MALAT1	11q13.1	0.36347315	0.00085218	0.02946919
IL6ST	5q11.2	0.36289521	0.00086981	0.03001794
RHOU	1q42.13	0.36239837	0.00088522	0.03047564
MTUS1	8p22	0.36235321	0.00088664	0.03047564
BCL10	1p22.3	0.36187895	0.0009016	0.03086595
ADD1	4p16.3	0.36187895	0.0009016	0.03086595
FGF7	15q21.2	0.36129178	0.00092046	0.03138552
PABPC3	13q12.13	0.36129178	0.00092046	0.03138552
CASQ2	1p13.1	0.36084011	0.0009352	0.03182473
FKBP2	11q13.1	0.35941734	0.00098306	0.03312119
MDM2	12q15	0.35905601	0.00099556	0.03341284
TGFBR3	1p22.1	0.35885276	0.00100266	0.03358502
ELF1	13q14.11	0.35869467	0.00100821	0.03370487
UBN1	16p13.3	0.35842367	0.00101779	0.03389251
RNF11	1p32.3	0.3581075	0.00102907	0.03406898
BICRAL	6p21.1	0.35774616	0.0010421	0.03436724
TTLL12	22q13.2	0.35763324	0.00104621	0.03436986
FAM98A	2p22.3	0.35754291	0.0010495	0.03437294
RSBN1L	7q11.23	0.35724005	0.00106061	0.0345112
PDE4D	5q11.2-q12.1	0.35707067	0.00106687	0.03464888
KNL1	15q15.1	0.35650608	0.00108798	0.03520059
PCDHB12	5q31.3	0.35612014	0.00110262	0.03550066
THUMPD1	16p12.3	0.35609756	0.00110349	0.03550066
ASPH	8q12.3	0.35600723	0.00110694	0.03552459
MYLIP	6p22.3	0.35591689	0.00111041	0.03552459
FHL3	1p34.3	0.35578139	0.00111563	0.03552459
CDH1	16q22.1	0.35575881	0.0011165	0.03552459
DGKA	12q13.2	0.35569106	0.00111912	0.03552459
RNF115	1q21.1	0.35564589	0.00112087	0.03552459
TCF7L1	2p11.2	0.35517164	0.00113938	0.03604457
PPARA	22q13.31	0.35487805	0.00115098	0.03627721
RALBP1	18p11.22	0.35458446	0.00116269	0.03656835
RBM42	19q13.12	0.35433604	0.00117268	0.03662326
EPB41L4A-D	5q22.2	0.35383921	0.0011929	0.03701286
FAM177A1	14q13.2	0.35381662	0.00119382	0.03701286
MEF2C	5q14.3	0.35365854	0.00120033	0.03704167

ABHD2	15q26.1	0.35363595	0.00120126	0.03704167
PDLIM3	4q35.1	0.35334237	0.00121343	0.03728235
WDR82	3p21.2	0.35318428	0.00122003	0.03741782
ZBTB10	8q21.13	0.3530262	0.00122666	0.03751564
KIF27	9q21.32	0.35300361	0.00122761	0.03751564
HIST1H4D	6p22.2	0.35275519	0.0012381	0.03770147
ATP10D	4p12	0.35255393	0.00124667	0.03777487
ITSN2	2p23.3	0.35255194	0.00124675	0.03777487
TCL1B	14q32.13	0.35221319	0.00126128	0.03813542
SEPHS2	16p11.2	0.35207769	0.00126714	0.03824479
SERHL	22q13.2	0.35185185	0.00127695	0.03847305
TGOLN2	2p11.2	0.35163929	0.00128626	0.03868504
NFIA	1p31.3	0.3515131	0.00129181	0.03878371
NCALD	8q22.3	0.35123279	0.00130421	0.03908751
ZNF638	2p13.3-p13.2	0.35111987	0.00130924	0.0391695
GLYR1	16p13.3	0.35101824	0.00131378	0.03923663
SP100	2q37.1	0.35051942	0.00133628	0.03975856
RFTN1	3p24.3	0.350271	0.00134761	0.03985965
TEF	22q13.2	0.35024842	0.00134864	0.03985965
SLC35E3	12q15	0.35024842	0.00134864	0.03985965
DEPTOR	8q24.12	0.34961608	0.0013779	0.0404445
CENPT	16q22.1	0.34952575	0.00138213	0.04046058
NPHP3	3q22.1	0.34950316	0.00138319	0.04046058
CSNK1G1	15q22.31	0.34909666	0.00140236	0.04088155
ALDH1A3	15q26.3	0.34902891	0.00140558	0.04089849
CDH11	16q21	0.34889341	0.00141204	0.04095403
GGA1	22q13.1	0.34878246	0.00141735	0.04103832
CEP295	11q21	0.34859982	0.00142613	0.04120917
PNPLA8	7q31.1	0.34846432	0.00143267	0.04120917
RHOJ	14q23.2	0.34841915	0.00143486	0.04120917
NOL4L	20q11.21	0.34840983	0.00143531	0.04120917
MBD5	2q23.1	0.3483514	0.00143815	0.04122128
RSRC1	3q25.32	0.34810298	0.00145026	0.04149876
DAD1	14q11.2	0.34765327	0.00147242	0.04195681
PPME1	11q13.4	0.34762873	0.00147363	0.04195681
DCAF1	3p21.2	0.34717706	0.00149621	0.0424655
DNAJC17	15q15.1	0.34712256	0.00149896	0.0424655
MRVI1	11p15.4	0.34699639	0.00150533	0.04257544
MARVELD3	16q22.2	0.34654472	0.00152835	0.04294204
MEIS2	15q14	0.34622855	0.00154465	0.04325773
PPARGC1B	5q32	0.34593496	0.00155992	0.04357053
PARD3B	2q33.3	0.34586721	0.00156347	0.04357053
LRRC37A2	17q21.31	0.34566591	0.00157404	0.04379378
SNX19	11q24.3-q25	0.3454607	0.00158489	0.04395239
ENPP1	6q23.2	0.3451897	0.00159932	0.04428061
MAP3K2	2q14.3	0.3450542	0.00160658	0.04432861
PKD2	4q22.1	0.34503357	0.00160768	0.04432861
ADRB1	10q25.3	0.34500903	0.001609	0.04432861

ZNF791	19p13.13	0.34479644	0.00162046	0.04450546
HM13	20q11.21	0.34473803	0.00162363	0.04452073
PFDN1	5q31.3	0.3446477	0.00162853	0.04458357
CHST2	3q24	0.34444639	0.0016395	0.04481212
NDE1	16p13.11	0.3442996	0.00164754	0.04496001
ZYG11B	1p32.3	0.34408505	0.00165936	0.04520918
SIDT1	3q13.2	0.34397019	0.00166572	0.04523924
GCC2	2q12.3	0.34374629	0.00167818	0.04550523
COX20	1q44	0.34356369	0.0016884	0.04570988
FAM3D	3p14.2	0.34324752	0.00170623	0.04600858
MYH14	19q13.33	0.34322493	0.00170751	0.04600858
CTDSP1	2q35	0.34310266	0.00171446	0.04612301
PPTC7	12q24.11	0.34304426	0.00171779	0.04613984
GTPBP8	3q13.2	0.34243451	0.00175287	0.04661275
UBE2Z	17q21.32	0.34232159	0.00175944	0.04661275
TRIB1	8q24.13	0.34214092	0.00176999	0.04661275
KIF21A	12q12	0.34209575	0.00177264	0.04661275
ITPRID2	2q31.3	0.34193767	0.00178193	0.04668833
CRIP2	14q32.33	0.34112659	0.00183032	0.04712737
SIPA1L2	1q42.2	0.34112659	0.00183032	0.04712737
CBL	11q23.3	0.34110208	0.0018318	0.04712737
PCDHAC2	5q31.3	0.34103818	0.00183566	0.04715578
CHRNA3	15q25.1	0.34089883	0.00184411	0.04726604
CREBBP	16p13.3	0.34087624	0.00184549	0.04726604
TNFRSF13C	22q13.2	0.34074074	0.00185374	0.04740647
BECN1	17q21.31	0.34059587	0.00186261	0.04756201
SUN1	7p22.3	0.34044715	0.00187175	0.04772409
ZNF345	19q13.12	0.33995032	0.00190259	0.04843795
MSRB3	12q14.3	0.33945348	0.00193388	0.0489428
WDFY1	2q36.1	0.33911664	0.00195535	0.04928559
ZNF564	19p13.2	0.33866305	0.00198461	0.04963851

TCGA TNBC

Correlated G	Cytoband	Spearman's (	p-Value	q-Value
TNRC6B	22q13.1	0.78877026	1.38E-18	2.76E-14
DPP8	15q22.31	0.76201826	9.24E-17	9.20E-13
UHMK1	1q23.3	0.75139583	4.22E-16	2.80E-12
PRR14L	22q12.2	0.74850078	6.29E-16	3.02E-12
ZDHHC20	13q12.11	0.74715121	7.57E-16	3.02E-12
NBEAL1	2q33.2	0.73655054	3.11E-15	9.45E-12
GTF2A1	14q31.1	0.73604989	3.32E-15	9.45E-12
TAOK1	17q11.2	0.733982	4.34E-15	1.08E-11
NCOA2	8q13.3	0.7329154	4.97E-15	1.10E-11
PIKFYVE	2q34	0.73104341	6.31E-15	1.26E-11
ZKSCAN1	7q22.1	0.72797423	9.29E-15	1.68E-11
LMBRD2	5p13.2	0.72449146	1.43E-14	2.38E-11
ASXL2	2p23.3	0.72129167	2.12E-14	3.21E-11
REST	4q12	0.72079102	2.25E-14	3.21E-11
LNPEP	5q15	0.71645933	3.79E-14	5.04E-11
MYSM1	1p32.1	0.71539274	4.30E-14	5.36E-11
ANKRD36BP1	1q24.2	0.71480502	4.62E-14	5.41E-11
MYO9A	15q23	0.71334661	5.48E-14	6.06E-11
MACF1	1p34.3	0.71234532	6.16E-14	6.46E-11
MINDY2	15q21.3-q22	0.71025566	7.86E-14	7.83E-11
REL	2p16.1	0.70958087	8.50E-14	8.06E-11
USF3	3q13.2	0.70803539	1.02E-13	9.20E-11
SMG1	16p12.3	0.70157051	2.12E-13	1.76E-10
USP12	13q12.13	0.69802244	3.14E-13	2.50E-10
LCOR	10q24.1	0.6965205	3.70E-13	2.83E-10
CDKL5	Xp22.13	0.69434377	4.69E-13	3.34E-10
BIRC6	2p22.3	0.69373429	5.01E-13	3.44E-10
RAPGEF6	5q31.1	0.69340778	5.19E-13	3.45E-10
ARFGEF2	20q13.13	0.69142695	6.43E-13	4.00E-10
MAN1A2	1p12	0.69027329	7.27E-13	4.39E-10
HIPK3	11p13	0.68999031	7.50E-13	4.39E-10
APC	5q22.2	0.68676876	1.06E-12	6.00E-10
VPS13B	8q22.2	0.68605043	1.14E-12	6.30E-10
LATS1	6q25.1	0.68089159	1.95E-12	1.05E-09
MAP3K2	2q14.3	0.67869309	2.44E-12	1.23E-09
FAM168A	11q13.4	0.67856249	2.47E-12	1.23E-09
IL6ST	5q11.2	0.67640753	3.08E-12	1.49E-09
ZNF641	12q13.11	0.67192347	4.82E-12	2.18E-09
HIPK1	1p13.2	0.67168403	4.94E-12	2.19E-09
KIAA0754	1p34.3	0.6697032	6.01E-12	2.55E-09
ASH1L	1q22	0.66909372	6.38E-12	2.65E-09
PLXNC1	12q22	0.66780945	7.23E-12	2.88E-09
KLHL11	17q21.2	0.6672435	7.65E-12	2.95E-09
PARD3B	2q33.3	0.66702583	7.81E-12	2.95E-09
DDI2	1p36.21	0.66656871	8.17E-12	3.01E-09
NOTCH2	1p12	0.66487087	9.63E-12	3.42E-09

HEATR5A	14q12	0.6638478	1.06E-11	3.69E-09
UBR5	8q22.3	0.66373897	1.07E-11	3.69E-09
CCNT1	12q13.11-q1	0.66295534	1.16E-11	3.91E-09
NIPBL	5p13.2	0.6627159	1.19E-11	3.93E-09
SKIL	3q26.2	0.66234586	1.23E-11	4.01E-09
ITGAV	2q32.1	0.66117043	1.37E-11	4.31E-09
SERINC5	5q14.1	0.66108336	1.39E-11	4.31E-09
SPATA13	13q12.12	0.66049564	1.47E-11	4.48E-09
STRN	2p22.2	0.66036504	1.48E-11	4.48E-09
PEAK1	15q24.3	0.65936374	1.63E-11	4.79E-09
BDP1	5q13.2	0.65923314	1.65E-11	4.79E-09
SLFN5	17q12	0.6591896	1.66E-11	4.79E-09
RIF1	2q23.3	0.65853659	1.76E-11	4.99E-09
RAD54L2	3p21.2	0.65844952	1.78E-11	4.99E-09
RASA2	3q23	0.65701288	2.04E-11	5.63E-09
INO80D	2q33.3	0.65585921	2.27E-11	6.19E-09
TCF20	22q13.2|22q	0.65487968	2.49E-11	6.54E-09
ZBTB37	1q25.1	0.65483615	2.50E-11	6.54E-09
DENND1B	1q31.3	0.65424843	2.64E-11	6.73E-09
TTBK2	15q15.2	0.65363895	2.79E-11	7.04E-09
HERC1	15q22.31	0.65298593	2.96E-11	7.38E-09
BMPR2	2q33.1-q33.2	0.65274649	3.03E-11	7.45E-09
MGAT5	2q21.2-q21.3	0.64891544	4.31E-11	1.05E-08
ADAT1	16q23.1	0.64863247	4.42E-11	1.06E-08
EFCAB14	1p33	0.64776178	4.78E-11	1.13E-08
ZNF81	Xp11.23	0.64739173	4.95E-11	1.16E-08
BICRAL	6p21.1	0.64617277	5.52E-11	1.22E-08
HECTD1	14q12	0.64617277	5.52E-11	1.22E-08
NR2C2	3p25.1	0.64517147	6.05E-11	1.30E-08
BTBD8	1p22.1	0.6451641	6.05E-11	1.30E-08
KBTBD7	13q14.11	0.64480143	6.25E-11	1.32E-08
KMT2E	7q22.3	0.64460552	6.36E-11	1.33E-08
SMG1P1	16p12.2	0.64223289	7.86E-11	1.60E-08
LOC1001909	16p12.2	0.64190638	8.09E-11	1.63E-08
KMT2A	11q23.3	0.64160164	8.31E-11	1.66E-08
BTAF1	10q23.32	0.63946844	1.00E-10	1.92E-08
EEA1	12q22	0.6390331	1.04E-10	1.93E-08
ERN1	17q23.3	0.6380318	1.14E-10	2.06E-08
PHC3	3q26.2	0.63794473	1.15E-10	2.06E-08
BARD1	2q35	0.63729171	1.22E-10	2.14E-08
NHSL2	Xq13.1	0.63711758	1.23E-10	2.16E-08
IDE	10q23.33	0.63592038	1.37E-10	2.37E-08
ELK4	1q32.1	0.6344402	1.56E-10	2.65E-08
SLX4IP	20p12.2	0.63398309	1.62E-10	2.73E-08
ZBTB20	3q13.31	0.63282942	1.79E-10	2.94E-08
ZFP91	11q12.1	0.63213287	1.90E-10	3.05E-08
TCP11L2	12q23.3	0.63161045	1.99E-10	3.16E-08
ZNF829	19q13.12	0.62878071	2.53E-10	3.90E-08

MIEF1	22q13.1	0.62878071	2.53E-10	3.90E-08
DMXL1	5q23.1	0.62736583	2.85E-10	4.30E-08
FAM126B	2q33.1	0.62357832	3.91E-10	5.64E-08
SLC30A4	15q21.1|15q	0.62214168	4.40E-10	6.08E-08
PPTC7	12q24.11	0.62207638	4.43E-10	6.08E-08
ZNF678	1q42.13	0.62151043	4.64E-10	6.24E-08
HACD2	3q21.1	0.62048737	5.05E-10	6.70E-08
SBNO1	12q24.31	0.62022616	5.16E-10	6.80E-08
CEP85L	6q22.31	0.61998672	5.26E-10	6.85E-08
LEPROT	1p31.3	0.61935547	5.54E-10	7.16E-08
POMK	8p11.21	0.61885482	5.77E-10	7.38E-08
ZNF619	3p22.1	0.61846301	5.96E-10	7.56E-08
CHD2	15q26.1	0.61815827	6.11E-10	7.68E-08
ROCK2	2p25.1	0.61774469	6.32E-10	7.86E-08
AKAP11	13q14.11	0.61696107	6.73E-10	8.33E-08
ZKSCAN8	6p22.1	0.6167434	6.85E-10	8.43E-08
CCDC186	10q25.3	0.61622098	7.15E-10	8.68E-08
KLF7	2q33.3	0.6158727	7.35E-10	8.88E-08
AFF4	5q31.1	0.61545913	7.60E-10	9.12E-08
MAML2	11q21	0.61504555	7.86E-10	9.38E-08
FMN1	15q13.3	0.61458844	8.16E-10	9.61E-08
PDPR	16q22.1	0.61434899	8.32E-10	9.69E-08
RNF111	15q22.1-q22	0.61424016	8.39E-10	9.71E-08
HELZ	17q24.2	0.61358714	8.84E-10	1.01E-07
ABHD2	15q26.1	0.61249878	9.65E-10	1.09E-07
ATM	11q22.3	0.61232464	9.78E-10	1.09E-07
N4BP2	4p14	0.61073563	1.11E-09	1.23E-07
BROX	1q41	0.61040912	1.14E-09	1.25E-07
ROCK1	18q11.1	0.6098214	1.19E-09	1.30E-07
XPO4	13q12.11	0.60956019	1.22E-09	1.31E-07
SPEN	1p36.21-p36	0.60945136	1.23E-09	1.32E-07
PAFAH1B2	11q23.3	0.60829769	1.35E-09	1.40E-07
PEX26	22q11.21	0.60803648	1.37E-09	1.41E-07
OSBPL8	12q21.2	0.60786234	1.39E-09	1.41E-07
STK4	20q13.12	0.60781881	1.40E-09	1.41E-07
PPP4R2	3p13	0.60696988	1.49E-09	1.47E-07
SEC24A	5q31.1	0.6064257	1.56E-09	1.53E-07
FER	5q21.3	0.60590329	1.62E-09	1.59E-07
LMTK2	7q21.3	0.60511967	1.73E-09	1.68E-07
CREB1	2q33.3	0.6050326	1.74E-09	1.68E-07
CHD9	16q12.2	0.60474962	1.78E-09	1.69E-07
GOLIM4	3q26.2	0.60468432	1.79E-09	1.69E-07
AKAP9	7q21.2	0.60466255	1.79E-09	1.69E-07
ELK3	12q23.1	0.60457548	1.80E-09	1.69E-07
PIK3CA	3q26.32	0.60418367	1.86E-09	1.72E-07
HIVEP1	6p24.1	0.60379186	1.92E-09	1.77E-07
MARF1	16p13.11	0.60287764	2.06E-09	1.87E-07
ZNF791	19p13.13	0.6016369	2.26E-09	2.02E-07

ZNF699	19p13.2	0.60111449	2.36E-09	2.08E-07
PREX2	8q13.2	0.60110687	2.36E-09	2.08E-07
ZFR	5p13.3	0.600505	2.47E-09	2.17E-07
MOSPD2	Xp22.2	0.60024379	2.52E-09	2.19E-07
PRKG1	10q11.23-q2	0.60022203	2.52E-09	2.19E-07
NPAT	11q22.3	0.59954724	2.66E-09	2.27E-07
NHLRC2	10q25.3	0.59848064	2.88E-09	2.43E-07
ADAM10	15q21.3	0.59821944	2.94E-09	2.47E-07
IQGAP1	15q26.1	0.59789293	3.01E-09	2.52E-07
MTMR12	5p13.3	0.59771879	3.05E-09	2.54E-07
PHIP	6q14.1	0.59760995	3.08E-09	2.55E-07
LPP	3q27.3-q28	0.59743581	3.12E-09	2.57E-07
MED13	17q23.2	0.5971093	3.20E-09	2.62E-07
SPAG9	17q21.33	0.59663042	3.32E-09	2.67E-07
TRIO	5p15.2	0.59493258	3.77E-09	2.98E-07
UBXN7	3q29	0.59480197	3.81E-09	2.99E-07
ATRX	Xq21.1	0.5947802	3.81E-09	2.99E-07
RASSF3	12q14.2	0.59436663	3.93E-09	3.06E-07
KIAA1109	4q27	0.59388775	4.08E-09	3.16E-07
FNIP2	4q32.1	0.59377891	4.11E-09	3.17E-07
ERBIN	5q12.3	0.593583	4.17E-09	3.21E-07
RC3H2	9q33.2	0.59327826	4.27E-09	3.25E-07
SOX5	12p12.1	0.59310412	4.32E-09	3.27E-07
CPLANE1	5p13.2	0.59262524	4.48E-09	3.37E-07
RALGAPA2	20p11.23	0.59181985	4.76E-09	3.56E-07
FRYL	4p11	0.591058	5.03E-09	3.70E-07
ZNF281	1q32.1	0.59073149	5.16E-09	3.78E-07
ETV3	1q23.1	0.59027438	5.34E-09	3.89E-07
TRIP11	14q32.12	0.59001317	5.44E-09	3.94E-07
MAPK1	22q11.22	0.58977373	5.54E-09	4.00E-07
RPS6KA3	Xp22.12	0.58933838	5.72E-09	4.09E-07
SMG1P3	16p12.2	0.58912071	5.81E-09	4.12E-07
MTMR3	22q12.2	0.58888127	5.91E-09	4.18E-07
ATG2B	14q32.2	0.5887289	5.98E-09	4.21E-07
SECISBP2L	15q21.1	0.58783644	6.39E-09	4.43E-07
HECTD4	12q24.13	0.58777114	6.42E-09	4.44E-07
GIGYF2	2q37.1	0.58637803	7.11E-09	4.81E-07
MGA	15q15.1	0.58568148	7.48E-09	5.03E-07
BPTF	17q24.2	0.58557264	7.54E-09	5.05E-07
MED13L	12q24.21	0.58552911	7.56E-09	5.05E-07
KIF13A	6p22.3	0.5852679	7.71E-09	5.13E-07
PWAR5	15q11.2	0.58500588	7.85E-09	5.22E-07
SLC16A7	12q14.1	0.58474549	8.00E-09	5.30E-07
MDN1	6q15	0.58452781	8.13E-09	5.36E-07
SYNE2	14q23.2	0.58446251	8.17E-09	5.36E-07
ZNF585B	19q13.12	0.58444074	8.18E-09	5.36E-07
XIAP	Xq25	0.58428837	8.28E-09	5.40E-07
PBRM1	3p21.1	0.584136	8.37E-09	5.45E-07

SON	21q22.11	0.58309117	9.02E-09	5.82E-07
SSH1	12q24.11	0.5830041	9.08E-09	5.84E-07
XRN1	3q23	0.58265583	9.31E-09	5.95E-07
HMGXB4	22q12.3	0.58256876	9.37E-09	5.95E-07
EYA3	1p35.3	0.58254699	9.39E-09	5.95E-07
GPATCH2L	14q24.3	0.58222048	9.61E-09	6.06E-07
ZBTB38	3q23	0.58211164	9.69E-09	6.09E-07
SLC30A6	2p22.3	0.58193751	9.81E-09	6.12E-07
BICC1	10q21.1	0.5818722	9.85E-09	6.13E-07
TMF1	3p14.1	0.58165453	1.00E-08	6.21E-07
MED1	17q12	0.58154569	1.01E-08	6.24E-07
ZBTB11	3q12.3	0.58124095	1.03E-08	6.30E-07
FZD6	8q22.3	0.58113212	1.04E-08	6.32E-07
SCAF11	12q12	0.58106681	1.04E-08	6.32E-07
CLIP1	12q24.31	0.58102328	1.05E-08	6.32E-07
ERCC6L2	9q22.32	0.5805444	1.08E-08	6.48E-07
NCOA3	20q13.12	0.58028319	1.10E-08	6.57E-07
UHRF1BP1L	12q23.1	0.57884655	1.22E-08	7.21E-07
RNF169	11q13.4	0.57843297	1.26E-08	7.38E-07
DENND4A	15q22.31	0.5782806	1.27E-08	7.44E-07
MPZL3	11q23.3	0.57812823	1.29E-08	7.48E-07
CLDN20	6q25.3	0.57773657	1.32E-08	7.67E-07
CREBRF	5q35.1	0.57758405	1.34E-08	7.72E-07
NBPF10	1q21.1	0.57756228	1.34E-08	7.72E-07
PIK3C2A	11p15.1	0.5771487	1.38E-08	7.90E-07
MBOAT1	6p22.3	0.57680043	1.42E-08	8.03E-07
ZBTB41	1q31.3	0.57680043	1.42E-08	8.03E-07
NUP50	22q13.31	0.57647392	1.45E-08	8.17E-07
AKAP13	15q25.3	0.57640862	1.46E-08	8.19E-07
KMT2D	12q13.12	0.57592974	1.51E-08	8.44E-07
EXOC6B	2p13.2	0.57473253	1.64E-08	9.01E-07
LYSMD3	5q14.3	0.57440603	1.68E-08	9.17E-07
SERINC3	20q13.12	0.57418835	1.70E-08	9.28E-07
OSBP	11q12.1	0.57375301	1.75E-08	9.55E-07
MYCBP2	13q22.3	0.57344826	1.79E-08	9.67E-07
BMP2K	4q21.21	0.57316529	1.83E-08	9.84E-07
UGGT1	2q14.3	0.57255581	1.91E-08	1.0211E-06
USP37	2q35	0.57218576	1.96E-08	1.0422E-06
REV3L	6q21	0.57144567	2.06E-08	1.0857E-06
SESTD1	2q31.2	0.57138037	2.07E-08	1.0865E-06
DDR2	1q23.3	0.57135861	2.07E-08	1.0865E-06
RICTOR	5p13.1	0.57124977	2.09E-08	1.0907E-06
ITSN2	2p23.3	0.571228	2.09E-08	1.0907E-06
FKSG29	13q32.3	0.57068492	2.17E-08	1.118E-06
MON2	12q14.1	0.57040084	2.22E-08	1.1372E-06
UTP14C	13q14.3	0.57031378	2.23E-08	1.1412E-06
CACNA2D1	7q21.11	0.56961722	2.34E-08	1.1853E-06
RTL6	22q13.31	0.56955192	2.35E-08	1.1877E-06

MRTFB	16p13.12	0.56944308	2.37E-08	1.1936E-06
DPY19L3	19q13.11	0.56920364	2.41E-08	1.2104E-06
XPNPEP3	22q13.2	0.5689642	2.45E-08	1.2244E-06
TMPPE	3p22.3	0.56885537	2.46E-08	1.2305E-06
GPATCH8	17q21.31	0.56842002	2.54E-08	1.2648E-06
SLC4A7	3p24.1	0.56813705	2.59E-08	1.2832E-06
DGKH	13q14.11	0.56785407	2.64E-08	1.3052E-06
VPS13C	15q22.2	0.567767	2.66E-08	1.3079E-06
PCDHGA12	5q31.3	0.56772347	2.66E-08	1.3079E-06
ANKRD44	2q33.1	0.56767993	2.67E-08	1.3079E-06
NFAT5	16q22.1	0.56715752	2.77E-08	1.3457E-06
BOD1L1	4p15.33	0.56709222	2.78E-08	1.3485E-06
GPRIN3	4q22.1	0.5666351	2.87E-08	1.3734E-06
ZFX	Xp22.11	0.5663739	2.92E-08	1.3927E-06
USP34	2p15	0.56619976	2.96E-08	1.406E-06
LOC284441	19p12	0.56589502	3.02E-08	1.4321E-06
EDEM3	1q25.3	0.56580795	3.04E-08	1.4373E-06
TOGARAM1	14q21.2	0.56576441	3.05E-08	1.4381E-06
CLOCK	4q12	0.56569911	3.06E-08	1.4399E-06
BTBD7	14q32.12	0.56567734	3.07E-08	1.4399E-06
CEP97	3q12.3	0.5654379	3.12E-08	1.4601E-06
ATE1	10q26.13	0.56539437	3.12E-08	1.461E-06
ICE1	5p15.32	0.56519846	3.17E-08	1.4772E-06
CDK12	17q12	0.56506786	3.20E-08	1.4869E-06
ARID2	12q12	0.56500256	3.21E-08	1.49E-06
ATXN1L	16q22.2	0.564306	3.36E-08	1.5514E-06
BCOR	Xp11.4	0.56391419	3.46E-08	1.5859E-06
SLC35A3	1p21.2	0.56339178	3.58E-08	1.6392E-06
HEG1	3q21.2	0.5629129	3.70E-08	1.6854E-06
FUT11	10q22.2	0.56223811	3.87E-08	1.7559E-06
RNF168	3q29	0.56210751	3.90E-08	1.7674E-06
IBTK	6q14.1	0.56206397	3.92E-08	1.7686E-06
WDFY3	4q21.23	0.561781	3.99E-08	1.7945E-06
ZNF770	15q14	0.56082324	4.26E-08	1.9009E-06
FRK	6q22.1	0.56069264	4.29E-08	1.9049E-06
NKTR	3p22.1	0.56060557	4.32E-08	1.9118E-06
PTAR1	9q21.12	0.55980018	4.56E-08	1.9999E-06
FNDC3B	3q26.31	0.55966957	4.60E-08	2.013E-06
ADNP	20q13.13	0.55888595	4.85E-08	2.1073E-06
ZNF292	6q14.3	0.55877711	4.88E-08	2.1134E-06
LIMS1	2q12.3	0.55873358	4.90E-08	2.1149E-06
TOR1AIP2	1q25.2	0.5584506	4.99E-08	2.1475E-06
MAN2A1	5q21.3	0.55751461	5.31E-08	2.2449E-06
CBX6	22q13.1	0.55683983	5.55E-08	2.3328E-06
ENTPD5	14q24.3	0.55666569	5.62E-08	2.3499E-06
NFATC2	20q13.2	0.55629564	5.75E-08	2.3981E-06
ZNF654	3p11.1	0.55594737	5.89E-08	2.4437E-06
CD2AP	6p12.3	0.55435835	6.54E-08	2.6688E-06

PTPN11	12q24.13	0.55387947	6.75E-08	2.7395E-06
PFKFB2	1q32.1	0.55383594	6.77E-08	2.7395E-06
PACSIN2	22q13.2	0.5537271	6.82E-08	2.7535E-06
PLEKHB2	2q21.1	0.55337883	6.97E-08	2.7984E-06
RSF1	11q14.1	0.55326999	7.02E-08	2.8087E-06
ZNF41	Xp11.3	0.55292171	7.18E-08	2.8677E-06
ARFGEF1	8q13.2	0.55272581	7.28E-08	2.8931E-06
SHPRH	6q24.3	0.55244283	7.41E-08	2.9353E-06
ARID4A	14q23.1	0.55222516	7.52E-08	2.9639E-06
ZSCAN20	1p35.1	0.55209456	7.58E-08	2.9791E-06
ATF2	2q31.1	0.55185512	7.70E-08	3.0082E-06
ARFGAP3	22q13.2	0.55159391	7.83E-08	3.0479E-06
GNPTAB	12q23.2	0.55137624	7.95E-08	3.0853E-06
KIRREL1	1q23.1	0.55094089	8.17E-08	3.1616E-06
FGD6	12q22	0.55072322	8.29E-08	3.1942E-06
PRDM2	1p36.21	0.55046201	8.43E-08	3.2425E-06
GAN	16q23.2	0.55024434	8.55E-08	3.2697E-06
KIF21A	12q12	0.55009197	8.64E-08	3.2909E-06
LNPK	2q31.1	0.55004843	8.66E-08	3.2909E-06
KMT2C	7q36.1	0.55002666	8.67E-08	3.2909E-06
CARMIL1	6p22.2	0.5499396	8.72E-08	3.3032E-06
YLPM1	14q24.3	0.54985253	8.77E-08	3.3139E-06
PPARA	22q13.31	0.54983076	8.78E-08	3.3139E-06
PTPRG	3p14.2	0.54972192	8.85E-08	3.3248E-06
CCDC39	3q26.33	0.54902537	9.25E-08	3.4518E-06
DYRK2	12q15	0.548873	9.35E-08	3.4762E-06
ZNF827	4q31.21-q31	0.54882946	9.37E-08	3.4762E-06
LTN1	21q21.3	0.54869886	9.45E-08	3.4926E-06
CEP350	1q25.2	0.54865533	9.48E-08	3.496E-06
EMSY	11q13.5	0.54826351	9.72E-08	3.5706E-06
SETX	9q34.13	0.54824175	9.73E-08	3.5706E-06
PKN2	1p22.2	0.54795877	9.91E-08	3.6162E-06
SRCAP	16p11.2	0.54756696	1.02E-07	3.6748E-06
ZNF106	15q15.1	0.54739282	1.03E-07	3.7094E-06
QKI	6q26	0.54695748	1.06E-07	3.8008E-06
DNAJC13	3q22.1	0.54676157	1.07E-07	3.8349E-06
UHRF1BP1	6p21.31	0.54643506	1.09E-07	3.909E-06
ZNF318	6p21.1	0.54617386	1.11E-07	3.9677E-06
ZNF70	22q11.23	0.54580381	1.14E-07	4.041E-06
SPECC1L	22q11.23	0.5453467	1.17E-07	4.1459E-06
BAGE2	21p11.2	0.5444964	1.24E-07	4.3535E-06
PRKAR2A	3p21.31	0.54436717	1.25E-07	4.3817E-06
LRCH3	3q29	0.54432364	1.25E-07	4.3861E-06
RREB1	6p24.3	0.54404066	1.27E-07	4.45E-06
RAPH1	2q33.2	0.54367062	1.30E-07	4.5398E-06
WASHC4	12q23.3	0.54347471	1.32E-07	4.5662E-06
RAPGEF2	4q32.1	0.54340941	1.33E-07	4.5662E-06
ZFC3H1	12q21.1	0.54338764	1.33E-07	4.5662E-06

TOB2	22q13.2	0.54338764	1.33E-07	4.5662E-06
PCDH17	13q21.1	0.54338764	1.33E-07	4.5662E-06
VCPIP1	8q13.1	0.54303937	1.36E-07	4.66E-06
FNIP1	5q31.1	0.54277816	1.38E-07	4.7295E-06
FBN1	15q21.1	0.54229928	1.42E-07	4.8665E-06
TACC1	8p11.22	0.54219044	1.43E-07	4.8917E-06
CFLAR	2q33.1	0.54153742	1.49E-07	5.0455E-06
ANKRD50	4q28.1	0.54125445	1.52E-07	5.1188E-06
PRKAA1	5p13.1	0.54116738	1.53E-07	5.1366E-06
EVI5	1p22.1	0.54114561	1.53E-07	5.1366E-06
TET2	4q24	0.54108031	1.54E-07	5.1491E-06
ARHGAP5	14q12	0.53892535	1.76E-07	5.7964E-06
SPTBN1	2p16.2	0.53892535	1.76E-07	5.7964E-06
AHNAK	11q12.3	0.53877298	1.77E-07	5.8421E-06
PRRC2C	1q24.3	0.53818526	1.84E-07	6.0404E-06
CTNND1	11q12.1	0.53803289	1.86E-07	6.068E-06
CHD6	20q12	0.53777168	1.89E-07	6.1373E-06
ZMAT3	3q26.32	0.53772815	1.89E-07	6.144E-06
TBC1D5	3p24.3	0.53746694	1.92E-07	6.2345E-06
CHM	Xq21.2	0.5373581	1.94E-07	6.2666E-06
TRIP12	2q36.3	0.53711866	1.97E-07	6.3366E-06
AAK1	2p13.3	0.53707513	1.97E-07	6.3366E-06
SOCS7	17q12	0.53679216	2.01E-07	6.4282E-06
ELF1	13q14.11	0.53663978	2.03E-07	6.4668E-06
KIAA1671	22q11.23	0.53661802	2.03E-07	6.4668E-06
PJA2	5q21.3	0.53657448	2.03E-07	6.4739E-06
PXK	3p14.3	0.53618267	2.08E-07	6.6119E-06
SUSD6	14q24.1	0.53618267	2.08E-07	6.6119E-06
DMXL2	15q21.2	0.5358997	2.12E-07	6.6968E-06
DAAM1	14q23.1	0.53574733	2.14E-07	6.7495E-06
TMED10P1	8q24.3	0.53568202	2.15E-07	6.7508E-06
KLHDC10	7q32.2	0.53563849	2.16E-07	6.7508E-06
IREB2	15q25.1	0.53542082	2.19E-07	6.8116E-06
CFAP126	1q23.3	0.53537728	2.19E-07	6.8193E-06
KLHL28	14q21.2	0.5348984	2.26E-07	7.002E-06
RC3H1	1q25.1	0.53463719	2.29E-07	7.0811E-06
NIN	14q22.1	0.53455012	2.31E-07	7.0986E-06
ZEB1	10p11.22	0.53441952	2.32E-07	7.1449E-06
NEU3	11q13.4	0.53418008	2.36E-07	7.2065E-06
ATXN1	6p22.3	0.53372297	2.43E-07	7.378E-06
PRKAR1A	17q24.2	0.53354883	2.45E-07	7.4346E-06
HMBOX1	8p21.1-p12	0.53313525	2.52E-07	7.5911E-06
ZNF717	3p12.3	0.53300465	2.54E-07	7.6405E-06
PCNX1	14q24.2	0.53289581	2.55E-07	7.6671E-06
TRIM44	11p13	0.53289581	2.55E-07	7.6671E-06
AP1G1	16q22.2	0.53287404	2.56E-07	7.6671E-06
KCTD20	6p21.31	0.53259107	2.60E-07	7.7894E-06
SNORD116-4	15q11.2	0.53237989	2.63E-07	7.8553E-06

UBN1	16p13.3	0.53198158	2.70E-07	8.0129E-06
CREBBP	16p13.3	0.53191628	2.71E-07	8.033E-06
ZNF570	19q13.12	0.53145917	2.79E-07	8.2223E-06
VPS13D	1p36.22-p36	0.5314374	2.79E-07	8.2223E-06
CAMSAP2	1q32.1	0.53113266	2.84E-07	8.3518E-06
BRWD3	Xq21.1	0.53095852	2.87E-07	8.4284E-06
SEL1L	14q31.1	0.53080615	2.90E-07	8.4819E-06
DOCK9	13q32.3	0.53015313	3.02E-07	8.7484E-06
KLF12	13q22.1	0.53000076	3.05E-07	8.8041E-06
TAF2	8q24.12	0.52991369	3.06E-07	8.824E-06
CSNK1G1	15q22.31	0.52989193	3.07E-07	8.824E-06
RBL1	20q11.23	0.52958718	3.12E-07	8.9498E-06
TULP4	6q25.3	0.52958718	3.12E-07	8.9498E-06
SLIT2	4p15.31	0.52936951	3.16E-07	9.0297E-06
ADAM9	8p11.22	0.5289124	3.25E-07	9.2698E-06
NBPF14	1q21.2	0.52873826	3.29E-07	9.3412E-06
TTC37	5q15	0.52834645	3.37E-07	9.5232E-06
GCC2	2q12.3	0.52832468	3.37E-07	9.5232E-06
ATP6V1A	3q13.31	0.52832468	3.37E-07	9.5232E-06
UBR4	1p36.13	0.5277805	3.48E-07	9.7855E-06
UBR2	6p21.1	0.52769343	3.50E-07	9.8232E-06
ZHX2	8q24.13	0.52738869	3.57E-07	9.963E-06
ANKRD17	4q13.3	0.52697511	3.66E-07	1.0199E-05
ZNF148	3q21.2	0.52695334	3.66E-07	1.0199E-05
OXR1	8q23.1	0.52680097	3.69E-07	1.0278E-05
SETD2	3p21.31	0.52656153	3.75E-07	1.0413E-05
ARID4B	1q42.3	0.52640916	3.78E-07	1.0464E-05
TAF1L	9p21.1	0.52640916	3.78E-07	1.0464E-05
TGOLN2	2p11.2	0.52584321	3.91E-07	1.0796E-05
APOOL	Xq21.1	0.52575614	3.93E-07	1.0837E-05
ZNF644	1p22.2	0.52566907	3.95E-07	1.0879E-05
RGP1	9p13.3	0.525582	3.97E-07	1.0905E-05
MTMR6	13q12.13	0.52523373	4.06E-07	1.1104E-05
ARHGEF12	11q23.3	0.52508136	4.10E-07	1.1191E-05
PCLO	7q21.11	0.52497252	4.12E-07	1.1248E-05
MLXIP	12q24.31	0.5243848	4.27E-07	1.1634E-05
ZNF417	19q13.43	0.5242542	4.30E-07	1.1708E-05
PPP1R12A	12q21.2-q21	0.52423243	4.31E-07	1.1708E-05
SLK	10q24.33-q2	0.52401476	4.36E-07	1.1796E-05
PLEKHM3	2q33.3	0.52388415	4.40E-07	1.1856E-05
RAB11FIP2	10q26.11	0.52371002	4.44E-07	1.1963E-05
RBM12B	8q22.1	0.52368825	4.45E-07	1.1963E-05
ATP2B4	1q32.1	0.52342704	4.52E-07	1.2116E-05
ATXN7	3p14.1	0.52342704	4.52E-07	1.2116E-05
VCAN	5q14.2-q14.3	0.52338351	4.53E-07	1.2116E-05
SERINC1	6q22.31	0.52336174	4.54E-07	1.2116E-05
FICD	12q23.3	0.523057	4.62E-07	1.2287E-05
CASP8AP2	6q15	0.52288286	4.67E-07	1.2333E-05

TNKS2	10q23.32	0.52262165	4.74E-07	1.2492E-05
CHD8	14q11.2	0.52253458	4.77E-07	1.254E-05
SLC6A6	3p25.1	0.52242575	4.80E-07	1.2572E-05
FAM91A1	8q24.13	0.52231691	4.83E-07	1.2636E-05
UBR1	15q15.2	0.522121	4.88E-07	1.2767E-05
SLF2	10q24.31	0.52188156	4.95E-07	1.2915E-05
PPIP5K2	5q21.1	0.5218598	4.96E-07	1.2915E-05
DDX6	11q23.3	0.52155505	5.05E-07	1.3064E-05
BRCA2	13q13.1	0.52057553	5.35E-07	1.3734E-05
HUWE1	Xp11.22	0.52053199	5.36E-07	1.3734E-05
ERCC4	16p13.12	0.52053199	5.36E-07	1.3734E-05
APAF1	12q23.1	0.52042316	5.40E-07	1.3787E-05
UGCG	9q31.3	0.52042316	5.40E-07	1.3787E-05
POLK	5q13.3	0.52031432	5.43E-07	1.3858E-05
RBL2	16q12.2	0.52029255	5.44E-07	1.3858E-05
FAM157A	3q29	0.52011841	5.50E-07	1.3965E-05
SYNJ1	21q22.11	0.51998781	5.54E-07	1.4055E-05
ZNF460	19q13.43	0.51996604	5.55E-07	1.4055E-05
SMCR8	17p11.2	0.51990074	5.57E-07	1.4073E-05
RAB3GAP2	1q41	0.51987897	5.57E-07	1.4073E-05
GOLGA4	3p22.2	0.51879061	5.94E-07	1.4888E-05
ZNF573	19q13.12	0.51874708	5.96E-07	1.4907E-05
PIAS1	15q23	0.51820289	6.15E-07	1.5332E-05
ZEB2	2q22.3	0.51818113	6.16E-07	1.5332E-05
RRM2B	8q22.3	0.51807229	6.20E-07	1.541E-05
NUP155	5p13.2	0.51791992	6.25E-07	1.5509E-05
RNF24	20p13	0.51787638	6.27E-07	1.553E-05
MED23	6q23.2	0.51776755	6.31E-07	1.5609E-05
TNRC6A	16p12.1	0.51761518	6.36E-07	1.5729E-05
ZNF426	19p13.2	0.5172669	6.50E-07	1.6012E-05
ZFYVE16	5q14.1	0.51711453	6.55E-07	1.6095E-05
CBL	11q23.3	0.51711453	6.55E-07	1.6095E-05
ZNF354C	5q35.3	0.51674448	6.70E-07	1.6405E-05
RGPD5	2q13	0.51665742	6.73E-07	1.6448E-05
NUP58	13q12.13	0.51657035	6.76E-07	1.6491E-05
ZYG11B	1p32.3	0.51646151	6.81E-07	1.6575E-05
HOOK3	8p11.21	0.5160044	6.99E-07	1.6918E-05
PROX1	1q32.3	0.51583026	7.06E-07	1.7069E-05
NEK7	1q31.3	0.51532961	7.27E-07	1.7508E-05
RGPD3	2q12.2	0.51526431	7.30E-07	1.7532E-05
ATP2A2	12q24.11	0.51509017	7.37E-07	1.7667E-05
FRS2	12q15	0.51498133	7.42E-07	1.7736E-05
RBBP5	1q32.1	0.51469836	7.54E-07	1.8007E-05
ANKRD40	17q21.33	0.51426301	7.73E-07	1.8443E-05
XYLT1	16p12.3	0.51293521	8.35E-07	1.9535E-05
ITCH	20q11.22	0.51282637	8.40E-07	1.9565E-05
UEVLD	11p15.1	0.51241279	8.60E-07	1.9919E-05
CCDC117	22q12.1	0.51232573	8.64E-07	1.9996E-05

DNAJB14	4q23	0.51193391	8.84E-07	2.0402E-05
RANBP2	2q13	0.51165094	8.98E-07	2.0712E-05
PLBD2	12q24.13	0.5114115	9.11E-07	2.0901E-05
PHACTR2	6q24.2	0.51106322	9.29E-07	2.1248E-05
MTR	1q43	0.51084555	9.41E-07	2.1464E-05
FRRS1	1p21.2	0.51078025	9.44E-07	2.152E-05
ITGA1	5q11.2	0.51010546	9.81E-07	2.2262E-05
AZIN1	8q22.3	0.50973542	1.0022E-06	2.2684E-05
CALCRL	2q32.1	0.50967012	1.006E-06	2.2743E-05
SLC33A1	3q25.31	0.50960481	1.0097E-06	2.275E-05
FAM114A1	4p14	0.50949598	1.016E-06	2.2814E-05
GPATCH11	2p22.2	0.50938714	1.0223E-06	2.293E-05
NCR3LG1	11p15.1	0.50927831	1.0286E-06	2.3046E-05
MIS18BP1	14q21.2	0.5089518	1.0479E-06	2.3346E-05
STON1-GTF2	2p16.3	0.50849995	1.0751E-06	2.3792E-05
ATP9A	20q13.2	0.50829878	1.0874E-06	2.4012E-05
TMTC3	12q21.32	0.50818994	1.0941E-06	2.4133E-05
MED14	Xp11.4	0.50812464	1.0982E-06	2.4196E-05
TNS1	2q35	0.50799404	1.1063E-06	2.4322E-05
ZNF430	19p12	0.50784166	1.1159E-06	2.4478E-05
PCDHGB6	5q31.3	0.50710158	1.1635E-06	2.5331E-05
PKD2	4q22.1	0.50710158	1.1635E-06	2.5331E-05
ZNF611	19q13.41	0.50707981	1.165E-06	2.5331E-05
HCFC2	12q23.3	0.50707981	1.165E-06	2.5331E-05
PRRC1	5q23.2	0.50647033	1.2057E-06	2.6103E-05
SIN3A	15q24.2	0.50603498	1.2356E-06	2.6692E-05
GOLGB1	3q13.33	0.50596968	1.2401E-06	2.6732E-05
EPC2	2q23.1	0.50592614	1.2431E-06	2.6769E-05
CHAMP1	13q34	0.50590438	1.2447E-06	2.6773E-05
MIB1	18q11.2	0.5056867	1.26E-06	2.7073E-05
HEATR5B	2p22.2	0.50533843	1.2849E-06	2.743E-05
CNOT1	16q21	0.50531666	1.2864E-06	2.7434E-05
PPM1L	3q25.33-q26	0.50522959	1.2927E-06	2.7539E-05
SLC24A1	15q22.31	0.50503368	1.307E-06	2.7813E-05
CRIM1	2p22.2	0.50466364	1.3344E-06	2.8275E-05
SIK2	11q23.1	0.50444597	1.3507E-06	2.853E-05
MARCH6	5p15.2	0.5044242	1.3524E-06	2.8535E-05
SOCS4	14q22.3	0.50411946	1.3756E-06	2.8933E-05
TANC1	2q24.2	0.50394532	1.3891E-06	2.9155E-05
IL6R	1q21.3	0.50390179	1.3925E-06	2.9195E-05
TMEM30A	6q14.1	0.50374942	1.4044E-06	2.9352E-05
ZNF221	19q13.31	0.50374942	1.4044E-06	2.9352E-05
ANO6	12q12	0.50370588	1.4078E-06	2.9392E-05
C10ORF12	10q24.1	0.50366235	1.4112E-06	2.9433E-05
BAZ2A	12q13.3	0.5033576	1.4354E-06	2.9844E-05
VPS13A	9q21.2	0.50327053	1.4424E-06	2.9958E-05
SRFBP1	5q23.1	0.503227	1.4459E-06	2.9968E-05
ZNF704	8q21.13	0.50263928	1.494E-06	3.0837E-05

YIPF6	Xq12-q13.1	0.50261752	1.4958E-06	3.0842E-05
UBE2H	7q32.2	0.50259575	1.4976E-06	3.0847E-05
TMED5	1p22.1	0.50250868	1.5049E-06	3.0965E-05
CPED1	7q31.31	0.50220394	1.5306E-06	3.1429E-05
EDEM1	3p26.1	0.50194273	1.5529E-06	3.179E-05
ICK	6p12.1	0.50179036	1.5661E-06	3.2027E-05
TMEM106B	7p21.3	0.5008326	1.6515E-06	3.3463E-05
BIRC2	11q22.2	0.50031018	1.6998E-06	3.4338E-05
ZFYVE26	14q24.1	0.50024488	1.706E-06	3.4358E-05
RALGAPB	20q11.23	0.50022311	1.708E-06	3.4364E-05
SEC24D	4q26	0.50002721	1.7266E-06	3.4668E-05
USP49	6p21.1	0.50002721	1.7266E-06	3.4668E-05
ZNF621	3p22.1	0.49989661	1.7391E-06	3.4883E-05
KLF3	4p14	0.4996354	1.7643E-06	3.5283E-05
ST13P4	13q14.2	0.49952656	1.7749E-06	3.5353E-05
SF3B1	2q33.1	0.49920005	1.8071E-06	3.5815E-05
SP3	2q31.1	0.49915652	1.8115E-06	3.5865E-05
COL8A1	3q12.1	0.4986341	1.8642E-06	3.6728E-05
CNOT9	2q35	0.4986341	1.8642E-06	3.6728E-05
ARHGAP11B	15q13.2	0.49861234	1.8665E-06	3.6735E-05
SAMD8	10q22.2	0.49854703	1.8732E-06	3.6831E-05
COL4A3BP	5q13.3	0.4983729	1.8912E-06	3.7075E-05
RSC1A1	1p36.21	0.49802462	1.9276E-06	3.7716E-05
DEPDC5	22q12.2-q12	0.49789402	1.9415E-06	3.7949E-05
ZC3HAV1L	7q34	0.49785048	1.9461E-06	3.7965E-05
NOP9	14q12	0.49780695	1.9508E-06	3.8019E-05
MIGA1	1p31.1	0.49763281	1.9695E-06	3.8308E-05
TRAM2	6p12.2	0.49680565	2.0606E-06	3.9847E-05
ACAP2	3q29	0.49660975	2.0827E-06	4.012E-05
SLC30A1	1q32.3	0.49652268	2.0927E-06	4.0233E-05
STT3B	3p23	0.49621793	2.1277E-06	4.0868E-05
NORAD	20q11.23	0.4961744	2.1328E-06	4.0926E-05
PDS5B	13q13.1	0.4960438	2.148E-06	4.1139E-05
PTPRK	6q22.33	0.49558668	2.2021E-06	4.1933E-05
ABCA1	9q31.1	0.49549961	2.2126E-06	4.2092E-05
ZNF366	5q13.2|5q13	0.49539078	2.2257E-06	4.2302E-05
ATP13A3	3q29	0.49530371	2.2363E-06	4.2462E-05
DICER1	14q32.13	0.49506427	2.2656E-06	4.2895E-05
HERC2	15q13.1	0.4950425	2.2683E-06	4.2905E-05
JOSD1	22q13.1	0.49445478	2.3418E-06	4.4211E-05
FILIP1	6q14.1	0.49386707	2.4175E-06	4.5469E-05
ZNF121	19p13.2	0.49375823	2.4318E-06	4.5651E-05
LAMA2	6q22.33	0.49373646	2.4346E-06	4.5662E-05
MBD5	2q23.1	0.49358409	2.4548E-06	4.5953E-05
HIVEP2	6q24.2	0.49312698	2.5161E-06	4.6969E-05
SLC10A6	4q21.3	0.49309701	2.5202E-06	4.7001E-05
PHTF2	7q11.23-q21	0.49269163	2.5759E-06	4.7861E-05
ELMSAN1	14q24.3	0.49247396	2.6063E-06	4.8336E-05

SZT2	1p34.2	0.49197331	2.6775E-06	4.9564E-05
ZC3H13	13q14.13	0.49166857	2.7217E-06	5.0149E-05
STON1	2p16.3	0.49140736	2.7602E-06	5.0717E-05
FNDC1	6q25.3	0.49134206	2.7699E-06	5.0848E-05
TMED7	5q22.3	0.49081965	2.8486E-06	5.2197E-05
ARHGAP31	3q13.32-q13	0.49071081	2.8652E-06	5.2454E-05
ZNF568	19q13.12	0.48997072	2.981E-06	5.4175E-05
NNT	5p12	0.48973128	3.0194E-06	5.4772E-05
LOC283922	16q23.1	0.48968775	3.0264E-06	5.485E-05
LIG4	13q33.3	0.48951361	3.0547E-06	5.5311E-05
WASHC5	8q24.13	0.48947008	3.0618E-06	5.539E-05
TRPM7	15q21.2	0.4893177	3.0867E-06	5.569E-05
SLC5A3	21q22.11	0.48914357	3.1155E-06	5.6072E-05
DDX17	22q13.1	0.4891218	3.1192E-06	5.6072E-05
KIF27	9q21.32	0.48896943	3.1446E-06	5.6427E-05
GPR107	9q34.11	0.4881205	3.2899E-06	5.8822E-05
ATP7A	Xq21.1	0.48801167	3.309E-06	5.911E-05
BBX	3q13.12	0.48772869	3.359E-06	5.9951E-05
BAZ1B	7q11.23	0.48755455	3.3902E-06	6.0399E-05
LANCL1	2q34	0.48753279	3.3941E-06	6.0415E-05
ANP32A-IT1	15q23	0.48743749	3.4113E-06	6.0613E-05
SCYL2	12q23.1	0.48735865	3.4256E-06	6.0812E-05
ZNF142	2q35	0.48729335	3.4375E-06	6.0969E-05
SNX29P2	16p11.2	0.48683623	3.5217E-06	6.2131E-05
RPL23AP64	11q23.3	0.4867887	3.5306E-06	6.2193E-05
RUNX1T1	8q21.3	0.48675181	3.5375E-06	6.2193E-05
LDLRAD4	18p11.21	0.4867274	3.5421E-06	6.2193E-05
ADAMTS12	5p13.3-p13.2	0.48668386	3.5502E-06	6.2193E-05
CLASP2	3p22.3	0.48668386	3.5502E-06	6.2193E-05
FEM1B	15q23	0.48661856	3.5625E-06	6.2295E-05
ATF7IP	12p13.1	0.48646619	3.5913E-06	6.2474E-05
SYNE1	6q25.2	0.48587847	3.7045E-06	6.4387E-05
PARP4	13q12.12	0.4857914	3.7216E-06	6.4627E-05
MBNL3	Xq26.2	0.48524722	3.8299E-06	6.6276E-05
NUP153	6p22.3	0.48476834	3.9276E-06	6.779E-05
BMS1P4	10q22.2	0.48470304	3.9411E-06	6.7964E-05
ANKRD36B	2q11.2	0.48461597	3.9592E-06	6.8217E-05
SLC30A7	1p21.2	0.48428946	4.0277E-06	6.9218E-05
ACER2	9p22.1	0.48420239	4.0461E-06	6.9475E-05
MAP4K3	2p22.1	0.48405002	4.0786E-06	6.9852E-05
ZNF844	19p13.2	0.48405002	4.0786E-06	6.9852E-05
GAS2L3	12q23.1	0.48396295	4.0973E-06	7.0111E-05
PRDM10	11q24.3	0.48381058	4.1301E-06	7.0613E-05
DOP1A	6q14.1	0.48376705	4.1396E-06	7.0653E-05
NIPAL1	4p12	0.48367998	4.1585E-06	7.0854E-05
SNTB2	16q22.1	0.48361468	4.1727E-06	7.1037E-05
ARHGEF28	5q13.2	0.48324463	4.2544E-06	7.2241E-05
IGF2R	6q25.3	0.48311403	4.2835E-06	7.2551E-05

C6ORF89	6p21.2	0.48304873	4.2982E-06	7.2737E-05
ZNF445	3p21.31	0.48302696	4.3031E-06	7.2758E-05
EFR3A	8q24.22	0.48298342	4.3129E-06	7.2862E-05
IPP	1p34.1	0.48296166	4.3178E-06	7.2883E-05
ACBD3	1q42.12	0.48289636	4.3326E-06	7.307E-05
AKAP2	9q31.3	0.48217803	4.4981E-06	7.5479E-05
PPFIBP1	12p11.23-p1	0.48202566	4.534E-06	7.6017E-05
KIF2A	5q12.1	0.48191683	4.5598E-06	7.6321E-05
TBC1D8B	Xq22.3	0.48172092	4.6066E-06	7.7039E-05
MGAM2	7q34	0.48166275	4.6205E-06	7.7208E-05
AVPR1A	12q14.2	0.48150325	4.6591E-06	7.7787E-05
PCDHGA5	5q31.3	0.48037135	4.9412E-06	8.2222E-05
ASCC3	6q16.3	0.48013191	5.0029E-06	8.304E-05
WDR44	Xq24	0.47991424	5.0597E-06	8.3912E-05
EBF2	8p21.2	0.47985155	5.0761E-06	8.4045E-05
MTF1	1p34.3	0.47969656	5.117E-06	8.4651E-05
VEZF1	17q22	0.47952243	5.1633E-06	8.5276E-05
DDX3X	Xp11.4	0.47917415	5.2571E-06	8.6681E-05
LYST	1q42.3	0.47893471	5.3225E-06	8.7614E-05
STAM2	2q23.3	0.47880411	5.3585E-06	8.8061E-05
EXOC8	1q42.2	0.4787388	5.3766E-06	8.8213E-05
ZNF431	19p12	0.47843406	5.4617E-06	8.9463E-05
KLF13	15q13.3	0.47839053	5.474E-06	8.9516E-05
ZNF490	19p13.2-p13	0.47825164	5.5133E-06	9.0074E-05
TGFBRAP1	2q12.1-q12.2	0.47823816	5.5171E-06	9.0074E-05
DOCK5	8p21.2	0.47804225	5.5731E-06	9.0913E-05
TUG1	22q12.2	0.47795518	5.5981E-06	9.1172E-05
CAPN7	3p25.1	0.4776069	5.6993E-06	9.2592E-05
CPEB2	4p15.32	0.4776069	5.6993E-06	9.2592E-05
ARHGAP21	10p12.1|10p	0.47749807	5.7312E-06	9.296E-05
RSPRY1	16q13	0.47730216	5.7892E-06	9.3748E-05
IRF6	1q32.2	0.47691035	5.9068E-06	9.5419E-05
SRRM2	16p13.3	0.47680152	5.9399E-06	9.5798E-05
ITGA8	10p13	0.47675127	5.9552E-06	9.5967E-05
CTDSPL2	15q15.3-q21	0.47660561	5.9998E-06	9.6608E-05
MPHOSPH9	12q24.31	0.47625733	6.1078E-06	9.803E-05
KAT6A	8p11.21	0.47617026	6.1351E-06	9.8389E-05
NUFIP2	17q11.2	0.47597436	6.1969E-06	9.93E-05
PUS7L	12q12	0.47580022	6.2523E-06	9.9786E-05
CCDC93	2q14.1	0.47580022	6.2523E-06	9.9786E-05
UBR3	2q31.1	0.47573492	6.2732E-06	0.00010004
ZZEF1	17p13.2	0.47569138	6.2872E-06	0.00010018
LRRK1	15q26.3	0.47556078	6.3293E-06	0.00010061
KANSL1	17q21.31	0.47545194	6.3645E-06	0.00010101
SEC23A	14q21.1	0.47543018	6.3716E-06	0.00010104
THSD7A	7p21.3	0.47534311	6.4E-06	0.00010133
SENP7	3q12.3	0.4752778	6.4214E-06	0.00010159
ARID1A	1p36.11	0.47523427	6.4356E-06	0.00010173

ZC3HAV1	7q34	0.47447242	6.6904E-06	0.00010501
FAT4	4q28.1	0.47440711	6.7127E-06	0.00010528
YTHDF3	8q12.3	0.47432004	6.7425E-06	0.00010566
GK5	3q23	0.47425474	6.7649E-06	0.00010593
NF1	17q11.2	0.47410237	6.8176E-06	0.00010667
MTX3	5q14.1	0.47397177	6.863E-06	0.00010721
RFX7	15q21.3	0.4738847	6.8934E-06	0.0001076
FAM161B	14q24.3	0.47377586	6.9316E-06	0.00010803
TBC1D12	10q23.33	0.47344935	7.0475E-06	0.00010967
SLC25A46	5q22.1	0.47312284	7.1651E-06	0.00011132
RGPD4	2q12.3	0.47297047	7.2207E-06	0.00011192
FOXN3	14q31.3-q32	0.47297047	7.2207E-06	0.00011192
RCAN3	1p36.11	0.4728834	7.2526E-06	0.00011224
USP8	15q21.2	0.4726875	7.3249E-06	0.00011327
ANKRD12	18p11.22	0.47249159	7.3979E-06	0.00011407
TNKS	8p23.1	0.47246983	7.406E-06	0.00011407
MAGI1	3p14.1	0.47246983	7.406E-06	0.00011407
GON4L	1q22	0.47242629	7.4223E-06	0.00011407
MEF2A	15q26.3	0.47240452	7.4305E-06	0.00011411
RASEF	9q21.32	0.47225215	7.488E-06	0.00011473
MORC3	21q22.12	0.47186034	7.6377E-06	0.00011675
TASOR	3p14.3	0.47179504	7.6629E-06	0.00011705
SLC41A2	12q23.3	0.47159913	7.739E-06	0.00011794
TM9SF2	13q32.3	0.47159913	7.739E-06	0.00011794
MFSD14A	1p21.2	0.47107672	7.9455E-06	0.0001209
C9ORF129	9q22.31	0.47088081	8.0242E-06	0.00012182
ITGB3	17q21.32	0.47057607	8.1482E-06	0.00012361
CLIC4	1p36.11	0.47033663	8.2469E-06	0.00012472
AGO4	1p34.3	0.47033663	8.2469E-06	0.00012472
TMOD3	15q21.2	0.47033663	8.2469E-06	0.00012472
NIPAL2	8q22.2	0.47014073	8.3284E-06	0.00012577
HAS2	8q24.13	0.46968361	8.5217E-06	0.00012849
TEAD1	11p15.3	0.46937887	8.6528E-06	0.00013017
FOXJ3	1p34.2	0.46920473	8.7286E-06	0.00013121
KRBA2	17p13.1	0.46913943	8.7572E-06	0.00013154
PANK3	5q34	0.46903059	8.805E-06	0.00013206
SMARCAD1	4q22.3	0.46874762	8.9305E-06	0.00013344
LAMC1	1q25.3	0.46839934	9.0873E-06	0.00013558
PTPN14	1q32.3-q41	0.46824697	9.1567E-06	0.00013651
LINC00598	13q14.11	0.46818192	9.1865E-06	0.00013665
ZNF623	8q24.3	0.46818167	9.1866E-06	0.00013665
SFMBT2	10p14	0.46818167	9.1866E-06	0.00013665
ZNFX1	20q13.13	0.4680293	9.2567E-06	0.00013749
UFL1	6q16.1	0.467964	9.2869E-06	0.00013773
HELB	12q14.3|12q	0.4669627	9.7616E-06	0.00014402
PCF11	11q14.1	0.4669627	9.7616E-06	0.00014402
GOLPH3	5p13.3	0.46685387	9.8146E-06	0.00014453
VANGL1	1p13.1	0.46685387	9.8146E-06	0.00014453

AGAP1	2q37.2	0.4668321	9.8252E-06	0.00014453
ARFGEF3	6q23.3-q24.1	0.4668321	9.8252E-06	0.00014453
KIAA0586	14q23.1	0.46670149	9.8892E-06	0.00014537
EBLN2	3p13	0.46646205	1.0007E-05	0.00014689
ITPRID2	2q31.3	0.46630968	1.0083E-05	0.00014779
SLC12A6	15q14	0.46596141	1.0259E-05	0.00015014
ITPR1	3p26.1	0.4658308	1.0326E-05	0.00015089
CMTM1	16q21	0.46572197	1.0382E-05	0.00015149
ILDR2	1q24.1	0.46531976	1.059E-05	0.00015397
RAB30	11q14.1	0.46519955	1.0653E-05	0.00015477
KDR	4q12	0.46513425	1.0688E-05	0.0001551
IKZF3	17q12-q21.1	0.46507148	1.0721E-05	0.0001553
PPP4R3B	2p16.1	0.46504718	1.0734E-05	0.00015538
AGL	1p21.2	0.46496011	1.078E-05	0.00015593
USP51	Xp11.21	0.46487304	1.0827E-05	0.00015638
BRPF3	6p21.31	0.46480774	1.0862E-05	0.00015677
GNB4	3q26.33	0.46422002	1.1181E-05	0.00016126
SP4	7p15.3	0.46376291	1.1436E-05	0.00016434
EPG5	18q12.3-q21	0.46369761	1.1473E-05	0.00016475
TBL1XR1	3q26.32	0.46334933	1.1671E-05	0.00016736
AFDN	6q27	0.4633058	1.1696E-05	0.00016759
ADAM22	7q21.12	0.46324302	1.1732E-05	0.00016799
CEP290	12q21.32	0.46310989	1.1809E-05	0.00016873
CCNT2	2q21.3	0.46308812	1.1822E-05	0.00016879
HPS4	22q12.1	0.46306636	1.1834E-05	0.00016885
ZNF800	7q31.33	0.46302282	1.186E-05	0.00016909
MSI2	17q22	0.46291399	1.1923E-05	0.00016975
TSTD2	9q22.33	0.46284868	1.1961E-05	0.00017018
LUZP1	1p36.12	0.46267455	1.2064E-05	0.00017127
ZFPM2	8q23.1	0.46258748	1.2116E-05	0.00017176
SLC38A2	12q13.11	0.46245687	1.2193E-05	0.00017261
BCLAF1	6q23.3	0.46226097	1.2311E-05	0.00017416
MAGI3	1p13.2	0.46202153	1.2456E-05	0.00017596
TFRC	3q29	0.46195623	1.2496E-05	0.0001764
AP4E1	15q21.2	0.46182562	1.2576E-05	0.00017728
ZBTB1	14q23.3	0.46176032	1.2616E-05	0.00017772
JAK1	1p31.3	0.46169502	1.2657E-05	0.00017804
SPTY2D1	11p15.1	0.46160795	1.2711E-05	0.00017867
MAPK14	6p21.31	0.46088963	1.3165E-05	0.00018414
SYNJ2	6q25.3	0.46080256	1.3221E-05	0.00018479
ARL10	5q35.2	0.46051959	1.3404E-05	0.00018683
EPC1	10p11.22	0.46047605	1.3433E-05	0.0001871
NSD1	5q35.3	0.46030191	1.3547E-05	0.0001883
WAPL	10q23.2	0.46030191	1.3547E-05	0.0001883
ZBTB40	1p36.12	0.46019308	1.3619E-05	0.00018903
KIF5B	10p11.22	0.45986657	1.3837E-05	0.00019153
FAM157B	9q34.3	0.45974382	1.392E-05	0.00019254
ARL5B	10p12.31	0.45967066	1.397E-05	0.00019296

CLTC	17q23.1	0.45958359	1.4029E-05	0.00019351
PIEZO2	18p11.22-p1	0.45947476	1.4103E-05	0.0001944
PHF3	6q12	0.45912648	1.4344E-05	0.00019758
SPDYE1	7p13	0.45895234	1.4465E-05	0.00019898
TRAK2	2q33.1	0.45890881	1.4496E-05	0.00019924
KLRC4	12p13.2	0.45889625	1.4505E-05	0.00019924
PDZD8	10q25.3-q26	0.4587782	1.4588E-05	0.00020025
CMTR2	16q22.2	0.45842993	1.4836E-05	0.00020337
RBBP6	16p12.1	0.45838639	1.4867E-05	0.00020366
MAST4	5q12.3	0.45834286	1.4899E-05	0.00020395
UBXN4	2q21.3	0.45829932	1.493E-05	0.00020424
ZCCHC14	16q24.2	0.45827755	1.4946E-05	0.00020432
TAF1	Xq13.1	0.45821225	1.4993E-05	0.00020482
GBF1	10q24.32	0.45808165	1.5088E-05	0.00020584
MFHAS1	8p23.1	0.45795105	1.5184E-05	0.000207
SNRK	3p22.1	0.45753747	1.549E-05	0.00021074
TNFSF15	9q32	0.4575157	1.5506E-05	0.00021082
ADAMTSL3	15q25.2	0.45747217	1.5539E-05	0.00021098
CLCN3	4q33	0.45734156	1.5637E-05	0.00021217
COL15A1	9q22.33	0.45725449	1.5703E-05	0.00021262
HDAC9	7p21.1	0.45712389	1.5802E-05	0.00021368
LOC653653	17q23.1	0.45688445	1.5985E-05	0.00021572
SMC5	9q21.12	0.45679738	1.6053E-05	0.00021633
PTPRB	12q15	0.45655794	1.6239E-05	0.00021854
CSGALNACT2	10q11.21	0.4565144	1.6273E-05	0.00021871
DOCK4	7q31.1	0.4565144	1.6273E-05	0.00021871
IL17RA	22q11.1	0.4564491	1.6324E-05	0.0002191
ZNF813	19q13.42	0.45620966	1.6513E-05	0.00022104
SBF2	11p15.4	0.45605729	1.6634E-05	0.00022251
WWP1	8q21.3	0.45588315	1.6774E-05	0.00022423
MBTPS2	Xp22.12	0.45579608	1.6844E-05	0.00022502
WIPF2	17q21.2	0.45536074	1.72E-05	0.00022931
CUL5	11q22.3	0.45527367	1.7272E-05	0.00022996
RBFOX2	22q12.3	0.45509953	1.7417E-05	0.00023158
TAB3	Xp21.2	0.45503423	1.7471E-05	0.00023208
IPMK	10q21.1	0.45494716	1.7544E-05	0.00023265
RNF6	13q12.13	0.45475125	1.7709E-05	0.00023438
TJP1	15q13.1	0.45470772	1.7746E-05	0.00023471
GMCL1	2p13.3	0.45446828	1.7951E-05	0.00023694
PDE3A	12p12.2	0.45438121	1.8026E-05	0.00023777
PCSK5	9q21.13	0.45425061	1.8138E-05	0.00023878
C11ORF42	11p15.4	0.45413216	1.8241E-05	0.00023996
MYO5C	15q21.2	0.45412	1.8252E-05	0.00023996
RAB3GAP1	2q21.3	0.45340168	1.8888E-05	0.00024735
CEP192	18p11.21	0.45333638	1.8947E-05	0.00024795
PDPK1	16p13.3	0.45331461	1.8967E-05	0.00024805
GLCE	15q23	0.45320578	1.9065E-05	0.00024917
SULF1	8q13.2-q13.3	0.45316224	1.9105E-05	0.00024936

CARNMT1	9q21.13	0.4527269	1.9505E-05	0.00025408
SLC24A2	9p22.1-p21.3	0.45269551	1.9534E-05	0.00025411
TSSK4	14q12	0.45261806	1.9606E-05	0.00025457
TNPO1	5q13.2	0.45253099	1.9687E-05	0.00025529
ZNF660	3p21.31	0.45242215	1.9789E-05	0.00025628
KDM6A	Xp11.3	0.45237862	1.983E-05	0.00025631
IRAK3	12q14.3	0.45237862	1.983E-05	0.00025631
PGAP1	2q33.1	0.45235685	1.9851E-05	0.00025641
LARGE1	22q12.3	0.45216095	2.0036E-05	0.00025847
UTRN	6q24.2	0.45209565	2.0098E-05	0.00025904
DUSP18	22q12.2	0.45207388	2.0119E-05	0.00025904
ZNF778	16q24.3	0.45203034	2.0161E-05	0.00025924
WNK1	12p13.33	0.45192151	2.0265E-05	0.00026041
WWP2	16q22.1	0.45137733	2.0794E-05	0.0002667
HRH2	5q35.2	0.45135556	2.0816E-05	0.0002668
RBM12B-AS1	8q22.1	0.45092021	2.1249E-05	0.000272
MANEA	6q16.1	0.45074607	2.1425E-05	0.00027372
SGK3	8q13.1	0.45055017	2.1624E-05	0.00027592
SYNE3	14q32.13	0.45000599	2.2186E-05	0.00028165
CYCSP52	1q23.1	0.44996218	2.2232E-05	0.00028205
CGGBP1	3p11.1	0.44985362	2.2346E-05	0.00028314
SLC5A4	22q12.3	0.44982661	2.2375E-05	0.00028332
ZBTB24	6q21	0.44909176	2.3162E-05	0.00029199
MCTP1	5q15	0.44902646	2.3233E-05	0.00029252
SSH2	17q11.2	0.44891762	2.3352E-05	0.00029383
GTF3C4	9q34.13	0.44867818	2.3616E-05	0.00029659
WDR36	5q22.1	0.44774219	2.4676E-05	0.00030795
CELSR1	22q13.31	0.44765512	2.4777E-05	0.00030882
JMJD1C	10q21.3	0.44765512	2.4777E-05	0.00030882
EIF2AK3	2p11.2	0.44752452	2.4928E-05	0.00030994
KDM1B	6p22.3	0.44752452	2.4928E-05	0.00030994
ATF7	12q13.13	0.44741568	2.5056E-05	0.00031113
ZNF518B	4p16.1	0.4470021	2.5545E-05	0.00031661
PCDHAC2	5q31.3	0.44698033	2.5571E-05	0.00031674
INTS2	17q23.2	0.44689326	2.5675E-05	0.00031783
NPTXR	22q13.1	0.4468715	2.5701E-05	0.00031796
MAP1B	5q13.2	0.44676266	2.5832E-05	0.00031918
PHACTR4	1p35.3	0.44676266	2.5832E-05	0.00031918
KNL1	15q15.1	0.44674089	2.5858E-05	0.00031931
SOGA1	20q11.23	0.44667559	2.5937E-05	0.00032008
WWTR1	3q25.1	0.44656675	2.6069E-05	0.00032151
ABHD13	13q33.3	0.44647969	2.6175E-05	0.00032242
UBXN2B	8q12.1	0.44639262	2.6281E-05	0.00032353
TRAPPC8	18q12.1	0.44630555	2.6388E-05	0.00032445
CYB5RL	1p32.3	0.44621848	2.6496E-05	0.00032536
CEP120	5q23.2	0.44606611	2.6684E-05	0.00032708
ALS2	2q33.1	0.44595727	2.682E-05	0.00032833
NCKAP1	2q32.1	0.44595727	2.682E-05	0.00032833

PCDHGB7	5q31.3	0.44591374	2.6874E-05	0.0003288
POLR2A	17p13.1	0.44584843	2.6956E-05	0.00032939
TPP2	13q33.1	0.44582667	2.6983E-05	0.00032945
ZNF37A	10p11.1	0.4458049	2.7011E-05	0.00032945
RNF19A	8q22.2	0.44571783	2.712E-05	0.00033059
RBMS3	3p24.1	0.44569606	2.7148E-05	0.00033072
ASB7	15q26.3	0.44541309	2.7507E-05	0.00033449
TSHZ2	20q13.2	0.44519542	2.7787E-05	0.00033748
UTP23	8q24.11	0.44506481	2.7956E-05	0.00033911
BBS10	12q21.2	0.44489067	2.8183E-05	0.00034166
GABRR2	6q15	0.44473007	2.8393E-05	0.00034379
MSMP	9p13.3	0.4446077	2.8555E-05	0.00034554
HPS5	11p15.1	0.44449886	2.8699E-05	0.00034687
FAM160A1	4q31.3	0.44449886	2.8699E-05	0.00034687
BACH2	6q15	0.44443356	2.8786E-05	0.0003475
ZFHX4	8q21.13	0.44430296	2.8961E-05	0.00034918
SHROOM4	Xp11.22	0.44428119	2.899E-05	0.00034932
FNDC3A	13q14.2	0.44425942	2.9019E-05	0.00034946
CD84	1q23.3	0.44415059	2.9166E-05	0.0003508
CDC42BPA	1q42.13	0.44410705	2.9225E-05	0.0003513
DLG1	3q29	0.44386761	2.955E-05	0.00035427
LRRK2	12q12	0.44384584	2.958E-05	0.00035427
ACSL6	5q31.1	0.44383374	2.9597E-05	0.00035427
RHBDD1	2q36.3	0.44367171	2.9819E-05	0.00035651
ALDH1L2	12q23.3	0.44362817	2.9879E-05	0.00035701
WDR31	9q32	0.44325813	3.0394E-05	0.0003623
SLC25A40	7q21.12	0.44312752	3.0578E-05	0.0003634
DNAJC10	2q32.1	0.44308399	3.064E-05	0.00036391
PIK3CB	3q22.3	0.44306222	3.067E-05	0.00036406
STAG1	3q22.3	0.44288808	3.0918E-05	0.00036656
ENTPD4	8p21.3	0.44288808	3.0918E-05	0.00036656
P2RX7	12q24.31	0.44271395	3.1167E-05	0.00036907
EXOC5	14q22.3	0.44262688	3.1292E-05	0.00037034
PLAGL2	20q11.21	0.44245274	3.1544E-05	0.00037281
BAZ2B	2q24.2	0.44243097	3.1576E-05	0.00037281
PGM3	6q14.1	0.4424092	3.1607E-05	0.00037296
MON1B	16q23.1	0.44238744	3.1639E-05	0.00037311
SLC2A13	12q12	0.44232213	3.1734E-05	0.00037401
FANCM	14q21.2	0.44219153	3.1926E-05	0.00037605
MTDH	8q22.1	0.44164735	3.2734E-05	0.00038421
EDIL3	5q14.3	0.4411467	3.3495E-05	0.00039221
PCDHGA9	5q31.3	0.4410161	3.3696E-05	0.00039434
ZC3H7B	22q13.2	0.44097256	3.3764E-05	0.00039489
KLHL20	1q25.1	0.44084196	3.3966E-05	0.0003968
LARP4	12q13.12	0.44075489	3.4102E-05	0.00039792
CNTF	11q12.1	0.44063757	3.4286E-05	0.00039982
STXBP5	6q24.3	0.44047191	3.4547E-05	0.00040263
RFX3	9p24.2	0.44027601	3.4858E-05	0.00040554

SP1	12q13.13	0.44005834	3.5206E-05	0.00040936
MARCH8	10q11.21-q1	0.43997127	3.5346E-05	0.00041075
ATF6	1q23.3	0.43984066	3.5558E-05	0.00041225
ABCC4	13q32.1	0.43973183	3.5735E-05	0.00041406
GPR75	2p16.2	0.43944885	3.6199E-05	0.00041823
EIF4ENIF1	22q12.2	0.43925295	3.6524E-05	0.00042125
HERC3	4q22.1	0.43912234	3.6742E-05	0.00042303
OTUD4	4q31.21	0.43907881	3.6815E-05	0.00042362
SLC35F5	2q14.1	0.4388829	3.7145E-05	0.00042692
PIK3CG	7q22.3	0.43879583	3.7293E-05	0.0004283
UACA	15q23	0.43864346	3.7552E-05	0.00043061
ZNF585A	19q13.12	0.43838226	3.8001E-05	0.00043475
TUT7	9q21.33	0.43829519	3.8152E-05	0.00043598
DNAJB4	1p31.1	0.43820812	3.8303E-05	0.00043695
ATRN	20p13	0.43816458	3.8379E-05	0.00043757
ZMYM2	13q12.11	0.43814282	3.8417E-05	0.00043775
VCL	10q22.2	0.43799044	3.8684E-05	0.00044028
RNF213	17q25.3	0.43785984	3.8914E-05	0.00044265
NEMP1	12q13.3	0.43772924	3.9145E-05	0.00044477
ST13	22q13.2	0.43757687	3.9416E-05	0.00044709
CDK17	12q23.1	0.43757687	3.9416E-05	0.00044709
CBX5	12q13.13	0.43746803	3.9611E-05	0.00044905
SLC4A10	2q24.2	0.43741296	3.971E-05	0.00044991
SHISAL1	22q13.31	0.43738096	3.9768E-05	0.00045031
SERTAD4	1q32.2	0.43731566	3.9886E-05	0.00045139
FERMT2	14q22.1	0.43720682	4.0083E-05	0.0004531
SDE2	1q42.12	0.43716329	4.0162E-05	0.00045348
ARID1B	6q25.3	0.43716329	4.0162E-05	0.00045348
IDS	Xq28	0.43705445	4.036E-05	0.00045547
TRIM56	7q22.1	0.43688031	4.068E-05	0.00045855
GLS	2q32.2	0.43681501	4.08E-05	0.00045965
CCP110	16p12.3	0.43657557	4.1244E-05	0.00046334
LNX2	13q12.2	0.4364232	4.1529E-05	0.00046628
NBPF9	1q21.2	0.43633613	4.1693E-05	0.00046733
ZBED4	22q13.33	0.43633613	4.1693E-05	0.00046733
DNAJC21	5p13.2	0.43622729	4.1898E-05	0.00046857
DR1	1p22.1	0.43622729	4.1898E-05	0.00046857
MFSD6	2q32.2	0.43622729	4.1898E-05	0.00046857
MYO5A	15q21.2	0.43622729	4.1898E-05	0.00046857
DCAF1	3p21.2	0.43616199	4.2022E-05	0.00046969
MAP1A	15q15.3	0.43566134	4.2982E-05	0.00047908
NDUFS1	2q33.3	0.4352913	4.3704E-05	0.00048604
DHX33	17p13.2	0.43524777	4.379E-05	0.00048672
PITPNB	22q12.1	0.43511716	4.4048E-05	0.00048878
BACH1	21q21.3	0.43505186	4.4178E-05	0.00048988
OR52W1	11p15.4	0.43503134	4.4219E-05	0.00048988
COL4A4	2q36.3	0.43503009	4.4221E-05	0.00048988
ZFP92	Xq28	0.4349338	4.4413E-05	0.00049091

ZNF347	19q13.42	0.43479065	4.47E-05	0.00049381
TRIM23	5q12.3	0.43437707	4.5538E-05	0.00050216
HIPK2	7q34	0.43435531	4.5583E-05	0.00050216
CCDC163	1p34.1	0.43407233	4.6165E-05	0.00050802
ZNF107	7q11.21	0.4340288	4.6255E-05	0.00050867
SMAD9	13q13.3	0.43400703	4.63E-05	0.00050867
KDM7A	7q34	0.43391996	4.6482E-05	0.00051009
DCUN1D1	3q26.33	0.43383289	4.6663E-05	0.00051152
SPOPL	2q22.1	0.43376759	4.68E-05	0.00051246
EPB41L5	2q14.2	0.43363699	4.7075E-05	0.00051462
COBLL1	2q24.3	0.43363699	4.7075E-05	0.00051462
ABHD3	18q11.2	0.43361522	4.7121E-05	0.00051477
SLMAP	3p14.3	0.43359345	4.7167E-05	0.00051477
PRDM11	11p11.2	0.43339755	4.7582E-05	0.00051846
MAP4K5	14q22.1	0.43328871	4.7814E-05	0.00052042
RAB6D	2q21.1	0.43322341	4.7954E-05	0.00052166
TMTC2	12q21.31	0.43317987	4.8048E-05	0.0005221
VKORC1L1	7q11.21	0.43317987	4.8048E-05	0.0005221
SETD5	3p25.3	0.43294043	4.8565E-05	0.00052715
CBLB	3q13.11	0.43291867	4.8612E-05	0.00052737
DST	6p12.1	0.43278806	4.8897E-05	0.00053017
TPTEP2	22q13.1	0.4327663	4.8944E-05	0.0005304
CCDC144B	17p11.2	0.432736	4.901E-05	0.00053083
THBS4	5q14.1	0.43265746	4.9183E-05	0.00053211
CAND1	12q14.3-q15	0.43254862	4.9422E-05	0.00053354
GHRHR	7p14.3	0.43247466	4.9586E-05	0.00053473
SLC35A5	3q13.2	0.43209151	5.044E-05	0.00054131
HECW2	2q32.3	0.43143849	5.1928E-05	0.00055608
PCYT1A	3q29	0.43130789	5.2231E-05	0.00055872
LACC1	13q14.11	0.43128612	5.2281E-05	0.00055896
FAM13B	5q31.2	0.43106845	5.279E-05	0.00056379
AGO3	1p34.3	0.43098138	5.2994E-05	0.00056567
FOXN2	2p16.3	0.43093784	5.3097E-05	0.00056646
SLC35E1	19p13.11	0.43080724	5.3406E-05	0.00056915
ABHD18	4q28.2	0.4306984	5.3664E-05	0.0005716
ITGA9	3p22.2	0.43067664	5.3716E-05	0.00057178
UTP20	12q23.2	0.43065487	5.3768E-05	0.00057178
MBNL2	13q32.1	0.43065487	5.3768E-05	0.00057178
NRP2	2q33.3	0.43028482	5.4658E-05	0.00058062
SASH1	6q24.3-q25.1	0.43011069	5.5081E-05	0.00058481
GCLM	1p22.1	0.43006715	5.5187E-05	0.00058563
RBM26	13q31.1	0.42993655	5.5507E-05	0.0005884
UBE2W	8q21.11	0.42982771	5.5775E-05	0.00059061
ZNF440	19p13.2	0.42969711	5.6099E-05	0.0005934
ZNF562	19p13.2	0.42961004	5.6315E-05	0.00059532
DLEU7	13q14.3	0.42959762	5.6346E-05	0.00059532
ZNF551	19q13.43	0.42939237	5.686E-05	0.00059986
PAG1	8q21.13	0.42921823	5.7299E-05	0.00060417

MPP5	14q23.3	0.42910939	5.7575E-05	0.00060606
CELF2	10p14	0.42908762	5.763E-05	0.00060606
SCUBE3	6p21.31	0.42893525	5.8019E-05	0.00060983
CERS6	2q24.3	0.42871758	5.8579E-05	0.00061442
ZFHX3	16q22.2-q22	0.42856521	5.8974E-05	0.00061791
PCDHGB3	5q31.3	0.42847814	5.9201E-05	0.00061996
CTAGE10P	13q14.2	0.42818966	5.9958E-05	0.00062657
MXRA5	Xp22.33	0.42797749	6.0521E-05	0.00063179
RECQL	12p12.1	0.42795573	6.0579E-05	0.00063206
RNFT1	17q23.1	0.42791219	6.0695E-05	0.00063261
NBPF1	1p36.13	0.42762922	6.1455E-05	0.00063919
NCOR1	17p12-p11.2	0.42752038	6.1749E-05	0.00064159
WDR7	18q21.31	0.42745508	6.1927E-05	0.00064276
SLC35E2A	1p36.33	0.42732447	6.2283E-05	0.00064612
FAT1	4q35.2	0.42728094	6.2402E-05	0.00064702
RBM27	5q32	0.42725917	6.2462E-05	0.0006473
PLSCR4	3q24	0.42715034	6.2761E-05	0.00064871
ARHGAP42	11q22.1	0.42712857	6.2821E-05	0.000649
ANAPC1	2q13	0.42638848	6.4892E-05	0.00066901
GNA13	17q24.1	0.42627964	6.5202E-05	0.00067116
DPY19L4	8q22.1	0.42601844	6.5952E-05	0.00067782
KLF10	8q22.3	0.42601844	6.5952E-05	0.00067782
TMEM131	2q11.2	0.42571369	6.6836E-05	0.0006855
ITPR3	6p21.31	0.42556132	6.7282E-05	0.00068937
SNX19	11q24.3-q25	0.42545249	6.7603E-05	0.00069229
DOCK11	Xq24	0.42534365	6.7925E-05	0.00069523
SETD7	4q31.1	0.42523481	6.8248E-05	0.00069818
ATR	3q23	0.42503891	6.8834E-05	0.00070309
RASSF8	12p12.1	0.42499537	6.8964E-05	0.00070407
SENP6	6q14.1	0.42493007	6.9161E-05	0.00070566
MTOR	1p36.22	0.42490831	6.9227E-05	0.00070566
CCPG1	15q21.3	0.42479947	6.9556E-05	0.00070865
SMC1A	Xp11.22	0.4247777	6.9622E-05	0.00070896
KIAA0232	4p16.1	0.42473417	6.9754E-05	0.00070994
RIC1	9p24.1	0.42440766	7.0752E-05	0.00071937
LATS2	13q12.11	0.42423352	7.129E-05	0.0007241
ADAR	1q21.3	0.42399408	7.2036E-05	0.00073019
FBXL3	13q22.3	0.42399408	7.2036E-05	0.00073019
ZXDB	Xp11.21	0.42390701	7.2309E-05	0.00073258
CPSF2	14q32.12	0.42377641	7.2721E-05	0.00073638
MDC1	6p21.33	0.4237111	7.2927E-05	0.00073772
PHF8	Xp11.22	0.4237111	7.2927E-05	0.00073772
HGF	7q21.11	0.42355873	7.3411E-05	0.00074224
SF3A1	22q12.2	0.42349343	7.3619E-05	0.00074396
KPNA6	1p35.2	0.42342813	7.3828E-05	0.00074494
MAN1A1	6q22.31	0.42342813	7.3828E-05	0.00074494
ARSB	5q14.1	0.42325399	7.4388E-05	0.00074945
C3ORF38	3p11.1	0.42323222	7.4458E-05	0.00074978

MPL	1p34.2	0.42292748	7.5448E-05	0.00075898
ZNF749	19q13.43	0.42290572	7.5519E-05	0.00075931
POLH	6p21.1	0.42281865	7.5804E-05	0.00076179
RNF150	4q31.21	0.42279688	7.5876E-05	0.00076213
TEK	9p21.2	0.42264451	7.6378E-05	0.00076562
CARF	2q33.2	0.42257921	7.6594E-05	0.00076702
SIK3	11q23.3	0.42257921	7.6594E-05	0.00076702
KBTBD6	13q14.11	0.4223833	7.7246E-05	0.00077238
GSK3B	3q13.33	0.42210033	7.8196E-05	0.0007811
ZNF449	Xq26.3	0.42203502	7.8417E-05	0.00078252
RESF1	12p11.21	0.42188265	7.8935E-05	0.0007869
KDM2A	11q13.2	0.42168675	7.9605E-05	0.00079279
USP9X	Xp11.4	0.42164321	7.9755E-05	0.00079388
GMCL2	5q35.3	0.42153438	8.013E-05	0.00079722
TCF4	18q21.2	0.42140377	8.0582E-05	0.00080132
CSNK2A3	11p15.4	0.42129494	8.0961E-05	0.00080468
TASOR2	10p15.1	0.42127317	8.1037E-05	0.00080499
C2CD3	11q13.4	0.42094666	8.2185E-05	0.00081522
CLASP1	2q14.2-q14.3	0.42090312	8.2339E-05	0.00081634
ATAD2B	2p24.1-p23.3	0.42083782	8.257E-05	0.00081818
TTC33	5p13.1	0.42081606	8.2648E-05	0.00081818
MYORG	9p13.3	0.42068545	8.3113E-05	0.00082197
STK38L	12p11.23	0.42059838	8.3425E-05	0.00082465
GTF2H3	12q24.31	0.42044601	8.3973E-05	0.00082965
ASAP1	8q24.21-q24	0.4200542	8.5398E-05	0.00084165
SEMA5A	5p15.31	0.42003243	8.5478E-05	0.00084202
RFTN2	2q33.1	0.4199889	8.5638E-05	0.00084317
TLN1	9p13.3	0.41990183	8.5959E-05	0.00084591
NAPEPLD	7q22.1	0.41988006	8.6039E-05	0.00084628
ANKFY1	17p13.2	0.41961885	8.7008E-05	0.00085455
TUBGCP3	13q34	0.41953179	8.7333E-05	0.0008569
GTF2I	7q11.23	0.41951002	8.7415E-05	0.00085728
IL7R	5p13.2	0.41933588	8.8069E-05	0.00086285
ZNF397	18q12.2	0.41924881	8.8398E-05	0.00086479
TTC17	11p12-p11.2	0.41894407	8.9559E-05	0.00087528
CCDC82	11q21	0.41883523	8.9976E-05	0.00087807
NEDD1	12q23.1	0.41883523	8.9976E-05	0.00087807
ZNF587	19q13.43	0.41876993	9.0228E-05	0.0008801
ZMYM1	1p34.3	0.4187264	9.0396E-05	0.00088131
SMCHD1	18p11.32	0.41866109	9.0649E-05	0.0008829
CAB39	2q37.1	0.41855226	9.1071E-05	0.00088572
KLHL42	12p11.22	0.41837812	9.1751E-05	0.0008919
METTL24	6q21	0.41816607	9.2585E-05	0.00089913
PPP2R5E	14q23.2	0.41807338	9.2952E-05	0.00090181
SLC35E3	12q15	0.41789924	9.3645E-05	0.00090677
APPL1	3p14.3	0.41770333	9.443E-05	0.00091259
TMEM184C	4q31.23	0.41770333	9.443E-05	0.00091259
C2CD4D-AS1	1q21.3	0.41728114	9.6143E-05	0.00092735

NR1D2	3p24.2	0.41720269	9.6464E-05	0.00093
P2RY1	3q25.2	0.41713738	9.6733E-05	0.00093168
ANKS1A	6p21.31	0.41698501	9.7361E-05	0.00093729
PI4K2B	4p15.2	0.41690021	9.7713E-05	0.00093985
GCNT4	5q13.3	0.41663674	9.8813E-05	0.00094897
OCLM	1q31.1	0.4165288	9.9267E-05	0.00095241
ZNF252P	8q24.3	0.41589665	0.00010196	0.0009764
ATF1	12q13.12	0.41574428	0.00010262	0.00098225
SNORD116-2	15q11.2	0.41566626	0.00010296	0.00098503
ECM2	9q22.31	0.41554837	0.00010348	0.00098948
RAB8B	15q22.2	0.41543954	0.00010396	0.00099309
LRATD2	8q24.21	0.4152654	0.00010472	0.00099948
ZBTB10	8q21.13	0.41513479	0.0001053	0.00100453
PDLIM5	4q22.3	0.41489535	0.00010638	0.0010128
GSTCD	4q24	0.41480828	0.00010677	0.00101605
NCOA1	2p23.3	0.41443824	0.00010845	0.00103007
WDPCP	2p15	0.41443824	0.00010845	0.00103007
B3GALT2	1q31.2	0.41441647	0.00010855	0.00103052
ADGRF5	6p12.3	0.41437294	0.00010875	0.00103192
DNAJC3	13q32.1	0.41428587	0.00010915	0.00103473
SGMS2	4q25	0.41376345	0.00011157	0.00105624
PHLPP2	16q22.2	0.41374169	0.00011168	0.00105671
TCF12	15q21.3	0.41358932	0.00011239	0.00106249
ZSCAN12P1	6p22.1	0.41350225	0.00011281	0.00106488
INO80	15q15.1	0.41341518	0.00011322	0.00106777
WDR48	3p22.2	0.41337164	0.00011343	0.00106922
VPS54	2p15-p14	0.41324104	0.00011405	0.00107459
TENM4	11q14.1	0.41315397	0.00011447	0.00107748
CUL4B	Xq24	0.4131322	0.00011457	0.00107748
RPEL1	10q24.33	0.41282746	0.00011605	0.00108981
THBS2	6q27	0.41271863	0.00011658	0.00109325
ROR1	1p31.3	0.41263156	0.00011701	0.00109622
CDC73	1q31.2	0.41232681	0.00011851	0.00110979
KLHL24	3q27.1	0.41221798	0.00011905	0.00111329
HCG11	6p22.2	0.41215268	0.00011938	0.00111529
SPTLC2	14q24.3	0.41204384	0.00011992	0.00111933
FAM160B1	10q25.3	0.4116738	0.00012179	0.0011352
PROSER3	19q13.12	0.41165203	0.00012191	0.0011357
RALGAPA1	14q13.2	0.41160849	0.00012213	0.00113723
POFUT1	20q11.21	0.41152142	0.00012257	0.00114019
PCDHGB2	5q31.3	0.41151278	0.00012262	0.00114019
ZKSCAN3	6p22.1	0.41149966	0.00012268	0.00114029
IKZF2	2q34	0.41099901	0.00012528	0.0011595
DROSHA	5p13.3	0.41052013	0.0001278	0.00117905
HIP1	7q11.23	0.41052013	0.0001278	0.00117905
PCDHGA11	5q31.3	0.41047659	0.00012803	0.00118064
KAT7	17q21.33	0.41038953	0.0001285	0.00118383
RAI14	5p13.2	0.41010655	0.00013002	0.00119675

INHBA	7p14.1	0.40988888	0.0001312	0.00120596
MPHOSPH8	13q12.11	0.40984534	0.00013144	0.00120759
SACM1L	3p21.31	0.40982358	0.00013156	0.00120812
RNGTT	6q15	0.40980181	0.00013168	0.00120866
CRTC3	15q26.1	0.40962767	0.00013264	0.00121686
MYO6	6q14.1	0.4096059	0.00013276	0.00121686
NECTIN3	3q13.13	0.40951883	0.00013324	0.00122014
ZFP30	19q13.12	0.40951883	0.00013324	0.00122014
IPO8	12p11.21	0.40949707	0.00013336	0.00122068
FKBP14	7p14.3	0.4093447	0.0001342	0.00122674
RAD50	5q31.1	0.4093447	0.0001342	0.00122674
FAM217B	20q13.33	0.40914879	0.0001353	0.00123503
MCM8	20p12.3	0.40912702	0.00013542	0.00123503
WASF2	1p36.11	0.40912702	0.00013542	0.00123503
DOCK1	10q26.2	0.40906172	0.00013579	0.00123725
DLC1	8p22	0.40869168	0.00013789	0.00125522
CD300E	17q25.1	0.40851812	0.00013888	0.00126254
FAM168B	2q21.1	0.40845224	0.00013926	0.00126541
FEM1C	5q22.3	0.4083434	0.00013989	0.00126938
MN1	22q12.1	0.4081475	0.00014103	0.00127913
SOS1	2p22.1	0.40788629	0.00014256	0.00129066
SESN3	11q21	0.40777745	0.0001432	0.00129589
ZBTB26	9q33.2	0.40760331	0.00014423	0.00130465
ITGB1	10p11.22	0.40755978	0.00014449	0.0013064
ABCB10	1q42.13	0.40740741	0.0001454	0.00131225
DOC2B	17p13.3	0.40728808	0.00014612	0.00131753
STAG2	Xq25	0.40710267	0.00014724	0.00132704
TEP1	14q11.2	0.40705913	0.00014751	0.00132822
KCND3	1p13.2	0.4068767	0.00014862	0.00133643
GTPBP1	22q13.1	0.40671085	0.00014964	0.00134457
DLX6-AS1	7q21.3	0.40670735	0.00014966	0.00134457
YAP1	11q22.1	0.40644965	0.00015125	0.00135584
WDFY1	2q36.1	0.40644965	0.00015125	0.00135584
CREB3L2	7q33	0.40621021	0.00015275	0.00136864
MMS22L	6q16.1	0.40612314	0.0001533	0.00137292
CEP152	15q21.1	0.40597077	0.00015426	0.00137968
RASA1	5q14.3	0.40590546	0.00015467	0.00138214
TBC1D2B	15q24.3-q25	0.40590546	0.00015467	0.00138214
TLL1	4q32.3	0.40584016	0.00015509	0.00138399
SAMD4A	14q22.2	0.40575309	0.00015564	0.0013877
BRIP1	17q23.2	0.40573133	0.00015578	0.0013877
PRRC2B	9q34.13	0.40573133	0.00015578	0.0013877
KIF26B	1q44	0.40540482	0.00015788	0.00140327
MBL2	10q21.1	0.40526113	0.00015882	0.0014103
UNC5C	4q22.3	0.40523068	0.00015901	0.00141143
CCDC144A	17p11.2	0.40521773	0.0001591	0.00141143
ZNF845	19q13.42	0.40518714	0.0001593	0.00141206
ZNF268	12q24.33	0.40505654	0.00016015	0.00141837

SLC7A11	4q28.3	0.40505654	0.00016015	0.00141837
RFC1	4p14	0.40499124	0.00016058	0.00142154
SORBS1	10q24.1	0.40470826	0.00016245	0.0014349
ARPP19	15q21.2	0.40462119	0.00016303	0.00143938
RSBN1	1p13.2	0.40459943	0.00016318	0.00144003
AREL1	14q24.3	0.40451236	0.00016376	0.00144452
GFPT1	2p13.3	0.40449059	0.0001639	0.00144517
NIPAL3	1p36.11	0.40442529	0.00016434	0.00144775
DIP2B	12q13.12	0.40438175	0.00016463	0.00144968
TMEM200A	6q23.1	0.40433822	0.00016493	0.00145162
ZXDA	Xp11.21	0.40418585	0.00016596	0.0014594
MAGT1	Xq21.1	0.40409878	0.00016655	0.00146395
PCDHGA4	5q31.3	0.40407701	0.0001667	0.0014646
ZNF91	19p12	0.40405525	0.00016684	0.00146526
STARD13	13q13.1-q13	0.40383757	0.00016833	0.00147768
OTUD7B	1q21.2	0.40364167	0.00016968	0.0014856
NPR3	5p13.3	0.4036199	0.00016983	0.00148626
BTN2A3P	6p22.2	0.4034893	0.00017074	0.00149288
TYW5	2q33.1	0.40346753	0.00017089	0.00149355
AGO1	1p34.3	0.40342399	0.00017119	0.00149555
PCNX4	14q23.1	0.40340223	0.00017135	0.00149622
CCDC134	22q13.2	0.40331516	0.00017195	0.00150022
MAP3K13	3q27.2	0.40331516	0.00017195	0.00150022
ZNRF2P1	7p14.3	0.40320632	0.00017272	0.00150556
ZNF451	6p12.1	0.40296688	0.00017441	0.00151698
ZSCAN23	6p22.1	0.40293212	0.00017466	0.00151847
MTMR10	15q13.3	0.40274921	0.00017596	0.00152714
PICALM	11q14.2	0.40259684	0.00017705	0.00153528
NEK4	3p21.1	0.40253154	0.00017752	0.00153869
FRMD6	14q22.1	0.402488	0.00017784	0.00154074
TMX1	14q22.1	0.4022921	0.00017926	0.00155237
MAP3K9	14q24.2	0.40224856	0.00017958	0.00155444
GPR180	13q32.1	0.40222679	0.00017973	0.00155514
C5ORF24	5q31.1	0.40194382	0.00018181	0.00156833
ZBTB6	9q33.2	0.40139964	0.00018586	0.0016019
CHD1	5q15-q21.1	0.40126903	0.00018685	0.00160899
ZBTB39	12q13.3	0.4012255	0.00018718	0.00161113
FAM199X	Xq22.2	0.40107313	0.00018833	0.00162039
ANGPTL5	11q22.1	0.40089553	0.00018969	0.00163066
PTPN1	20q13.13	0.40083369	0.00019016	0.00163333
SENP1	12q13.11	0.40079015	0.0001905	0.0016355
ANKRD13C	1p31.1	0.40076839	0.00019067	0.00163623
TAB2	6q25.1	0.40072485	0.000191	0.0016377
PDS5A	4p14	0.40061601	0.00019184	0.0016435
FRMD3	9q21.32	0.40039834	0.00019354	0.00165444
TBC1D23	3q12.1-q12.2	0.40037657	0.00019371	0.00165519
MFN1	3q26.33	0.4001589	0.00019541	0.00166692
SLC39A10	2q32.3	0.40005006	0.00019627	0.0016721

RNF152	18q21.33	0.40000653	0.00019662	0.00167432
STRN3	14q12	0.399963	0.00019696	0.00167655
DENND2C	1p13.2	0.39985416	0.00019783	0.00168104
GPLD1	6p22.3	0.39985416	0.00019783	0.00168104
YY2	Xp22.12	0.39981062	0.00019818	0.00168327
NAIP	5q13.2	0.39974532	0.0001987	0.00168698
PRKDC	8q11.21	0.39957118	0.0002001	0.00169668
CEMIP2	9q21.13	0.39957118	0.0002001	0.00169668
PDP2	16q22.1	0.39948412	0.0002008	0.00170191
LEMD3	12q14.3	0.3990923	0.00020399	0.0017238
PRPF40A	2q23.3	0.3990923	0.00020399	0.0017238
PNMA2	8p21.2	0.39907054	0.00020416	0.00172458
EVC	4p16.2	0.39898347	0.00020488	0.00172842
TRPS1	8q23.3	0.39891817	0.00020542	0.00173223
BICD1	12p11.21	0.3988964	0.0002056	0.00173301
FAM135A	6q13	0.3988311	0.00020614	0.00173682
LIPI	21q11.2	0.39869878	0.00020724	0.00174534
NAA25	12q24.13	0.39854812	0.00020849	0.00175518
MSL2	3q22.3	0.39852635	0.00020867	0.00175597
ARIH1	15q24.1	0.39850459	0.00020886	0.00175602
PLEKHF2	8q22.1	0.39850459	0.00020886	0.00175602
SETD1B	12q24.31	0.39841752	0.00020959	0.00175993
ATL3	11q13.1	0.39828691	0.00021069	0.00176693
PCDHGC4	5q31.3	0.39814321	0.0002119	0.00177639
SUZ12	17q11.2	0.39809101	0.00021235	0.00177861
NBEA	13q13.3	0.39806924	0.00021253	0.00177941
UBP1	3p22.3	0.39802571	0.0002129	0.00178177
MARCH3	5q23.2	0.39800394	0.00021309	0.00178258
TRAF6	11p12	0.39787334	0.00021421	0.00178967
EP400	12q24.33	0.3978298	0.00021458	0.00179128
PALB2	16p12.2	0.3978298	0.00021458	0.00179128
DYNC1LI2	16q22.1	0.39752506	0.00021721	0.00181174
ZNF708	19p12	0.39752506	0.00021721	0.00181174
ZNF718	4p16.3	0.39728562	0.0002193	0.00182839
NOMO2	16p12.3	0.39713325	0.00022064	0.00183802
G2E3	14q12	0.39680674	0.00022353	0.00185824
CPEB4	5q35.2	0.39674144	0.00022412	0.00186076
NBN	8q21.3	0.39674144	0.00022412	0.00186076
AKAP10	17p11.2	0.39637139	0.00022745	0.00188213
TCAF1	7q35	0.39611019	0.00022983	0.00190023
ZNF236	18q23	0.39602312	0.00023062	0.00190604
PUM1	1p35.2	0.39587075	0.00023203	0.00191683
SLC39A14	8p21.3	0.39565307	0.00023404	0.00193189
P2RY4	Xq13.1	0.39527904	0.00023755	0.00195919
TNFAIP3	6q23.3	0.39523949	0.00023792	0.00196145
GMFB	14q22.2	0.39495652	0.00024061	0.00197958
CDK13	7p14.1	0.39493475	0.00024081	0.00197958
ENTPD7	10q24.2	0.39493475	0.00024081	0.00197958

RABGAP1L	1q25.1	0.39473885	0.00024269	0.00199173
MTRR	5p15.31	0.39471708	0.0002429	0.00199262
BTBD1	15q25.2	0.39456471	0.00024437	0.00200304
PLD1	3q26.31	0.39454294	0.00024458	0.00200394
LPIN2	18p11.31	0.3944341	0.00024564	0.00201012
UBE4A	11q23.3	0.39439057	0.00024606	0.00201276
RNF217	6q22.31	0.39432527	0.0002467	0.00201631
RAD1	5p13.2	0.39421643	0.00024776	0.00202419
CALU	7q32.1	0.3941729	0.00024819	0.00202601
PIGK	1p31.1	0.39406406	0.00024926	0.00203226
BLOC1S6	15q21.1	0.39397699	0.00025012	0.00203843
PDE8A	15q25.3	0.39391169	0.00025077	0.00204287
MYH9	22q12.3	0.39388992	0.00025098	0.00204379
SLC23A2	20p13	0.39378109	0.00025207	0.00205008
UBE3B	12q24.11	0.39378109	0.00025207	0.00205008
ZNF720	16p11.2	0.39373755	0.0002525	0.00205194
ETS1	11q24.3	0.39349811	0.0002549	0.00206974
PCDH18	4q28.3	0.39330221	0.00025688	0.00208411
FGD4	12p11.21	0.39328044	0.0002571	0.00208505
HSPG2	1p36.12	0.3931063	0.00025887	0.00209857
NT5DC3	12q23.3	0.39299746	0.00025998	0.00210502
KCNT2	1q31.3	0.3929757	0.00026021	0.00210511
USP24	1p32.3	0.3929757	0.00026021	0.00210511
TMEM245	9q31.3	0.39295393	0.00026043	0.00210607
ATP6V1C1	8q22.3	0.39288863	0.0002611	0.00211064
ZHX1	8q24.13	0.39262742	0.0002638	0.00212987
DCAF17	2q31.1	0.39260565	0.00026403	0.00213083
POLDIP3	22q13.2	0.39258389	0.00026425	0.00213179
JMY	5q14.1	0.39254035	0.00026471	0.00213459
CRAMP1	16p13.3	0.39247505	0.00026539	0.00213921
KIF3B	20q11.21	0.39240975	0.00026607	0.00214298
TRERF1	6p21.1	0.39240975	0.00026607	0.00214298
RRAGC	1p34.3	0.39225738	0.00026767	0.00215325
RSPO2	8q23.1	0.39223859	0.00026787	0.00215336
PAK2	3q29	0.39223561	0.0002679	0.00215336
SMAD3	15q22.33	0.39221384	0.00026813	0.00215433
ZNF501	3p21.31	0.39212677	0.00026905	0.00215997
CEP170	1q43	0.39210501	0.00026928	0.00216095
NBR1	17q21.31	0.39199617	0.00027043	0.00216934
LPAR4	Xq21.1	0.39186557	0.00027182	0.00217874
GAB1	4q31.21	0.39180026	0.00027252	0.00218346
EAF1	3p25.1	0.39162613	0.00027439	0.00219491
PLCB1	20p12.3	0.39160436	0.00027463	0.00219502
SREBF2	22q13.2	0.39160436	0.00027463	0.00219502
PAPPA2	1q25.2	0.39149319	0.00027583	0.00220196
TCEANC	Xp22.2	0.39125608	0.0002784	0.00221986
WDR82	3p21.2	0.39112548	0.00027983	0.00223036
ZBTB43	9q33.3	0.39095134	0.00028175	0.00224383

MAP3K7	6q15	0.3909078	0.00028223	0.00224676
BNC2	9p22.3-p22.2	0.3908425	0.00028295	0.00225161
RASGRF2	5q14.1	0.39079897	0.00028343	0.00225455
ZFP14	19q13.12	0.3905813	0.00028586	0.00227202
DOCK10	2q36.2	0.39023302	0.00028978	0.00229859
SPATA6	1p33	0.39018948	0.00029027	0.00230158
NEK1	4q33	0.39016772	0.00029052	0.00230262
SEC23IP	10q26.11-q2	0.39010242	0.00029126	0.00230759
CMTR1	6p21.2	0.39008065	0.00029151	0.00230781
C2CD6	2q33.1	0.39007957	0.00029152	0.00230781
BTBD19	1p34.1	0.39003711	0.000292	0.00231072
THBS1	15q14	0.38995004	0.000293	0.00231767
MTM1	Xq28	0.38988474	0.00029374	0.00232266
TMEM19	12q21.1	0.38979767	0.00029474	0.00232964
MMGT1	Xq26.3	0.38960177	0.000297	0.00234565
ADCY5	3q21.1	0.38925349	0.00030106	0.00237488
PRLR	5p13.2	0.38903582	0.00030363	0.00239131
TRIM25	17q22	0.38888345	0.00030543	0.00240363
RNF185	22q12.2	0.38870931	0.00030751	0.00241901
TGFBR1	9q22.33	0.38860047	0.00030881	0.00242735
C3ORF70	3q27.2	0.38840457	0.00031117	0.00244108
BRD2	6p21.32	0.3883175	0.00031223	0.00244742
PI4KA	22q11.21	0.38823043	0.00031328	0.00245474
KCNQ3	8q24.22	0.38794745	0.00031674	0.0024799
NR3C1	5q31.3	0.38783862	0.00031808	0.00248843
ZNF609	15q22.31	0.38783862	0.00031808	0.00248843
CDK19	6q21	0.38768625	0.00031997	0.00250023
ZNF462	9q31.2	0.38759918	0.00032105	0.0025077
CDYL2	16q23.2	0.38755564	0.00032159	0.00251095
PGM5P2	9p11.2	0.38752799	0.00032193	0.00251265
PRKAB2	1q21.1	0.38749034	0.0003224	0.00251533
PRR11	17q22	0.38740327	0.00032349	0.00251988
PPP1R12B	1q32.1	0.3873815	0.00032377	0.00252004
GALNT5	2q24.1	0.38735974	0.00032404	0.00252118
DOCK7	1p31.3	0.3873162	0.00032459	0.00252444
ZNF484	9q22.31	0.38722913	0.00032568	0.00253198
EGFR	7p11.2	0.38716383	0.00032651	0.0025364
ZC3H7A	16p13.13	0.38688086	0.0003301	0.00256132
NSD3	8p11.23	0.38677202	0.00033149	0.00257112
UNC13B	9p13.3	0.38672849	0.00033205	0.00257344
ZFP1	16q23.1	0.38668495	0.00033261	0.00257677
SRGAP1	12q14.2	0.38635844	0.00033683	0.00260744
FAM216B	13q14.11	0.38620516	0.00033883	0.00261883
SH2B3	12q24.12	0.38614077	0.00033967	0.00262433
DBT	1p21.2	0.38601017	0.00034138	0.00263553
KDM5A	12p13.33	0.38583603	0.00034368	0.00265225
ARCN1	11q23.3	0.38581426	0.00034397	0.00265345
PRELP	1q32.1	0.38572719	0.00034513	0.00266133

TTC14	3q26.33	0.38570542	0.00034542	0.00266254
NAV1	1q32.1	0.38561835	0.00034658	0.00267045
SPRTN	1q42.2	0.38553129	0.00034774	0.00267838
NUAK1	12q23.3	0.38548775	0.00034832	0.00268183
TBC1D16	17q25.3	0.38544422	0.00034891	0.00268529
WSCD2	12q23.3	0.38521486	0.000352	0.002707
NDC1	1p32.3	0.38520478	0.00035214	0.002707
MFAP3	5q33.2	0.38518301	0.00035243	0.00270718
UBE2Q2	15q24.2	0.38496534	0.00035539	0.00272677
KIAA1107	1p22.1	0.38490003	0.00035628	0.00273257
BCLAF3	Xp22.12	0.38483473	0.00035718	0.00273732
DIS3	13q21.33	0.38481296	0.00035748	0.00273751
RAB23	6p12.1-p11.2	0.3847259	0.00035868	0.00274351
MOB1A	2p13.1	0.38466059	0.00035958	0.00274829
PXT1	6p21.31	0.38464973	0.00035973	0.00274838
SPAST	2p22.3	0.38446469	0.00036229	0.00276478
PCARE	2p23.2	0.38440964	0.00036306	0.00276957
MTMR9	8p23.1	0.38439939	0.0003632	0.00276959
DIXDC1	11q23.1	0.38431232	0.00036441	0.00277779
SVEP1	9q31.3	0.38429055	0.00036472	0.00277905
JCAD	10p11.23	0.38424702	0.00036533	0.0027805
PAWR	12q21.2	0.38424702	0.00036533	0.0027805
RORA	15q22.2	0.38413818	0.00036685	0.00278999
TTN	2q31.2	0.38400758	0.00036869	0.00280078
TOR1AIP1	1q25.2	0.38392051	0.00036993	0.00280907
KIDINS220	2p25.1	0.38389874	0.00037023	0.00280927
ITPR2	12p11.23	0.38389874	0.00037023	0.00280927
KIAA1217	10p12.2-p12	0.38383344	0.00037116	0.00281523
PRPF4B	6p25.2	0.38361576	0.00037427	0.00283446
TLR6	4p14	0.38350693	0.00037583	0.00284413
GAS7	17p13.1	0.38348516	0.00037614	0.00284542
GASK1B	4q32.1	0.38346339	0.00037645	0.00284562
SAMHD1	20q11.23	0.38346339	0.00037645	0.00284562
ZNF592	15q25.3	0.38341986	0.00037708	0.00284928
PLEKHG1	6q25.1	0.38337632	0.00037771	0.00285187
ATP8B2	1q21.3	0.38320219	0.00038023	0.00286765
CLIC5	6p21.1	0.38320219	0.00038023	0.00286765
CTDSP2	12q14.1	0.38283214	0.00038564	0.00290406
IFT80	3q25.33	0.38281037	0.00038596	0.00290538
RAB21	12q21.1	0.38278861	0.00038628	0.0029056
CDC42BPB	14q32.32	0.38276684	0.00038661	0.00290691
RBM7	11q23.2	0.38274507	0.00038693	0.00290823
KIF1B	1p36.22	0.38267977	0.00038789	0.00291439
MICAL2	11p15.3	0.38263624	0.00038854	0.00291813
FGF10	5p12	0.3825527	0.00038978	0.00292453
GAB2	11q14.1	0.38250563	0.00039048	0.00292718
NAA30	14q22.3	0.38235326	0.00039275	0.00294202
TRANK1	3p22.2	0.38233149	0.00039308	0.00294335

TRIM33	1p13.2	0.38228796	0.00039373	0.00294492
INTS4P2	7q11.21	0.38222266	0.00039471	0.00295114
AKAP12	6q25.1	0.38220089	0.00039504	0.00295138
CFAP97	4q35.1	0.38220089	0.00039504	0.00295138
TBC1D15	12q21.1	0.38213559	0.00039602	0.00295762
HAS2-AS1	8q24.13	0.38205843	0.00039719	0.00296521
HOOK1	1p32.1	0.38202675	0.00039767	0.00296545
PAFAH1B1	17p13.3	0.38193968	0.00039899	0.00297307
ZNF675	19p12	0.38187438	0.00039998	0.00297935
HMCN1	1q25.3-q31.1	0.38183085	0.00040064	0.00298317
OTUD6B	8q21.3	0.38174378	0.00040197	0.00299195
PCDHGA2	5q31.3	0.38163494	0.00040364	0.00299988
ZNF625	19p13.2	0.3814608	0.00040632	0.00301642
ZNF689	16p11.2	0.38143904	0.00040666	0.00301667
MARCH1	4q32.2-q32.3	0.38130843	0.00040868	0.00302942
GLIS3	9p24.2	0.38106899	0.00041241	0.00305254
KAT6B	10q22.2	0.38106899	0.00041241	0.00305254
CALHM5	6q22.1	0.38100369	0.00041343	0.00305784
CNTN5	11q22.1	0.38077816	0.00041699	0.00308297
DENND6A	3p14.3	0.38074248	0.00041755	0.00308371
SAMD9	7q21.2	0.38074248	0.00041755	0.00308371
RGS17	6q25.2	0.38067718	0.00041858	0.00308906
ITGA4	2q31.3	0.38052481	0.00042101	0.00310465
DDX21	10q22.1	0.38024183	0.00042554	0.0031323
BLZF1	1q24.2	0.38022007	0.0004259	0.00313256
DYNC1H1	14q32.31	0.38022007	0.0004259	0.00313256
LRIG2	1p13.2	0.37989356	0.00043119	0.00316682
ATP11B	3q26.33	0.37976295	0.00043332	0.00317781
CDC27	17q21.32	0.37976295	0.00043332	0.00317781
NOL9	1p36.31	0.37965412	0.00043511	0.00318766
DAG1	3p21.31	0.37963235	0.00043547	0.00318766
NPTN	15q24.1	0.37963235	0.00043547	0.00318766
RAPGEF5	7p15.3	0.37945821	0.00043834	0.00320634
PLA2R1	2q24.2	0.379197	0.00044268	0.00323454
ALG10B	12q12	0.37917524	0.00044305	0.00323601
PLEKHA7	11p15.2-p15	0.3790011	0.00044597	0.00325495
CELF1	11p11.2	0.37891403	0.00044743	0.00326445
ERC1	12p13.33	0.37889226	0.0004478	0.00326594
LRRC37A4P	17q21.31	0.37880519	0.00044927	0.00327486
SLC39A9	14q24.1	0.37878343	0.00044964	0.00327486
LRRC8B	1p22.2	0.37876166	0.00045001	0.00327486
GLG1	16q23.1	0.37876166	0.00045001	0.00327486
RNF38	9p13.2	0.37876166	0.00045001	0.00327486
NFIC	19p13.3	0.37876166	0.00045001	0.00327486
SASS6	1p21.2	0.37871812	0.00045075	0.00327903
PTAFR	1p35.3	0.37869636	0.00045112	0.00327933
PTPN13	4q21.3	0.37867459	0.00045149	0.00328082
LRP1	12q13.3	0.37841338	0.00045595	0.00331083

ZNF184	6p22.1	0.37819571	0.0004597	0.00333683
PTPRD	9p24.1-p23	0.37817394	0.00046007	0.00333835
GNL3L	Xp11.22	0.37813041	0.00046083	0.0033426
SYDE2	1p22.3	0.37804334	0.00046234	0.00335234
SNORD116-2	15q11.2	0.3779955	0.00046317	0.00335715
GP5	3q29	0.37796373	0.00046373	0.00335994
LIMD1	3p21.31	0.3778692	0.00046538	0.00337067
ABCC9	12p12.1	0.37778213	0.0004669	0.0033768
ZSCAN12	6p22.1	0.37771683	0.00046805	0.00338386
ADD3	10q25.1-q25	0.37762976	0.00046958	0.00339371
UBN2	7q34	0.37752092	0.0004715	0.00340637
LRRC8C	1p22.2	0.37734679	0.00047459	0.00342373
TXLNG	Xp22.2	0.37734679	0.00047459	0.00342373
NHLRC3	13q13.3	0.37682437	0.00048397	0.00348258
ZNF680	7q11.21	0.3767373	0.00048555	0.00349143
WDR47	1p13.3	0.37671553	0.00048595	0.00349302
FBXO38	5q32	0.376672	0.00048674	0.00349745
CYLD	16q12.1	0.37638902	0.00049192	0.00353212
MET	7q31.2	0.37636726	0.00049232	0.00353245
MYO1E	15q22.2	0.37636726	0.00049232	0.00353245
GCN1	12q24.23	0.37628019	0.00049393	0.00354269
HMGCS1	5p12	0.37621489	0.00049513	0.00355007
CCR4	3p22.3	0.37581424	0.0005026	0.0035997
SOCS5	2p21	0.3756707	0.0005053	0.00361288
GALNT4	12q21.33	0.37564894	0.00050571	0.00361288
GNS	12q14.3	0.37564894	0.00050571	0.00361288
LIN54	4q21.22	0.3756054	0.00050653	0.00361745
GCLC	6p12.1	0.37556187	0.00050735	0.00362203
STS	Xp22.31	0.37549657	0.00050859	0.00362826
ATP11A	13q34	0.3754748	0.000509	0.00362991
SNORA71E	20q11.23	0.37522176	0.00051383	0.00366037
PHF20L1	8q24.22	0.37508299	0.00051649	0.00367408
WRN	8p12	0.37499592	0.00051817	0.00368207
NSF	17q21.31	0.37499592	0.00051817	0.00368207
SPATA46	1q23.3	0.3748707	0.00052059	0.00369737
DHX36	3q25.2	0.37486531	0.00052069	0.00369737
RAB39A	11q22.3	0.37480001	0.00052196	0.00370372
ZNF136	19p13.2	0.37477825	0.00052238	0.0037054
TENT5A	6q14.1	0.37475648	0.0005228	0.00370595
ADORA2A-AS	22q11.23	0.37475511	0.00052283	0.00370595
ZNF518A	10q24.1	0.37466941	0.0005245	0.00371514
QSER1	11p13	0.37458234	0.0005262	0.00372454
ARL13B	3q11.1-q11.2	0.37451704	0.00052748	0.00372961
FRY	13q13.1	0.37449527	0.00052791	0.00373131
CYBRD1	2q31.1	0.37442997	0.00052919	0.00373905
ALKBH8	11q22.3	0.37438643	0.00053005	0.00374377
FAM205A	9p13.3	0.37437288	0.00053031	0.00374433
SRRM1	1p36.11	0.37429937	0.00053176	0.00375058

FBXO30	6q24.3	0.37425583	0.00053262	0.00375266
TMEM87B	2q13	0.37425583	0.00053262	0.00375266
EXPH5	11q22.3	0.37419053	0.00053392	0.00375912
ZNRD1ASP	6p22.1	0.37412523	0.00053521	0.00376425
SLC7A1	13q12.3	0.37412523	0.00053521	0.00376425
TMPO	12q23.1	0.37390755	0.00053955	0.00379076
UBL3	13q12.3	0.37384225	0.00054086	0.00379728
ANKIB1	7q21.2	0.37375518	0.00054261	0.00380687
UGGT2	13q32.1	0.37360281	0.00054568	0.00382171
USP38	4q31.21	0.37360281	0.00054568	0.00382171
ZNF510	9q22.33	0.37358105	0.00054612	0.00382345
PAN3	13q12.2	0.37349398	0.00054789	0.00383445
VGLL3	3p12.1	0.37345044	0.00054877	0.00383795
ZNF507	19q13.11	0.37345044	0.00054877	0.00383795
PPP1R15B	1q32.1	0.37342867	0.00054922	0.00383835
ZNF225	19q13.31	0.37342867	0.00054922	0.00383835
DDX46	5q31.1	0.3732763	0.00055232	0.00385736
C20ORF194	20p13	0.37310216	0.0005559	0.00388094
IGFBP5	2q35	0.3730804	0.00055634	0.00388271
LY75	2q24.2	0.37305863	0.00055679	0.00388447
PROS1	3q11.1	0.37290626	0.00055994	0.00390506
CRYBG1	6q21	0.37268859	0.00056446	0.00393248
NR6A1	9q33.3	0.37253622	0.00056765	0.00395329
RBSN	3p25.1	0.37251445	0.0005681	0.003955
MARCH7	2q24.2	0.37249268	0.00056856	0.0039555
MBNL1	3q25.1-q25.2	0.37247091	0.00056902	0.0039573
BAG4	8p11.23	0.37242738	0.00056993	0.00395952
TTC30B	2q31.2	0.37242738	0.00056993	0.00395952
PLEKHA1	10q26.13	0.37229678	0.00057269	0.0039745
ZNF701	19q13.41	0.37229678	0.00057269	0.0039745
TMTC1	12p11.22	0.37223147	0.00057407	0.0039827
SCYL3	1q24.2	0.37218794	0.00057499	0.00398678
SIM1	6q16.3	0.37218488	0.00057506	0.00398678
SLC30A5	5q13.1-q13.2	0.37216617	0.00057546	0.00398814
HMG20A	15q24.3	0.37212264	0.00057638	0.00399177
PRKD3	2p22.2	0.3720791	0.00057731	0.00399584
CRYBG3	3q11.2	0.37205734	0.00057777	0.00399584
ZFP91-CNTF	11q12.1	0.37192751	0.00058054	0.00401084
CD109	6q13	0.37170906	0.00058523	0.00403905
KLHL3	5q31.2	0.37160022	0.00058758	0.00405387
RPS6KA6	Xq21.1	0.37143153	0.00059125	0.0040757
PTGER4	5p13.1	0.37131725	0.00059374	0.00409065
DEK	6p22.3	0.37127371	0.00059469	0.00409579
CLSPN	1p34.3	0.37120841	0.00059612	0.0041028
DCP1A	3p21.1	0.37112134	0.00059803	0.00411027
NEDD4	15q21.3	0.37112134	0.00059803	0.00411027
CPT1A	11q13.3	0.37107781	0.00059899	0.00411544
TENT4B	16q12.1	0.37103427	0.00059995	0.00412061

CELSR2	1p13.3	0.37101251	0.00060043	0.00412106
ZNF217	20q13.2	0.37099074	0.00060091	0.00412294
TRRAP	7q22.1	0.3709472	0.00060187	0.00412528
SYT11	1q22	0.3709472	0.00060187	0.00412528
OTULIN	5p15.2	0.3708819	0.00060332	0.00413235
DNAH5	5p15.2	0.37083837	0.00060428	0.00413754
KIAA2026	9p24.1	0.37079483	0.00060525	0.00414274
FGF7	15q21.2	0.370686	0.00060767	0.00415647
STMN2	8q21.13	0.37056346	0.00061041	0.00417091
B3GALT1	2q24.3	0.37053681	0.00061101	0.00417356
ZSWIM6	5q12.1	0.37044656	0.00061304	0.0041831
IGFBPL1	9p13.1	0.37034389	0.00061535	0.00419266
GORAB	1q24.2	0.37033772	0.00061549	0.00419266
ZC3H6	2q14.1	0.37007651	0.00062141	0.00423155
CIPC	14q24.3	0.37005474	0.00062191	0.00423348
SERAC1	6q25.3	0.36998944	0.0006234	0.00424072
TBCEL	11q23.3	0.36990237	0.00062539	0.00425136
FBXO48	2p13.3	0.3698153	0.00062739	0.00426058
NFE2L1	17q21.32	0.36977177	0.00062839	0.00426592
MIER3	5q11.2	0.36940173	0.00063695	0.00431378
OMD	9q22.31	0.36940173	0.00063695	0.00431378
RO60	1q31.2	0.36922759	0.00064102	0.0043369
SCAPER	15q24.3	0.36918405	0.00064204	0.00434113
TMED7-TICA	5q22.3	0.36918238	0.00064208	0.00434113
FER1L6	8q24.13	0.36915632	0.00064269	0.00434379
LOC729603	6q25.3	0.36911875	0.00064357	0.00434828
SMAD5	5q31.1	0.3685528	0.00065701	0.00443002
DCDC2B	1p35.2	0.36852852	0.00065759	0.00443244
MINAR1	15q25.1	0.36844397	0.00065962	0.00444462
MCFD2	2p21	0.36840043	0.00066067	0.00445018
CA5B	Xp22.2	0.36837866	0.00066119	0.0044522
DAB2	5p13.1	0.36833513	0.00066224	0.00445724
RGS7BP	5q12.3	0.3683291	0.00066239	0.00445724
CASD1	7q21.3	0.36829159	0.00066329	0.00446183
ZBTB33	Xq24	0.36813922	0.00066698	0.00448515
KIAA0319	6p22.3	0.36799974	0.00067038	0.00450494
ANK2	4q25-q26	0.36787802	0.00067336	0.00452342
GRHL2	8q22.3	0.36785625	0.00067389	0.00452548
KCNS2	8q22.2	0.36784306	0.00067421	0.00452601
CCDC50	3q28	0.36783448	0.00067443	0.00452601
SYNJ2BP	14q24.2	0.36774741	0.00067656	0.00453731
TNS3	7p12.3	0.36768211	0.00067817	0.00454504
CDC5L	6p21.1	0.36763858	0.00067925	0.00454918
C5ORF51	5p13.1	0.36755151	0.0006814	0.00456053
PDE10A	6q27	0.36750797	0.00068248	0.00456622
GIT2	12q24.11	0.367225	0.00068953	0.00460876
CRY1	12q23.3	0.3671597	0.00069117	0.00461815
PP2D1	3p24.3	0.36694264	0.00069664	0.00465311

CDK14	7q21.13	0.36689849	0.00069775	0.00465901
ZDHHC21	9p22.3	0.36685495	0.00069885	0.00466013
EOGT	3p14.1	0.36685495	0.00069885	0.00466013
GPR176	15q14-q15.1	0.36683319	0.00069941	0.00466224
KITLG	12q21.32	0.36674612	0.00070162	0.00467542
THAP5	7q31.1	0.36641961	0.00070997	0.00472317
SACS	13q12.12	0.366289	0.00071334	0.00474081
MIER1	1p31.3	0.36626724	0.0007139	0.00474297
ANKRD26	10p12.1	0.3662237	0.00071502	0.00474728
NRCAM	7q31.1	0.36589719	0.00072352	0.00478932
PCDH11X	Xq21.31	0.36581714	0.00072562	0.00480123
GPD2	2q24.1	0.36581012	0.0007258	0.00480123
SHISA6	17p12	0.36567438	0.00072937	0.00482324
RAB27A	15q21.3	0.36559245	0.00073153	0.00483432
TBC1D32	6q22.31	0.36559245	0.00073153	0.00483432
GTF3C3	2q33.1	0.36557068	0.00073211	0.00483492
CDKL1	14q21.3	0.36554892	0.00073268	0.00483712
GFM1	3q25.32	0.36546185	0.00073499	0.00485074
LONRF3	Xq24	0.36541831	0.00073615	0.00485676
CHST6	16q23.1	0.36535301	0.00073788	0.00486662
ZNF267	16p11.2	0.36530948	0.00073904	0.00487265
GALNT3	2q24.3	0.36522241	0.00074137	0.00488637
FLT1	13q12.3	0.36517887	0.00074254	0.00489243
GOPC	6q22.1	0.36515711	0.00074312	0.00489466
DDX60	4q32.3	0.36504827	0.00074604	0.00491066
SEMA6D	15q21.1	0.36504827	0.00074604	0.00491066
NAA50	3q13.31	0.36500473	0.00074721	0.00491513
LRBA	4q31.3	0.3648959	0.00075015	0.00493282
PTPRE	10q26.2	0.36480883	0.00075251	0.00494342
RFWD3	16q23.1	0.36480883	0.00075251	0.00494342
TUBGCP4	15q15.3	0.36463469	0.00075724	0.00496961
NUDT21	16q13	0.36459116	0.00075843	0.00497577
C2ORF69	2q33.1	0.36452585	0.00076022	0.00498584
MMUT	6p12.3	0.36417758	0.0007698	0.00504539
RBM41	Xq22.3	0.36411228	0.00077161	0.00505392
LHFPL2	5q14.1	0.36409051	0.00077222	0.00505455
SORL1	11q24.1	0.36409051	0.00077222	0.00505455
AQR	15q14	0.36393814	0.00077646	0.00507731
MYOF	10q23.33	0.3638293	0.0007795	0.00509051
RPRD1B	20q11.23	0.36380753	0.00078011	0.00509282
CLINT1	5q33.3	0.36372046	0.00078255	0.0051071
ENTPD1	10q24.1	0.36367693	0.00078378	0.00511341
IGF1R	15q26.3	0.3636334	0.000785	0.00511806
RDX	11q22.3	0.3636334	0.000785	0.00511806
SALL1	16q12.1	0.36350279	0.00078869	0.00513874
ZNF224	19q13.31	0.36345926	0.00078992	0.00514509
KANSL1L	2q34	0.36330689	0.00079425	0.0051699
COL6A3	2q37.3	0.36308921	0.00080047	0.0052053

C9ORF64	9q21.32	0.36298038	0.0008036	0.00522222
ZNF92	7q11.21	0.36298038	0.0008036	0.00522222
USP28	11q23.2	0.36287154	0.00080674	0.00524091
BCL2L2	14q11.2	0.36278447	0.00080926	0.00525556
RAD21	8q24.11	0.36267563	0.00081242	0.00527435
HPS3	3q24	0.3625668	0.00081559	0.00529119
SCN7A	2q24.3	0.36255922	0.00081581	0.00529119
ZMYM6	1p34.3	0.36252326	0.00081686	0.00529456
VWA7	6p21.33	0.36245796	0.00081877	0.00530521
AVL9	7p14.3	0.36239266	0.00082068	0.00531588
LRRC15	3q29	0.36228382	0.00082388	0.00533314
TOP2B	3p24.2	0.36217499	0.00082709	0.0053487
ETNK1	12p12.1	0.36215322	0.00082773	0.00534939
SREK1	5q12.3	0.36215322	0.00082773	0.00534939
FAM135B	8q24.23	0.36203922	0.00083111	0.00536948
C12ORF49	12q24.22	0.36195731	0.00083355	0.00538346
CDC40	6q21	0.36193555	0.00083419	0.0053859
TM9SF3	10q24.1	0.36191378	0.00083484	0.00538834
QPCT	2p22.2	0.36180494	0.00083809	0.00540581
TRIM37	17q22	0.36178318	0.00083874	0.00540826
MEF2C	5q14.3	0.36163081	0.00084332	0.00543422
PKDREJ	22q13.31	0.36160904	0.00084397	0.00543573
GNRHR	4q13.2	0.36160486	0.0008441	0.00543573
CLDN16	3q28	0.36156409	0.00084532	0.00544012
DIAPH3	13q21.2	0.36152197	0.00084659	0.00544654
FAM172A	5q15	0.36147843	0.00084791	0.00545324
KATNAL1	13q12.3	0.3613043	0.00085319	0.00548187
PTPRJ	11p11.2	0.36126076	0.00085451	0.00548861
NFE2L2	2q31.2	0.36119546	0.0008565	0.00549867
IMPG1	6q14.1	0.36119124	0.00085663	0.00549867
CNTNAP1	17q21.2	0.36104309	0.00086116	0.00552064
SGTB	5q12.3	0.36104309	0.00086116	0.00552064
ZNF710	15q26.1	0.36065128	0.00087325	0.00558914
NEMF	14q21.3	0.36062951	0.00087393	0.0055903
CTAGE1	18q11.2	0.36062737	0.00087399	0.0055903
MID1	Xp22.2	0.36060774	0.0008746	0.00559241
INPP4B	4q31.21	0.36056421	0.00087596	0.00559568
RUNX1	21q22.12	0.36049891	0.00087799	0.00560688
CD93	20p11.21	0.36041184	0.00088071	0.00562065
UBE3A	15q11.2	0.3603683	0.00088208	0.00562574
UNC13A	19p13.11	0.36034654	0.00088276	0.00562649
APPBP2	17q23.2	0.36032477	0.00088344	0.00562904
SLC10A7	4q31.22	0.36004179	0.00089236	0.00567861
ANGPTL1	1q25.2	0.35999826	0.00089374	0.00568376
INKA2	1p13.2	0.35971528	0.00090275	0.00573374
SOX6	11p15.2	0.35967175	0.00090415	0.0057353
CDH11	16q21	0.35962821	0.00090554	0.0057405
SUCO	1q24.3	0.35945408	0.00091115	0.00576868

PFN1P2	1p11.2	0.35938877	0.00091326	0.0057802
CDK15	2q33.1	0.35924041	0.00091807	0.00580879
LINC01881	2q37.3	0.35919287	0.00091961	0.00581672
CD46	1q32.2	0.3591058	0.00092245	0.00582912
CASS4	20q13.31	0.35875752	0.00093388	0.00589386
LUZP6	7q33	0.35875752	0.00093388	0.00589386
STAT3	17q21.2	0.35873576	0.0009346	0.00589653
RECK	9p13.3	0.35871399	0.00093532	0.00589733
SEC31A	4q21.22	0.35864869	0.00093748	0.00590908
DPPA2P3	3q29	0.35853551	0.00094123	0.00593077
HTR2A	13q14.2	0.35852047	0.00094173	0.00593077
PRPF8	17p13.3	0.35838748	0.00094617	0.00595253
ITGB8	7p21.1	0.35825688	0.00095054	0.00597814
NR2F2	15q26.2	0.35823511	0.00095127	0.00598085
KIF3A	5q31.1	0.35816981	0.00095346	0.00599275
LIMK2	22q12.2	0.35814804	0.00095419	0.00599546
SUCNR1	3q25.1	0.35801938	0.00095853	0.00601702
AARS	16q22.1	0.35769093	0.00096969	0.00607558
ITGA2	5q11.2	0.35753856	0.0009749	0.00610634
PCDHGA6	5q31.3	0.35749502	0.0009764	0.00611378
SPRED1	15q14	0.35732088	0.0009824	0.00614556
ATMIN	16q23.2	0.35727735	0.00098391	0.00615304
MFAP3L	4q33	0.35725558	0.00098466	0.00615582
AGPS	2q31.2	0.35716851	0.00098768	0.00617276
MASP2	1p36.22	0.35714674	0.00098843	0.00617555
STAG3L1	7q11.23	0.35695084	0.00099526	0.00621431
CSNK1A1L	13q13.3	0.35692902	0.00099603	0.00621713
FCHO2	5q13.2	0.356842	0.00099908	0.00623421
BRD4	19p13.12	0.3566461	0.00100597	0.00626938
NPC1	18q11.2	0.35660256	0.00100751	0.006277
ALG10	12p11.1	0.35655903	0.00100905	0.0062807
PLEKHA3	2q31.2	0.35645019	0.00101291	0.00630079
LRCH1	13q14.13-q1	0.35625429	0.00101989	0.00633829
GAPVD1	9q33.3	0.35621075	0.00102145	0.00634599
SMARCA5	4q31.21	0.35608015	0.00102613	0.00637112
RHOBTB3	5q15	0.35601485	0.00102848	0.00638173
IL17REL	22q13.33	0.3559925	0.00102929	0.00638474
TNFRSF9	1p36.23	0.35577541	0.00103714	0.00642945
NT5E	6q14.3	0.35575364	0.00103793	0.00643235
SOS2	14q21.3	0.35560127	0.00104348	0.00646472
TMEM181	6q25.3	0.35540536	0.00105066	0.00650309
ANGEL1	14q24.3	0.35536183	0.00105226	0.00650693
PDE4D	5q11.2-q12.1	0.35536183	0.00105226	0.00650693
ARRDC3	5q14.3	0.35520946	0.00105787	0.00653964
SLC26A2	5q32	0.35510062	0.0010619	0.00655844
RUNX2	6p21.1	0.35503532	0.00106433	0.0065673
GLUD2	Xq24	0.35492648	0.00106838	0.00659026
TPR	1q31.1	0.35488295	0.00107	0.00659824

CEP126	11q22.1	0.35477411	0.00107407	0.00661925
SH3D19	4q31.3	0.35468704	0.00107734	0.00663528
GPR141	7p14.1	0.35464544	0.00107891	0.00664286
CDH13	16q23.3	0.3545782	0.00108144	0.00665433
CNKSR2	Xp22.12	0.35456416	0.00108197	0.00665554
ENPP1	6q23.2	0.35446937	0.00108555	0.0066755
SLAIN2	4p11	0.35433876	0.0010905	0.00670181
MRE11	11q21	0.35427346	0.00109298	0.00671293
MYLK	3q21.1	0.35416463	0.00109713	0.00672805
ZNF28	19q13.41	0.35416463	0.00109713	0.00672805
TRUB1	10q25.3	0.35414286	0.00109797	0.00673108
HSPA13	21q11.2	0.35401225	0.00110297	0.00675967
FLRT2	14q31.3	0.35399049	0.0011038	0.00676271
AP2B1	17q12	0.35359868	0.00111895	0.00684496
TRAM1	8q13.3	0.35305449	0.00114029	0.00695844
EFEMP1	2p16.1	0.35301096	0.00114202	0.00696469
YTHDC2	5q22.2	0.35296743	0.00114374	0.00697308
ATP10D	4p12	0.35279329	0.00115067	0.00701102
C5ORF22	5p13.3	0.35272799	0.00115328	0.00702046
PPP4R3A	14q32.12	0.35259738	0.00115851	0.00705015
FAM106C	17p11.2	0.35252304	0.00116149	0.00706617
NRIP3	11p15.4	0.35233617	0.00116903	0.00710769
AMMECR1L	2q14.3	0.35209673	0.00117876	0.00716026
DHX9	1q25.3	0.35207497	0.00117965	0.00716116
BMP8A	1p34.3	0.3520532	0.00118053	0.00716116
ZNF765	19q13.42	0.3520532	0.00118053	0.00716116
SLC16A6	17q24.2	0.35203143	0.00118142	0.00716116
NXPE3	3q12.3	0.35190083	0.00118677	0.00719138
ATAD5	17q11.2	0.35183553	0.00118945	0.00720544
CASC4	15q15.3	0.35166139	0.00119663	0.00724451
VSIG10	12q24.23	0.35161785	0.00119843	0.007251
HACE1	6q16.3	0.35159609	0.00119933	0.00725205
KCTD7	7q11.21	0.35157432	0.00120023	0.0072553
CCDC36	3p21.31	0.35152181	0.00120241	0.00726626
PCGF5	10q23.32	0.35144372	0.00120565	0.00728366
ADAMTS2	5q35.3	0.35126958	0.00121292	0.00731868
CHRNA6	8p11.21	0.3511289	0.00121882	0.00734759
RCOR1	14q32.31-q3	0.35109544	0.00122023	0.00735162
ZNF808	19q13.41	0.35107367	0.00122114	0.00735269
HCG18	6p22.1	0.3510519	0.00122206	0.00735599
XKR4	8q12.1	0.35082648	0.00123159	0.0074044
OXSR1	3p22.2	0.3507907	0.00123311	0.00740906
GXYLT1	12q12	0.3507907	0.00123311	0.00740906
TXNRD1	12q23.3	0.35048595	0.00124611	0.00747366
PPARGC1A	4p15.2	0.35042065	0.00124891	0.00748596
SIX4	14q23.1	0.35039889	0.00124985	0.00748706
NAA35	9q21.33	0.35039889	0.00124985	0.00748706
TTPAL	20q13.12	0.35020298	0.0012583	0.00753314

NLRP14	11p15.4	0.35009538	0.00126296	0.00755794
KLHL2	4q32.3	0.35007238	0.00126396	0.00755794
LYRM7	5q23.3-q31.1	0.35007238	0.00126396	0.00755794
WDHD1	14q22.2-q22	0.35007238	0.00126396	0.00755794
NRIP1	21q11.2-q21	0.35000707	0.0012668	0.00757264
MED20	6p21.1	0.34989824	0.00127154	0.00759872
RPL23AP53	8p23.3	0.34981117	0.00127535	0.00761234
SYNGAP1	6p21.32	0.34963703	0.00128299	0.0076488
RBAK	7p22.1	0.34957173	0.00128587	0.00765908
POLA1	Xp22.11-p21	0.34944112	0.00129164	0.00768427
PPP1R9A	7q21.3	0.34941936	0.00129261	0.00768771
IGF1	12q23.2	0.34939759	0.00129357	0.00769116
FAM8A1	6p22.3	0.34922345	0.00130132	0.00773258
PDGFD	11q22.3	0.34904931	0.0013091	0.00777421
SLC30A9	4p13	0.34891871	0.00131497	0.00780672
SCAF8	6q25.2	0.34885341	0.00131791	0.00781953
MITF	3p13	0.34883164	0.00131889	0.00782303
DDHD1	14q22.1	0.34880987	0.00131988	0.00782653
MYBL1	8q13.1	0.34876634	0.00132184	0.00783586
SEPTIN8	5q31.1	0.3487228	0.00132381	0.00784054
PCDHGC5	5q31.3	0.34867927	0.00132579	0.00784756
WNK3	Xp11.22	0.34867927	0.00132579	0.00784756
ARGLU1	13q33.3	0.34861397	0.00132875	0.0078581
ICE2	15q22.2	0.3485922	0.00132974	0.00786162
CCDC80	3q13.2	0.34857043	0.00133073	0.00786514
TAOK3	12q24.23	0.34854867	0.00133172	0.00786632
TSNAX	1q42.2	0.34854867	0.00133172	0.00786632
PATL1	11q12.1	0.34833099	0.00134167	0.00790631
FYCO1	3p21.31	0.34833099	0.00134167	0.00790631
ZNF264	19q13.43	0.34826569	0.00134466	0.00791928
NRAS	1p13.2	0.34820039	0.00134767	0.00793462
KCTD12	13q22.3	0.34809155	0.00135268	0.00795945
PLCL1	2q33.1	0.34802625	0.0013557	0.00797486
GRIP1	12q14.3	0.34800448	0.00135671	0.00797608
DCHS1	11p15.4	0.34798272	0.00135772	0.00797965
ZRANB3	2q21.3	0.34793918	0.00135974	0.0079868
ZNRF3	22q12.1	0.34791741	0.00136075	0.00798802
SMIM14	4p14	0.34791741	0.00136075	0.00798802
GDNF	5p13.2	0.3478262	0.00136499	0.00801056
SFT2D2	1q24.2	0.34759091	0.00137598	0.00806558
TMEM64	8q21.3	0.34750384	0.00138007	0.00808717
ECT2	3q26.31	0.34748207	0.0013811	0.00809079
DPY19L1	7p14.2	0.34743853	0.00138315	0.00809803
PDE5A	4q26	0.34737323	0.00138623	0.0081113
RASSF2	20p13	0.3473297	0.00138828	0.00812095
GVINP1	11p15.4	0.34695965	0.00140588	0.00821423
GXYLT2	3p13	0.34695965	0.00140588	0.00821423
SAMD12	8q24.11-q24	0.34685082	0.00141109	0.00823985

FBXW11	5q35.1	0.34676375	0.00141528	0.00826186
CDC14A	1p21.2	0.34667668	0.00141947	0.00827664
EPS15	1p32.3	0.34661138	0.00142262	0.00829017
FAM76A	1p35.3	0.34645901	0.00143001	0.00832102
NEK9	14q24.3	0.34621957	0.00144168	0.00838403
ADAM28	8p21.2	0.3461978	0.00144274	0.00838778
RPS15AP10	1p34.1	0.34607996	0.00144852	0.00841892
CYP27C1	2q14.3	0.34602366	0.00145129	0.00843255
CP	3q24-q25.1	0.34595836	0.00145451	0.00844878
KIAA0513	16q24.1	0.34589306	0.00145773	0.0084601
ZNF543	19q13.43	0.34589306	0.00145773	0.0084601
PLPP3	1p32.2	0.34578422	0.00146312	0.00848395
ACTR3	2q14.1	0.34576245	0.0014642	0.00848774
PER3	1p36.23	0.34571892	0.00146636	0.00849779
TMEM123	11q22.2	0.34526181	0.00148923	0.00862028
XCR1	3p21.31	0.34495243	0.00150488	0.00870586
PIWIL2	8p21.3	0.3449323	0.00150591	0.00870926
ZC3H12C	11q22.3	0.34478293	0.00151353	0.0087457
FAM76B	11q21	0.34460879	0.00152245	0.00878453
OR2C1	16p13.3	0.34435164	0.00153572	0.00885339
A4GNT	3q22.3	0.344333	0.00153669	0.0088564
AHNAK2	14q32.33	0.34426051	0.00154045	0.00887294
ZZZ3	1p31.1	0.34426051	0.00154045	0.00887294
KBTBD4	11p11.2	0.34421698	0.00154271	0.0088834
USP44	12q22	0.34407209	0.00155026	0.00891883
ZC3H11A	1q32.1	0.34406461	0.00155065	0.00891883
ANKRD52	12q13.3	0.34397754	0.00155521	0.00893986
ARPIN	15q26.1	0.3438034	0.00156436	0.00898726
PER2	2q37.3	0.34371633	0.00156895	0.00901104
USP32	17q23.1-q23	0.34369456	0.0015701	0.00901504
ILRUN	6p21.31	0.34360749	0.0015747	0.00903629
CDR1	Xq27.1	0.34360749	0.0015747	0.00903629
SPG11	15q21.1	0.34319391	0.00159675	0.00914964
UBE2Q2P1	15q25.2	0.34310684	0.00160143	0.00917381
EIF5	14q32.32	0.34308508	0.0016026	0.00917524
TMCO3	13q34	0.34308508	0.0016026	0.00917524
ZW10	11q23.2	0.34291094	0.001612	0.0092211
PURB	7p13	0.34288917	0.00161318	0.00922519
DHX15	4p15.2	0.3428021	0.0016179	0.00924953
CDV3	3q22.1	0.34278034	0.00161908	0.00925363
ALMS1	2p13.1	0.3427368	0.00162145	0.0092645
TCF19	6p21.33	0.3426715	0.00162501	0.00928216
AHCYL2	7q32.1	0.3426062	0.00162857	0.00929451
EHF	11p13	0.3426062	0.00162857	0.00929451
CMTM4	16q21-q22.1	0.34258443	0.00162976	0.00929597
LCORL	4p15.31	0.34258443	0.00162976	0.00929597
DSG2	18q12.1	0.34212732	0.00165491	0.00943406
HARBI1	11p11.2	0.34195318	0.00166459	0.00948107

FBXL4	6q16.1-q16.2	0.34190964	0.00166702	0.00949218
CXXC4	4q24	0.3418877	0.00166824	0.00949643
BMP8B	1p34.2	0.34184434	0.00167066	0.0095075
THAP2	12q21.1	0.34182257	0.00167188	0.00951171
TAGAP	6q25.3	0.34177904	0.00167432	0.00952285
CAPS2	12q21.1-q21	0.34173551	0.00167676	0.009534
SLC35D1	1p31.3	0.34171374	0.00167798	0.00953822
PARM1	4q13.3	0.34169197	0.0016792	0.00954244
CPEB3	10q23.32	0.34143076	0.00169392	0.00962063
SWAP70	11p15.4	0.341409	0.00169515	0.00962488
ZDBF2	2q33.3	0.34134369	0.00169886	0.00964315
PTPN4	2q14.2	0.34127839	0.00170256	0.00966145
CEP295	11q21	0.34121309	0.00170628	0.00967978
PRICKLE2	3p14.1	0.34119132	0.00170752	0.00968406
DISC1	1q42.2	0.34116956	0.00170876	0.00968558
TMEM263	12q23.3	0.34099542	0.00171872	0.00973372
AP5M1	14q22.3	0.34093012	0.00172247	0.00975217
NHS	Xp22.2-p22.1	0.34090835	0.00172372	0.00975648
SEPTIN11	4q21.1	0.34084305	0.00172748	0.00976887
HRH4	18q11.2	0.3408362	0.00172787	0.00976887
USP1	1p31.3	0.34082128	0.00172873	0.00977096
ST6GAL2	2q12.3	0.34079951	0.00172999	0.00977528
PDCD6IP	3p22.3	0.34075598	0.0017325	0.00978393
IPO11	5q12.1	0.34075598	0.0017325	0.00978393
ATAD2	8q24.13	0.34071244	0.00173502	0.00978981
HDAC4	2q37.3	0.34071244	0.00173502	0.00978981
SH3RF1	4q32.3-q33	0.34071244	0.00173502	0.00978981
BCL9L	11q23.3	0.34066891	0.00173753	0.0097957
RBM43	2q23.3	0.34062537	0.00174006	0.00980714
BTC	4q13.3	0.34060361	0.00174132	0.00981148
NUP160	11p11.2	0.34045124	0.00175018	0.00985583
MARS2	2q33.1	0.34036417	0.00175526	0.00987328
SEPTIN2	2q37.3	0.34019003	0.00176546	0.00992506
ABI3BP	3q12.2	0.33990705	0.00178216	0.00999916
MYNN	3q26.2	0.33981998	0.00178732	0.01002249
KCNJ9	1q23.2	0.33969925	0.00179451	0.01005994
FOXO1	13q14.11	0.33962408	0.00179899	0.01008226
SEMA3D	7q21.11	0.33942817	0.00181073	0.01013949
SNX13	7p21.1	0.33942817	0.00181073	0.01013949
KCTD16	5q31.3	0.33937804	0.00181375	0.01015007
TIRAP	11q24.2	0.33936287	0.00181466	0.01015007
TRAK1	3p22.1	0.33936287	0.00181466	0.01015007
KRIT1	7q21.2	0.33936287	0.00181466	0.01015007
PRKCI	3q26.2	0.33923227	0.00182254	0.01018557
RCOR3	1q32.2-q32.3	0.3392105	0.00182386	0.01019006
VIRMA	8q22.1	0.33918873	0.00182517	0.01019456
KIAA0100	17q11.2	0.33912343	0.00182913	0.0102138
FARP2	2q37.3	0.33905813	0.00183309	0.01023307

FAM83B	6p12.1	0.33901459	0.00183574	0.01024497
CHRM4	11p11.2	0.33887919	0.001844	0.0102824
GNA11	19p13.3	0.33884046	0.00184637	0.01029272
HGSNAT	8p11.21-p11	0.33870985	0.00185437	0.0103229
TXNDC16	14q22.1	0.33862278	0.00185972	0.01034691
GCNT2	6p24.3-p24.2	0.33844865	0.00187047	0.0103893
ZNF774	15q26.1	0.33840511	0.00187317	0.01039558
PCDHGB1	5q31.3	0.33836158	0.00187587	0.01040766
ZNF134	19q13.43	0.33810037	0.00189214	0.01048624
CLIP4	2p23.2	0.3380786	0.0018935	0.01049087
SMURF2	17q23.3-q24	0.33805683	0.00189486	0.01049258
ZNF112	19q13.31	0.33803507	0.00189622	0.01049721
SLC38A7	16q21	0.33792623	0.00190305	0.01053209
ZNF557	19p13.2	0.33790446	0.00190442	0.01053381
TIMP3	22q12.3	0.3378827	0.00190579	0.01053846
MAN2A2	15q26.1	0.33786093	0.00190716	0.01054311
KIAA1147	7q34	0.33781739	0.0019099	0.01055242
ANKRD28	3p25.1	0.33768679	0.00191816	0.01058626
LOX	5q23.1	0.33757795	0.00192506	0.01062141
PATJ	1p31.3	0.33755619	0.00192644	0.01062315
CEMIP	15q25.1	0.33751265	0.00192921	0.01063547
FPGT	1p31.1	0.33744735	0.00193337	0.01065251
ARL5A	2q23.3	0.33738205	0.00193753	0.01066662
NRXN3	14q24.3-q31	0.33738205	0.00193753	0.01066662
CCDC144CP	17p11.2	0.33730139	0.00194269	0.01068911
GMEB1	1p35.3	0.33705554	0.00195849	0.01075524
PLXNA4	7q32.3	0.337012	0.0019613	0.0107677
CNST	1q44	0.33690317	0.00196834	0.01079922
ZNF664	12q24.31	0.3368814	0.00196975	0.01079922
SMC2	9q31.1	0.33685963	0.00197117	0.01080399
LRP6	12p13.2	0.33683787	0.00197258	0.01080875
CSPP1	8q13.1-q13.2	0.3368161	0.00197399	0.01081352
PDE4DIP	1q21.2	0.33664196	0.00198533	0.01086967
ARHGEF7	13q34	0.33640252	0.00200102	0.01094354
PREPL	2p21	0.33631545	0.00200676	0.01096887
ZNF713	7p11.2	0.33625015	0.00201107	0.0109864
EML4	2p21	0.33622838	0.0020125	0.01099124
CYSLTR2	13q14.2	0.33606696	0.0020232	0.0110436
CMAHP	6p22.3	0.33601071	0.00202694	0.01106098
SH3PXD2A	10q24.33	0.33590187	0.00203419	0.01109447
ARHGAP12	10p11.22	0.3357495	0.00204438	0.01114396
MFAP5	12p13.31	0.3356842	0.00204877	0.01116479
ZNF564	19p13.2	0.33551006	0.00206049	0.01122563
RP2	Xp11.3	0.33548829	0.00206196	0.0112275
KPNA3	13q14.2	0.33546653	0.00206343	0.01123244
PRUNE2	9q21.2	0.33522709	0.00207968	0.01131468
FCAR	19q13.42	0.33517687	0.0020831	0.01132092
DCAF7	17q23.3	0.33511825	0.0020871	0.01133957

NFKB1	4q24	0.33509648	0.00208859	0.01134456
ABCA8	17q24.2	0.33505477	0.00209144	0.01135695
TSPY26P	20q11.21	0.33496588	0.00209753	0.01138072
HECTD2	10q23.32	0.33494411	0.00209902	0.01138572
PTMAP11	9q22.33	0.33481422	0.00210796	0.01142485
RSRC1	3q25.32	0.33466114	0.00211853	0.01147591
GPR31	6q27	0.33464106	0.00211992	0.01147784
GATAD2B	1q21.3	0.33463937	0.00212004	0.01147784
CRISPLD2	16q24.1	0.33457407	0.00212457	0.0114961
RANBP6	9p24.1	0.33446523	0.00213213	0.01153077
ADAMTS16	5p15.32	0.3343038	0.0021434	0.01158541
PPFIA1	11q13.3	0.33424756	0.00214734	0.01160355
KCTD18	2q33.1	0.33413872	0.00215498	0.01163851
ARHGEF11	1q23.1	0.33394282	0.00216879	0.01170675
SLC38A1	12q13.11	0.33392105	0.00217033	0.01171189
RSPH3	6q25.3	0.33389928	0.00217187	0.01171703
TAS2R3	7q34	0.33385691	0.00217487	0.01172414
GALC	14q31.3	0.33385575	0.00217495	0.01172414
ZNF407	18q22.3	0.33385575	0.00217495	0.01172414
TBX18	6q14.3	0.33379045	0.00217958	0.01173323
SLC1A4	2p14	0.33374691	0.00218268	0.01174354
SETDB2	13q14.2	0.33374691	0.00218268	0.01174354
YOD1	1q32.1	0.33368161	0.00218733	0.01176537
MATR3	5q31.2	0.33346394	0.00220288	0.01183307
CHMP2B	3p11.2	0.33344217	0.00220445	0.01183827
TLR4	9q33.1	0.33339864	0.00220757	0.01185186
ATXN2	12q24.12	0.33331157	0.00221383	0.01187908
SPPL2A	15q21.2	0.33331157	0.00221383	0.01187908
RHOJ	14q23.2	0.3331592	0.00222483	0.01192524
ARHGEF6	Xq26.3	0.33313743	0.00222641	0.01193047
LRP12	8q22.3	0.33298506	0.00223746	0.01198649
DDR1	6p21.33	0.33294152	0.00224063	0.01200023
CASC3	17q21.1	0.33285445	0.00224698	0.01202775
LINC01000	7q32.1	0.33272385	0.00225653	0.01207239
XPR1	1q25.3	0.33272385	0.00225653	0.01207239
LCLAT1	2p23.1	0.33270208	0.00225812	0.01207768
ATXN10	22q13.31	0.33265855	0.00226132	0.01208827
MID2	Xq22.3	0.33265855	0.00226132	0.01208827
KLHL5	4p14	0.33250618	0.00227253	0.01213842
PHACTR1	6p24.1	0.33244087	0.00227735	0.0121609
CANX	5q35.3	0.33239734	0.00228056	0.01217482
OSTM1	6q21	0.33231027	0.00228701	0.01220271
STK38	6p21.31	0.3322885	0.00228863	0.01220805
SYNPO2	4q26	0.33217967	0.00229672	0.01224464
ZNF493	19p12	0.3321579	0.00229834	0.01225
PPM1B	2p21	0.3320273	0.00230808	0.01229866
MBTD1	17q21.33	0.33196199	0.00231297	0.01231811
TRIM2	4q31.3	0.33194023	0.0023146	0.01232351

CALD1	7q33	0.33178786	0.00232605	0.01237784
PPARGC1B	5q32	0.33174432	0.00232933	0.01239198
SPIN4	Xq11.1	0.33170079	0.00233261	0.01240282
SIRPB1	20p13	0.33167902	0.00233426	0.01240825
CNTN1	12q12	0.33159195	0.00234084	0.0124333
PAQR8	6p12.2	0.33148311	0.0023491	0.01246385
PDXDC1	16p13.11	0.33146135	0.00235075	0.01246598
AGO2	8q24.3	0.33146135	0.00235075	0.01246598
RPL7L1	6p21.1	0.33143958	0.0023524	0.01247144
KTN1	14q22.3	0.33141781	0.00235406	0.0124769
VPS50	7q21.2-q21.3	0.33126544	0.00236568	0.01253516
FMR1	Xq27.3	0.33120014	0.00237068	0.01254829
PDGFRA	4q12	0.33120014	0.00237068	0.01254829
ARHGAP35	19q13.32	0.33113484	0.00237569	0.0125681
EFCAB13	17q21.32	0.33093893	0.00239076	0.01264114
RPGRIP1L	16q12.2	0.33085186	0.00239749	0.01266663
TCP11L1	11p13	0.3308301	0.00239917	0.01267217
PCDH1	5q31.3	0.33080833	0.00240086	0.01267771
KIAA1958	9q32	0.33078656	0.00240255	0.01267989
ZNF652	17q21.32-q2	0.33065596	0.00241269	0.01272331
RUBCN	3q29	0.33063419	0.00241438	0.01272887
HIF1A	14q23.2	0.33059066	0.00241777	0.01274001
NUMB	14q24.2-q24	0.33059066	0.00241777	0.01274001
IL1RAP	3q28	0.33043828	0.00242968	0.01279597
ITPRIPL2	16p12.3	0.33039475	0.00243309	0.01280716
SMYD4	17p13.3	0.33039475	0.00243309	0.01280716
NOS2	17q11.2	0.33037298	0.0024348	0.01281276
IFNAR2	21q22.11	0.33035122	0.00243651	0.01281836
SLC20A2	8p11.21	0.33022061	0.00244678	0.0128656
PCDHGB8P	5q31.3	0.33018555	0.00244954	0.01287673
ADAMTS5	21q21.3	0.33015531	0.00245193	0.01288248
DTWD2	5q23.1	0.33004647	0.00246053	0.01291066
FUBP3	9q34.11-q34	0.33000294	0.00246398	0.01292536
LOC148696	1q32.2	0.32995697	0.00246763	0.01294045
SEPHS1P1	7q11.21	0.32995032	0.00246816	0.01294045
ANKHD1	5q31.3	0.32993764	0.00246916	0.01294232
ZNF322	6p22.2	0.32991587	0.00247089	0.01294798
IL23R	1p31.3	0.32988716	0.00247318	0.01295653
PIK3R4	3q22.1	0.32980703	0.00247956	0.01298655
MDFIC	7q31.1-q31.2	0.32974173	0.00248477	0.01300018
TSPO2	6p21.1	0.32956017	0.00249931	0.01306597
LINC01591	8q24.22	0.32946261	0.00250716	0.01310355
GPR32	19q13.33	0.32937653	0.0025141	0.01313293
OTUD3	1p36.13	0.32934992	0.00251625	0.01313727
KCNA3	1p13.3	0.32934992	0.00251625	0.01313727
TRIM66	11p15.4	0.32932815	0.00251801	0.01314301
ERLEC1	2p16.2	0.32930639	0.00251977	0.01314531
KCNG3	2p21	0.32928077	0.00252184	0.01315268

BVES	6q21	0.32926285	0.00252329	0.0131568
SGCD	5q33.2-q33.3	0.32921932	0.00252682	0.01317175
OR5K2	3q11.2	0.32895955	0.00254797	0.01327157
JRKL	11q21	0.32878397	0.00256235	0.01333602
LAMA4	6q21	0.32878397	0.00256235	0.01333602
TSC1	9q34.13	0.32843569	0.0025911	0.01347859
LRRFIP1	2q37.3	0.32837039	0.00259652	0.01350327
AGTPBP1	9q21.33	0.32832686	0.00260014	0.01351857
POM121	7q11.23	0.32819625	0.00261103	0.01356809
MICALCL	11p15.3	0.32817449	0.00261285	0.013574
TRIQK	8q22.1	0.32815272	0.00261467	0.01357991
MED12L	3q25.1	0.32804388	0.00262378	0.01362016
CKAP2	13q14.3	0.32802212	0.00262561	0.01362254
CDK8	13q12.13	0.32793505	0.00263293	0.01365695
CENPC	4q13.2	0.32789151	0.00263659	0.0136724
CCNYL1	2q33.3	0.32786974	0.00263843	0.01367835
BIRC3	11q22.2	0.32754324	0.00266609	0.01380018
RNPC3	1p21.1	0.32752147	0.00266794	0.01380619
FAM120B	6q27	0.3274344	0.00267536	0.01383741
ANGEL2	1q32.3	0.3273691	0.00268094	0.01386267
POM121C	7q11.23	0.32734733	0.0026828	0.0138687
LIN52	14q24.3	0.32732556	0.00268467	0.01387473
KLF5	13q22.1	0.32712966	0.00270149	0.01394604
RLF	1p34.2	0.32710789	0.00270337	0.01394604
CHUK	10q24.31	0.32710789	0.00270337	0.01394604
SYNRG	17q12	0.32710789	0.00270337	0.01394604
KLHL4	Xq21.31	0.32709324	0.00270463	0.01394894
TOPBP1	3q22.1	0.32704259	0.002709	0.01396786
SIGLEC6	19q13.41	0.32695537	0.00271654	0.01399948
ATXN7L1	7q22.3	0.32689022	0.00272218	0.01401044
OPA1	3q29	0.32689022	0.00272218	0.01401044
ZSCAN29	15q15.3	0.32689022	0.00272218	0.01401044
SHC4	15q21.1	0.32675961	0.00273353	0.0140543
MECOM	3q26.2	0.32671608	0.00273732	0.01406653
DSTYK	1q32.1	0.32669431	0.00273921	0.01407265
GPR157	1p36.22	0.32667254	0.00274111	0.01407878
DOK6	18q22.2	0.32658547	0.00274872	0.01410329
NIBAN1	1q25.3	0.32654194	0.00275253	0.01411557
SRPK2	7q22.3	0.32649841	0.00275634	0.01412422
CEP170P1	4q26	0.32622253	0.00278063	0.01423173
MIA3	1q41	0.32621543	0.00278126	0.01423173
FOXP2	7q31.1	0.32621142	0.00278161	0.01423173
KIAA0825	5q15	0.32619366	0.00278318	0.0142361
MELTF	3q29	0.3261719	0.00278511	0.01423864
RNF219	13q31.1	0.3261719	0.00278511	0.01423864
EYS	6q12	0.32615013	0.00278703	0.01424483
AFTPH	2p14	0.32612836	0.00278896	0.01425103
BCL2L11	2q13	0.32599776	0.00280055	0.01430294

TFEC	7q31.2	0.32599776	0.00280055	0.01430294
TMEM67	8q22.1	0.32588892	0.00281025	0.01434509
ARRDC4	15q26.2	0.32575832	0.00282192	0.01440099
ZNF532	18q21.32	0.32558418	0.00283755	0.01446964
TBC1D4	13q22.2	0.32534474	0.00285918	0.01456498
EIF4G3	1p36.12	0.32519237	0.00287301	0.01462797
VHL	3p25.3	0.32514883	0.00287697	0.01464441
UVSSA	4p16.3	0.32493116	0.00289687	0.01472685
RPRD2	1q21.2	0.32493116	0.00289687	0.01472685
FAM102B	1p13.3	0.32486586	0.00290286	0.01475355
LINC00312	3p25.3	0.32484409	0.00290486	0.01475618
PIGW	17q12	0.32484409	0.00290486	0.01475618
SLC9A8	20q13.13	0.32477879	0.00291087	0.01477539
TBC1D22B	6p21.2	0.32477879	0.00291087	0.01477539
HPSE	4q21.23	0.32475702	0.00291287	0.01478068
LDHAL6A	11p15.1	0.32473894	0.00291454	0.01478068
PLS1	3q23	0.32473526	0.00291488	0.01478068
TECPR2	14q32.31	0.32471349	0.00291688	0.01478333
UNC5B	10q22.1	0.32471349	0.00291688	0.01478333
BCL6	3q27.3	0.32462642	0.00292493	0.01481544
NAA16	13q14.11	0.32460465	0.00292694	0.01481544
STOX2	4q35.1	0.32460465	0.00292694	0.01481544
USP53	4q26	0.32456112	0.00293097	0.01482454
KBTBD8	3p14.1	0.32451758	0.002935	0.01484119
HMGCR	5q13.3	0.32445228	0.00294107	0.01485675
ST8SIA6	10p12.33	0.32433433	0.00295205	0.01490683
ZMPSTE24	1p34.2	0.32432168	0.00295323	0.01490683
SIKE1	1p13.2	0.32432168	0.00295323	0.01490683
SPIC	12q23.2	0.32429443	0.00295577	0.01491588
PLCE1	10q23.33	0.32425638	0.00295932	0.01492626
FZD4	11q14.2	0.32412577	0.00297155	0.01498413
CEP85	1p36.11	0.3239081	0.00299203	0.0150645
TNFRSF11B	8q24.12	0.32360336	0.00302091	0.01518688
IKZF4	12q13.2	0.32360336	0.00302091	0.01518688
OSMR	5p13.1	0.32358159	0.00302298	0.01518963
SMC1B	22q13.31	0.32358159	0.00302298	0.01518963
HIST3H3	1q42.13	0.323478	0.00303286	0.01523543
FN1	2q35	0.32345099	0.00303544	0.01524455
SMARCA2	9p24.3	0.32340745	0.0030396	0.01526162
PTCH1	9q22.32	0.32334215	0.00304586	0.01527762
ZNF423	16q12.1	0.32332038	0.00304795	0.01528425
CHL1	3p26.3	0.32323331	0.00305631	0.01531847
EPB41L3	18p11.31	0.32318978	0.0030605	0.01533176
CKAP2L	2q14.1	0.32316801	0.0030626	0.01533455
FBN2	5q23.3	0.32314624	0.0030647	0.01534119
LEMD1	1q32.1	0.32297914	0.00308084	0.01540655
OR13J1	9p13.3	0.32295313	0.00308337	0.01541528
NCSTN	1q23.2	0.32279797	0.00309844	0.01547898

KNTC1	12q24.31	0.32275443	0.00310268	0.01549628
NCK1	3q22.3	0.32273267	0.0031048	0.015503
ERCC6	10q11.23	0.32268913	0.00310905	0.01551643
IMPG2	3q12.3	0.32249322	0.00312824	0.01560827
APBB2	4p14-p13	0.32244969	0.00313251	0.01561788
TRDMT1	10p13	0.32244969	0.00313251	0.01561788
WAC	10p12.1|10p	0.32236262	0.00314109	0.01564886
OPHN1	Xq12	0.32231909	0.00314538	0.01566137
TRAPPC10	21q22.3	0.32229732	0.00314753	0.01566137
ACTR2	2p14	0.32229732	0.00314753	0.01566137
PLCB4	20p12.3-p12	0.32223202	0.00315398	0.01568956
XIST	Xq13.2	0.32205788	0.00317125	0.01576758
EPB41L2	6q23.1-q23.2	0.32199258	0.00317775	0.01579594
MOXD1	6q23.2	0.32179667	0.00319731	0.01588526
B3GNT2	2p15	0.32179667	0.00319731	0.01588526
NMD3	3q26.1	0.3217096	0.00320604	0.01592069
COL12A1	6q13-q14.1	0.32166607	0.00321041	0.01593843
ASPA	17p13.2	0.3216281	0.00321423	0.01594824
PALLD	4q32.3	0.321579	0.00321917	0.01596601
AP1S2	Xp22.2	0.3215137	0.00322576	0.0159907
AFF1	4q21.3-q22.1	0.32140486	0.00323676	0.01603726
YME1L1	10p12.1	0.32140486	0.00323676	0.01603726
ADAM12	10q26.2	0.32136133	0.00324117	0.01605112
NEDD9	6p24.2	0.32136133	0.00324117	0.01605112
CMIP	16q23.2-q23	0.32133956	0.00324338	0.01605806
FANCI	15q26.1	0.32127426	0.00325001	0.01608688
GALNT13	2q23.3-q24.1	0.32113452	0.00326423	0.01614526
RYR2	1q43	0.32112189	0.00326552	0.01614763
FYTTD1	3q29	0.32105658	0.00327219	0.01617258
SLFN12	17q12	0.32099128	0.00327887	0.01619757
KLF3-AS1	4p14	0.32092598	0.00328557	0.01622259
LRRC40	1p31.1	0.32090421	0.0032878	0.01622959
RNMT	18p11.21	0.32088245	0.00329004	0.0162366
C6ORF62	6p22.3	0.32083891	0.00329451	0.01625466
CRKL	22q11.21	0.32055594	0.00332373	0.01638259
CSNK1G3	5q23.2	0.32053417	0.00332599	0.01638966
RNF138P1	5q11.2	0.32046887	0.00333277	0.01641901
SLC25A44	1q22	0.3204471	0.00333503	0.01642204
STK32A	5q32	0.3202911	0.00335129	0.01649375
MLANA	9p24.1	0.32028351	0.00335208	0.01649375
XPO1	2p15	0.32020766	0.00336001	0.0165287
OR2S2	9p13.3	0.32010434	0.00337084	0.0165697
KIAA0355	19q13.11	0.32007706	0.00337371	0.01657969
RAB31	18p11.22	0.31961994	0.00342204	0.01680476
PTPRM	18p11.23	0.31957641	0.00342667	0.01682338
CHST3	10q22.1	0.31955464	0.00342899	0.01682647
ZNF24	18q12.2	0.31935874	0.00344993	0.01691672
CHD4	12p13.31	0.3193152	0.0034546	0.01693544

KIAA1143	3p21.31	0.3192499	0.00346161	0.01696565
FAM122A	9q21.11	0.31922813	0.00346395	0.01697294
NCOA7	6q22.31-q22	0.31916283	0.00347098	0.01700321
ZNF304	19q13.43	0.3191193	0.00347568	0.01701783
AHR	7p21.1	0.31907576	0.00348038	0.01702829
MDM2	12q15	0.31907576	0.00348038	0.01702829
FAM120C	Xp11.22	0.31883632	0.00350633	0.01714683
RNF19B	1p35.1	0.31881455	0.00350869	0.01715419
ESCO2	8p21.1	0.31877102	0.00351343	0.01717315
TM7SF3	12p11.23	0.31870572	0.00352055	0.01719951
API5	11p12	0.31864042	0.00352769	0.01722591
SYBU	8q23.2	0.31857511	0.00353483	0.01725657
C10ORF131	10q24.1	0.31844538	0.00354907	0.01731804
BAG5	14q32.33	0.31842274	0.00355156	0.01732548
PRKG2	4q21.21	0.31833741	0.00356095	0.01736374
PCDHGB4	5q31.3	0.31833567	0.00356115	0.01736374
USP3	15q22.31	0.31831391	0.00356355	0.0173712
CNOT6L	4q21.1	0.3182486	0.00357076	0.01740209
NFATC3	16q22.1	0.3180527	0.00359247	0.01749505
CCSER2	10q23.1	0.31800916	0.00359731	0.01751006
FLG	1q21.3	0.31791316	0.003608	0.01755782
ACSL4	Xq23	0.31783503	0.00361673	0.01759599
SLC36A1	5q33.1	0.31774796	0.00362647	0.01763478
ZNF75D	Xq26.3	0.31770442	0.00363135	0.0176456
SNX29	16p13.13-p1	0.31766089	0.00363624	0.01766503
CHML	1q43	0.31761735	0.00364113	0.01768018
SRSF11	1p31.1	0.31759559	0.00364358	0.01768776
PTPRC	1q31.3-q32.1	0.31753028	0.00365094	0.01771914
FAN1	15q13.3	0.31748675	0.00365585	0.0177257
OSBPL3	7p15.3	0.31748675	0.00365585	0.0177257
SLC9A7	Xp11.3|Xp11	0.31746498	0.00365831	0.01772898
UBFD1	16p12.2	0.31739968	0.00366569	0.01776044
ZNF385B	2q31.2-q31.3	0.31737964	0.00366795	0.01776703
LILRA1	19q13.42	0.31736892	0.00366917	0.01776703
BCORL1	Xq26.1	0.31735615	0.00367062	0.01776703
OSBPL11	3q21.2	0.31735615	0.00367062	0.01776703
ARHGAP20	11q22.3-q23	0.31733438	0.00367308	0.01777033
CD226	18q22.2	0.31733438	0.00367308	0.01777033
ENDOD1	11q21	0.31726908	0.00368049	0.0177932
PHAX	5q23.2	0.31726908	0.00368049	0.0177932
FBXO40	3q13.33	0.31725299	0.00368232	0.01779771
ZNF669	1q44	0.31718201	0.00369039	0.01782375
PLOD2	3q24	0.31711671	0.00369783	0.01785102
ZNF674	Xp11.3	0.31711671	0.00369783	0.01785102
NCOA6	20q11.22	0.31696433	0.00371524	0.0179177
NPEPPS	17q21.32	0.31687727	0.00372522	0.01796148
UTP25	1q32.2	0.3168555	0.00372772	0.01796918
PPP6R3	11q13.2	0.31676843	0.00373773	0.01801308

ADCY2	5p15.31	0.31669397	0.00374631	0.0180457
NOCT	4q31.1	0.31657252	0.00376034	0.01810892
RNF115	1q21.1	0.31655076	0.00376286	0.01811667
KIF20B	10q23.31	0.31650722	0.00376791	0.01813658
F5	1q24.2	0.31648545	0.00377043	0.01814434
TRIM61	4q32.3	0.31643095	0.00377676	0.01816767
ITGBL1	13q33.1	0.31639838	0.00378054	0.01817545
SLC12A2	5q23.3	0.31628955	0.00379322	0.01823199
SELE	1q24.2	0.31626778	0.00379576	0.01823979
IFIH1	2q24.2	0.31622425	0.00380084	0.01825542
ACOX1	17q25.1	0.31611541	0.00381358	0.01830776
CDC42EP3	2p22.2	0.31611541	0.00381358	0.01830776
TMEM229A	7q31.32	0.31607277	0.00381858	0.01832344
PCYOX1	2p13.3	0.31607188	0.00381869	0.01832344
RASGRP1	15q14	0.31587597	0.00384173	0.01842516
QTRT2	3q13.31	0.31581067	0.00384945	0.01845769
ADAMTSL1	9p22.2-p22.1	0.31576713	0.00385459	0.01847348
HLTF	3q24	0.31574537	0.00385717	0.01848138
COG5	7q22.3	0.31570183	0.00386233	0.01849275
ARMH4	14q23.1	0.31557123	0.00387784	0.01855808
HS2ST1	1p22.3	0.31550593	0.00388561	0.01859082
RBM12	20q11.22	0.31535356	0.00390381	0.01866891
ZNF383	19q13.12	0.31533179	0.00390641	0.01867689
SLC8A1	2p22.1	0.31526649	0.00391424	0.01870532
SPTAN1	9q34.11	0.31526649	0.00391424	0.01870532
TSPAN12	7q31.31	0.31513588	0.00392993	0.01875782
CPNE8	12q12	0.31507058	0.0039378	0.01878637
MARVELD2	5q13.2	0.31498351	0.00394831	0.0188275
ASXL1	20q11.21	0.31491821	0.00395621	0.01886066
PRSS23	11q14.2	0.31478761	0.00397206	0.01893167
PXDNL	8q11.22-q11	0.31476584	0.0039747	0.01893975
PRR26	10p15.3	0.31475738	0.00397573	0.01894012
AFAP1	4p16.1	0.31474407	0.00397735	0.0189433
FMNL3	12q13.12	0.31454817	0.00400125	0.01904347
PCDH19	Xq22.1	0.3145264	0.00400391	0.01904704
STXBP4	17q22	0.31450463	0.00400658	0.01905062
ESYT2	7q36.3	0.31426519	0.004036	0.01918135
ZNF100	19p12	0.31415635	0.00404944	0.01923144
BCL2L13	22q11.21	0.31413459	0.00405213	0.01923964
CEP135	4q12	0.31398222	0.00407102	0.01931091
MAL2	8q24.12	0.31393868	0.00407643	0.01933197
KLHL33	14q11.2	0.31387713	0.00408409	0.0193637
DSP	6p24.3	0.31376454	0.00409814	0.01942105
UBE3C	7q36.3	0.31374278	0.00410086	0.01942932
LIPH	3q27.2	0.31363394	0.00411449	0.01946611
ZNF432	19q13.41	0.31363394	0.00411449	0.01946611
ANOS1	Xp22.31	0.3135904	0.00411995	0.0194827
CPNE3	8q21.3	0.3133945	0.00414461	0.01958072

STK3	8q22.2	0.31335096	0.00415011	0.0195974
GRK3	22q12.1	0.3133292	0.00415286	0.0196011
CDON	11q24.2	0.31330743	0.00415561	0.01960945
AMOTL1	11q21	0.31324213	0.00416388	0.01964381
RIN2	20p11.23	0.31322036	0.00416664	0.01965218
ANKRD20A9I	13q11	0.31317683	0.00417216	0.01966426
STK17B	2q32.3	0.31317683	0.00417216	0.01966426
TMEM170A	16q23.1	0.31317683	0.00417216	0.01966426
RASAL2	1q25.2	0.31313329	0.0041777	0.01968102
ANGPTL7	1p36.22	0.31311829	0.0041796	0.01968535
DENND4C	9p22.1	0.31308976	0.00418323	0.01969313
PTGIS	20q13.13	0.31308976	0.00418323	0.01969313
ADAM17	2p25.1	0.31304622	0.00418878	0.01971365
HNRNPLL	2p22.1	0.31302446	0.00419155	0.01971365
SLC7A6	16q22.1	0.31302446	0.00419155	0.01971365
SFMBT1	3p21.1	0.31287208	0.00421102	0.01979494
THOC2	Xq25	0.31285032	0.0042138	0.01979494
ADGRG6	6q24.2	0.31285032	0.0042138	0.01979494
HSPH1	13q12.3	0.31269795	0.00423336	0.01987744
MATN3	2p24.1	0.31245851	0.00426426	0.02001308
FAM155A	13q33.3	0.31241497	0.0042699	0.0200301
HIVEP3	1p34.2	0.3123932	0.00427272	0.02003391
SULT1C4	2q12.3	0.31237314	0.00427532	0.02004139
CCDC121	2p23.3	0.31221907	0.00429536	0.0201211
TRPC1	3q23	0.31221907	0.00429536	0.0201211
KLHL15	Xp22.11	0.3121973	0.00429819	0.02012965
MSRB3	12q14.3	0.31206669	0.00431525	0.02020478
TSPYL1	6q22.1	0.31202316	0.00432095	0.02022196
MMP16	8q21.3	0.31195786	0.00432951	0.02024776
PGBD4	15q14	0.31195786	0.00432951	0.02024776
KIF14	1q32.1	0.31189256	0.00433809	0.0202831
CDC14C	7p12.3	0.31169665	0.00436391	0.02039425
CHMP3	2p11.2	0.31169665	0.00436391	0.02039425
PTPN9	15q24.2	0.31167488	0.00436678	0.02040291
TTLL5	14q24.3	0.31163135	0.00437254	0.02042503
FNBP1L	1p22.1	0.31160958	0.00437543	0.02042891
RAP2A	13q32.1	0.31160958	0.00437543	0.02042891
LRRC37A6P	10p12.1	0.31152251	0.00438697	0.02047802
ADAM23	2q33.3	0.31143544	0.00439854	0.02052242
PAQR3	4q21.21	0.31141368	0.00440144	0.02052632
GREM1	15q13.3	0.31139191	0.00440434	0.02052842
DCC	18q21.2	0.31139114	0.00440444	0.02052842
MS4A2	11q12.1	0.31138709	0.00440498	0.02052842
ARNTL	11p15.3	0.31137014	0.00440724	0.02053414
COPB1	11p15.2	0.31134837	0.00441015	0.02054286
DSE	6q22.1	0.31132661	0.00441305	0.02055158
CPM	12q15	0.31117424	0.00443343	0.02063683
MCM9	6q22.31	0.31117424	0.00443343	0.02063683

CLMP	11q24.1	0.31104363	0.00445096	0.0206991
SPAG1	8q22.2	0.31104363	0.00445096	0.0206991
OAS3	12q24.13	0.3110001	0.00445682	0.0207215
TSHZ3	19q12	0.31095656	0.00446268	0.02074393
FOSL2	2p23.2	0.31091303	0.00446856	0.02076154
PTPN12	7q11.23	0.31086949	0.00447443	0.020784
RPE	2q34	0.31071712	0.00449506	0.02086523
TMEM26	10q21.2	0.31060829	0.00450985	0.02092412
ZDHHC17	12q21.2	0.31060829	0.00450985	0.02092412
IGSF9B	11q25	0.3105935	0.00451186	0.02092858
LOC646999	7p14.1	0.31052291	0.00452148	0.02095962
SMG7	1q25.3	0.31045592	0.00453063	0.02099117
PLS3	Xq23	0.31043415	0.0045336	0.02100006
TMEM184B	22q13.1	0.31039061	0.00453956	0.02101787
CYSLTR1	Xq21.1	0.31026001	0.00455746	0.02108117
LOC646214	15q11.2	0.31021648	0.00456344	0.02110395
FAM106A	17p11.2	0.3101577	0.00457153	0.02113644
SGO2	2q33.1	0.31012941	0.00457543	0.02114955
SHE	1q21.3	0.31002057	0.00459045	0.02120915
LTBP2	14q24.3	0.3099335	0.0046025	0.02125496
TGFBR3	1p22.1	0.30991173	0.00460552	0.02126396
PEX12	17q12	0.30984643	0.00461458	0.02129592
TOX4	14q11.2	0.30978113	0.00462366	0.02133286
PAPPA	9q33.1	0.30967229	0.00463882	0.02139291
EFCAB5	17q11.2	0.30962243	0.00464578	0.02142005
MGC15885	15q22.2	0.30958534	0.00465096	0.02143411
PLA2G4A	1q31.1	0.30958522	0.00465098	0.02143411
ABCA9	17q24.2	0.30941109	0.00467539	0.02153663
MPEG1	11q12.1	0.30938932	0.00467845	0.02154076
DDX58	9p21.1	0.30923695	0.00469991	0.02161959
PGR	11q22.1	0.30906281	0.00472455	0.02171286
ZNF441	19p13.2	0.30901927	0.00473072	0.02173122
ARMC8	3q22.3	0.30897574	0.00473691	0.02175461
SLC8A3	14q24.2	0.3088993	0.00474778	0.02179953
PPM1H	12q14.1-q14	0.3087363	0.00477105	0.02190131
SEC24B	4q25	0.308671	0.0047804	0.02193411
UBQLN1	9q21.32|9q2	0.308671	0.0047804	0.02193411
FGD5	3p25.1	0.30851863	0.00480228	0.02202942
PKP4	2q24.1	0.30840979	0.00481796	0.02209627
ZNF223	19q13.31	0.30834449	0.00482739	0.02212933
MAML1	5q35.3	0.30827919	0.00483684	0.02216753
COPA	1q23.2	0.30823565	0.00484314	0.02218623
IGDCC4	15q22.31	0.30819212	0.00484946	0.02220494
FBXO3	11p13	0.30817035	0.00485262	0.0222092
INHBC	12q13.3	0.30815913	0.00485425	0.02221156
ATP1B1	1q24.2	0.30806151	0.00486844	0.02226629
APOL6	22q12.3	0.30773501	0.0049162	0.02246923
ZNF300P1	5q33.1	0.30769314	0.00492235	0.02249219

ZNF776	19q13.43	0.3076697	0.0049258	0.02250278
SHTN1	10q25.3	0.3074085	0.00496437	0.02266859
POLR3A	10q22.3	0.30732143	0.00497729	0.02271716
PROKR2	20p12.3	0.30727712	0.00498387	0.022742
SUN1	7p22.3	0.30723436	0.00499024	0.0227606
UNC119B	12q24.31	0.30723436	0.00499024	0.0227606
SLFN11	17q12	0.30703845	0.00501947	0.02288347
CRK	17p13.3	0.30686431	0.00504559	0.0229815
RAB6A	11q13.4	0.30662487	0.0050817	0.02313538
CSF2RB	22q12.3	0.30660311	0.00508499	0.02314509
PTPDC1	9q22.32	0.30658134	0.00508829	0.0231548
C7ORF69	7p12.3	0.30639719	0.00511625	0.0232767
TLR8	Xp22.2	0.30632013	0.00512799	0.02331946
BTN2A1	6p22.2	0.30614599	0.0051546	0.02342446
DACT1	14q23.1	0.30612423	0.00515794	0.02342892
TMCC3	12q22	0.30612423	0.00515794	0.02342892
ZNF618	9q32	0.30610246	0.00516128	0.02343874
SLC35B3	6p24.3	0.30599362	0.00517799	0.02350394
ATG14	14q22.3	0.30581948	0.00520484	0.02361504
GPR142	17q25.1	0.30563648	0.00523319	0.02372572
ANLN	7p14.2	0.30562358	0.00523519	0.02372572
TBX20	7p14.2	0.3055783	0.00524223	0.0237522
SLC22A15	1p13.1	0.30553651	0.00524873	0.02377626
PPM1A	14q23.1	0.30547121	0.0052589	0.02381152
SNX27	1q21.3	0.30531884	0.00528271	0.02390302
MKLN1	7q32.3	0.30516647	0.00530662	0.02398938
SRSF10	1p36.11	0.30490526	0.00534782	0.02414277
UBR7	14q32.12	0.30488349	0.00535127	0.02415285
GPC6	13q31.3-q32	0.30479642	0.00536507	0.02420419
CHST15	10q26.13	0.30468759	0.00538237	0.02427125
VPS41	7p14.1	0.30457875	0.00539972	0.02434397
XRN2	20p11.22	0.30453521	0.00540668	0.02436429
CLEC6A	12p13.31	0.30448467	0.00541476	0.02438417
CHIC1	Xq13.2	0.30446991	0.00541712	0.02438929
LINC00928	15q26.1	0.30442482	0.00542435	0.02441078
INTS4P1	7q11.21	0.30433931	0.00543807	0.02445512
NID1	1q42.3	0.30431754	0.00544156	0.02445512
FAM126A	7p15.3	0.30429577	0.00544506	0.02446532
GALNT15	3p25.1	0.30423047	0.00545558	0.02449598
C8ORF37	8q22.1	0.30416517	0.0054661	0.02453219
ZNF25	10p11.21	0.30416517	0.0054661	0.02453219
CPD	17q11.2	0.3041434	0.00546962	0.0245369
KLHL23	2q31.1	0.30412164	0.00547313	0.02454162
OR52K1	11p15.4	0.30406113	0.00548292	0.0245689
SLC5A8	12q23.1-q23	0.30403123	0.00548776	0.02458505
POLD3	11q13.4	0.3040128	0.00549074	0.0245929
MYO10	5p15.1	0.30399103	0.00549427	0.02460316
GDAP2	1p12	0.30396926	0.0054978	0.02461344

PPFIA2	12q21.31	0.30395411	0.00550026	0.02461891
UAP1	1q23.3	0.30368629	0.00554387	0.02480296
YIPF5	5q31.3	0.30368629	0.00554387	0.02480296
ZNF45	19q13.31	0.30366452	0.00554743	0.02480774
PIGO	9p13.3	0.30366452	0.00554743	0.02480774
CAMSAP1	9q34.3	0.30364276	0.00555099	0.02481252
WDR11	10q26.12	0.30364276	0.00555099	0.02481252
NR3C2	4q31.23	0.30362099	0.00555455	0.02482287
COL24A1	1p22.3	0.30351215	0.00557239	0.02489142
ABCD2	12q12	0.30349038	0.00557596	0.02490181
COL10A1	6q22.1	0.30338155	0.00559387	0.02495378
PHLDB2	3q13.2	0.30338155	0.00559387	0.02495378
DCAF16	4p15.31	0.30331625	0.00560463	0.02498501
PEG3-AS1	19q13.43	0.30322132	0.00562031	0.02503811
NFXL1	4p12	0.30318564	0.00562622	0.0250532
RSBN1L	7q11.23	0.30316388	0.00562982	0.02506365
SCN4B	11q23.3	0.30312034	0.00563704	0.02508456
CUBN	10p13	0.30309857	0.00564065	0.02509502
STIL	1p33	0.3030115	0.00565511	0.02515375
FCF1	14q24.3	0.30298974	0.00565873	0.02516424
NSUN3	3q11.2	0.30285913	0.0056805	0.02522725
ZNF44	19p13.2	0.30279383	0.00569141	0.02526445
ZNF700	19p13.2	0.30268499	0.00570964	0.02532843
GDAP1	8q21.11	0.30259793	0.00572426	0.02538198
C5ORF58	5q35.1	0.30258733	0.00572604	0.02538423
MICAL3	22q11.21	0.30253262	0.00573525	0.02540807
LOC641367	19p12	0.30224041	0.00578464	0.02561085
ANKH	5p15.2	0.30220611	0.00579046	0.02561849
ZNF566	19q13.12	0.30220611	0.00579046	0.02561849
NAP1L3	Xq21.32	0.30209728	0.00580897	0.02567187
MUC16	19p13.2	0.30207551	0.00581268	0.02568167
RXFP4	1q22	0.30206912	0.00581377	0.02568167
OGT	Xq13.1	0.30201021	0.00582382	0.02571446
ARGFXP2	17q11.2	0.3020029	0.00582506	0.02571446
BNIP2	15q22.2	0.30196667	0.00583125	0.02573607
OGN	9q22.31	0.30179254	0.00586108	0.02583907
ACVR2B	3p22.2	0.30177077	0.00586481	0.02584411
RB1CC1	8q11.23	0.30177077	0.00586481	0.02584411
ZNF561	19p13.2	0.301749	0.00586855	0.02585487
SEC22B	1p12	0.30166193	0.00588353	0.0259094
DHX8	17q21.31	0.30164016	0.00588728	0.02592018
GPR21	9q33.2	0.30162946	0.00588913	0.02592258
CRPPA	7p21.2	0.30142249	0.0059249	0.02606276
GCKR	2p23.3	0.30140439	0.00592804	0.02606785
PCDHB11	5q31.3	0.30140072	0.00592867	0.02606785
GREB1L	18q11.1-q11	0.30136971	0.00593405	0.02607423
USP31	16p12.2	0.30131366	0.00594379	0.02609974
ADRA1A	8p21.2	0.30130383	0.0059455	0.02610148

PUM2	2p24.1	0.30127012	0.00595136	0.02612146
KANK2	19p13.2	0.30113952	0.00597412	0.02619948
RORB	9q21.13	0.30113794	0.0059744	0.02619948
ZNF131	5p12	0.30111775	0.00597792	0.02620917
RIC8B	12q23.3	0.30105245	0.00598934	0.02625345
ATP10A	15q12	0.30090008	0.00601605	0.02636474
LRRCC1	8q21.2	0.30081301	0.00603136	0.0264144
SYTL2	11q14.1	0.30057357	0.00607365	0.02657622
CD164	6q21	0.30057357	0.00607365	0.02657622
SUSD1	9q31.3-q32	0.30057357	0.00607365	0.02657622
FPR3	19q13.41	0.3005518	0.00607751	0.02658142
PLAG1	8q12.1	0.3005518	0.00607751	0.02658142
RLIM	Xq13.2	0.30039943	0.00610457	0.02667634
TBCK	4q24	0.30039943	0.00610457	0.02667634
DYNC2H1	11q22.3	0.30033413	0.0061162	0.02671544
KDM3B	5q31.2	0.30031236	0.00612008	0.02672067
CXADR	21q21.1	0.30024706	0.00613173	0.02675983
CCKAR	4p15.2	0.30022537	0.00613561	0.02677087
TAS2R9	12p13.2	0.30014449	0.00615008	0.02682814
TMC7	16p12.3	0.30007292	0.00616291	0.02687823
ULBP1	6q25.1	0.30005115	0.00616682	0.02688349
ADAMTS6	5q12.3	0.30000762	0.00617464	0.02689992
MEGF10	5q23.2	0.29994232	0.00618639	0.02693932
CFH	1q31.3	0.29992055	0.00619031	0.0269505
CORO1C	12q24.11	0.29968111	0.00623357	0.02710924
ZBTB21	21q22.3	0.29965934	0.00623752	0.02712048
CTTNBP2	7q31.31	0.29961581	0.00624542	0.02714891
IL12RB2	1p31.3	0.2994199	0.00628108	0.027292
POLR2M	15q21.3	0.29939813	0.00628505	0.02729677
CACNG8	19q13.42	0.29939069	0.00628641	0.02729677
TSPYL5	8q22.1	0.29937637	0.00628903	0.02729677
WFIKKN2	17q21.33	0.29933733	0.00629616	0.0273194
RPS6KC1	1q32.3	0.29933283	0.00629699	0.0273194
LMAN1	18q21.32	0.29918046	0.00632491	0.02742858
ZBTB8A	1p35.1	0.29915869	0.0063289	0.02743995
TMEM255A	Xq24	0.29913693	0.0063329	0.02745131
ADCYAP1R1	7p14.3	0.29910593	0.0063386	0.02747004
CARD8	19q13.33	0.29904986	0.00634893	0.02749085
RNF13	3q25.1	0.29902809	0.00635294	0.02750224
CXORF38	Xp11.4	0.29900632	0.00635695	0.02750766
PHF6	Xq26.2	0.29900632	0.00635695	0.02750766
GUCY1A2	11q22.3	0.29898456	0.00636097	0.02751905
CXCL12	10q11.21	0.29891925	0.00637303	0.02756524
FLVCR1	1q32.3	0.29876688	0.00640124	0.02766326
ATP11C	Xq27.1	0.29876688	0.00640124	0.02766326
AGMO	7p21.2	0.29875819	0.00640286	0.02766423
ZNF333	19p13.12	0.29867981	0.00641742	0.02772113
OR1Q1	9q33.2	0.29863733	0.00642532	0.02774927

DZIP3	3q13.13	0.29857098	0.00643769	0.02779663
SLC25A32	8q22.3	0.29841861	0.00646616	0.02790142
CCDC47	17q23.3	0.29839684	0.00647023	0.02791296
EPB41	1p35.3	0.29833154	0.00648248	0.02795972
PCDHGA1	5q31.3	0.29826624	0.00649474	0.02800655
ZNF860	3p23-p22.3	0.2982227	0.00650293	0.02803578
ABCD3	1p21.3	0.2981574	0.00651522	0.02807664
F2R	5q13.3	0.2980268	0.00653988	0.02817071
ZNF141	4p16.3	0.29796149	0.00655224	0.02821174
LINC00654	20p12.3	0.29793973	0.00655636	0.0282234
TMOD2	15q21.2	0.29789619	0.00656462	0.02825283
ZNF469	16q24.2	0.29776559	0.00658944	0.02833516
ZNF514	2q11.1	0.29759145	0.00662266	0.02845345
EDA2R	Xq12	0.29750438	0.00663933	0.02851891
LAMP2	Xq24	0.29748261	0.0066435	0.02852453
TIMP2	17q25.3	0.29748261	0.0066435	0.02852453
MYO16	13q33.3	0.29744555	0.00665061	0.0285489
PYGO1	15q21.3	0.29741893	0.00665572	0.02856468
CSNK1G2-AS	19p13.3	0.29738917	0.00666144	0.02858307
ADGRG2	Xp22.13	0.29737378	0.0066644	0.02858909
OR3A1	17p13.3	0.2973543	0.00666815	0.02858909
COL5A2	2q32.2	0.29722141	0.00669375	0.02867847
R3HDM2	12q13.3	0.2970255	0.00673166	0.02882847
RGS22	8q22.2	0.29685349	0.0067651	0.02895923
PCMTD1	8q11.23	0.29676429	0.0067825	0.02902123
GPR83	11q21	0.29674253	0.00678675	0.02902695
OR10AD1	12q13.11	0.29672589	0.00679	0.02903462
ARHGAP29	1p22.1	0.29669899	0.00679526	0.02904464
HHAT	1q32.2	0.29667722	0.00679952	0.02905661
ZKSCAN2	16p12.1	0.29663369	0.00680805	0.0290868
ZFP69B	1p34.2	0.29648132	0.00683796	0.0291958
JAK2	9p24.1	0.29641602	0.00685081	0.02923189
ANKRD11	16q24.3	0.29641602	0.00685081	0.02923189
ABCA5	17q24.3	0.29639425	0.0068551	0.02923766
TRMT13	1p21.2	0.29639425	0.0068551	0.02923766
NUDCD1	8q23.1	0.29635071	0.00686369	0.02926175
IPO9	1q32.1	0.29622011	0.0068895	0.02936552
ARNT2	15q25.1	0.29617658	0.00689813	0.02938342
ZNF117	7q11.21	0.29617658	0.00689813	0.02938342
TRAF5	1q32.3	0.29593714	0.00694573	0.02955458
C18ORF25	18q21.1	0.29587183	0.00695876	0.02959739
LOC1001290	9q33.3	0.29580653	0.00697181	0.02964026
ADCY9	16p13.3	0.29574123	0.00698489	0.02967051
VPS8	3q27.2	0.29574123	0.00698489	0.02967051
ANKRD31	5q13.3	0.29566092	0.007001	0.02973261
ZNF684	1p34.2	0.29563239	0.00700673	0.02974426
ZBED9	6p22.1	0.29550479	0.00703242	0.02984693
UBE2Z	17q21.32	0.29548002	0.00703741	0.02986176

ZNF154	19q13.43	0.29545826	0.0070418	0.02987404
INPP4A	2q11.2	0.29541472	0.00705059	0.0298986
PPP1R2B	5q33.3	0.29541472	0.00705059	0.0298986
SIGLEC5	19q13.41	0.29534942	0.0070638	0.02994185
SETBP1	18q12.3	0.29530588	0.00707262	0.02997284
ATP8B4	15q21.2	0.29526235	0.00708144	0.0299911
ADAM18	8p11.22	0.29515456	0.00710334	0.03007743
NME9	3q22.3	0.29510427	0.00711357	0.03011436
TIPARP	3q25.31	0.29508821	0.00711684	0.03012181
LOC202181	5q35.3	0.29502291	0.00713016	0.03017175
CCNE2	8q22.1	0.29497938	0.00713904	0.03019654
ENPP4	6p21.1	0.29495761	0.00714349	0.03020893
GOSR2	17q21.32	0.29491407	0.0071524	0.03023375
PRDM5	4q27	0.29478347	0.00717917	0.03033403
CACNB4	2q23.3	0.29473994	0.00718811	0.03036537
OR6K6	1q23.1	0.29466664	0.00720319	0.03041616
GPR174	Xq21.1	0.2946327	0.00721018	0.03043923
HP1BP3	1p36.12	0.29447873	0.00724197	0.0305605
TMEM267	5p12	0.29439166	0.00726	0.03061066
SNRNP200	2q11.2	0.29439166	0.00726	0.03061066
WBP2NL	22q13.2	0.29426619	0.00728606	0.03070752
TMEM161B	5q14.3	0.29423929	0.00729166	0.0307246
YWHAB	20q13.12	0.29415222	0.0073098	0.03078802
ZNF420	19q13.12	0.29408692	0.00732343	0.03083891
ZNF367	9q22.32|9q2	0.29402161	0.00733708	0.03087683
COG6	13q14.11	0.29397808	0.0073462	0.03089561
NOS1AP	1q23.3	0.29397808	0.0073462	0.03089561
ZKSCAN4	6p22.1	0.29384748	0.00737361	0.03099778
ANO4	12q23.1	0.29383815	0.00737557	0.03099948
MYLK4	6p25.2	0.29369511	0.00740569	0.03111298
FNDC7	1p13.3	0.29367942	0.007409	0.03112032
THSD4	15q23	0.29352097	0.00744252	0.03124132
HIST4H4	12p12.3	0.29341213	0.00746561	0.03133166
IRAK4	12q12	0.2933686	0.00747487	0.0313639
FKTN	9q31.2	0.29332506	0.00748413	0.03138955
NRP1	10p11.22	0.29332506	0.00748413	0.03138955
AOX1	2q33.1	0.29321623	0.00750734	0.03147364
MAPK8	10q11.22	0.29315092	0.0075213	0.03151888
GLP1R	6p21.2	0.29312861	0.00752607	0.03153225
FANCD2	3p25.3	0.29291148	0.00757267	0.03170746
HS3ST3B1	17p12	0.29288972	0.00757735	0.03172041
GPALPP1	13q14.12	0.29286795	0.00758204	0.03173336
EBAG9	8q23.2	0.29284618	0.00758673	0.03173965
EIF4G2	11p15.4	0.29284618	0.00758673	0.03173965
RBM5	3p21.31	0.29265028	0.00762906	0.03189626
ZNF782	9q22.33	0.29262851	0.00763377	0.03189626
ZNF793	19q13.12	0.29262851	0.00763377	0.03189626
PDC	1q31.1	0.29253372	0.00765434	0.03197547

DDX18	2q14.1	0.29251967	0.00765739	0.03198152
AQP7P1	9q13	0.29246109	0.00767013	0.032028
TMEM39A	3q13.33	0.2924326	0.00767633	0.03204718
FUT10	8p12	0.29221493	0.00772386	0.03222534
TUT4	1p32.3	0.29208433	0.0077525	0.03233807
PDIK1L	1p36.11	0.29201902	0.00776685	0.03239117
METTL21EP	13q33.1	0.29195854	0.00778017	0.03243312
MKI67	10q26.2	0.29191019	0.00779083	0.03245719
PTP4A2	1p35.2	0.29184489	0.00780525	0.03251046
PIK3AP1	10q24.1	0.29180135	0.00781488	0.03253556
COL28A1	7p21.3	0.29179546	0.00781618	0.03253556
CASQ2	1p13.1	0.29178594	0.00781829	0.03253754
ADCY7	16q12.1	0.29171428	0.00783416	0.03258316
CENPBD1	16q24.3	0.29167075	0.00784381	0.03261652
SPON1	11p15.2	0.29164898	0.00784865	0.0326298
SDR16C5	8q12.1	0.29161746	0.00785565	0.03265209
FYB1	5p13.1	0.29151838	0.0078777	0.03272324
PABPC4L	4q28.3	0.29149661	0.00788255	0.03272974
ABCA6	17q24.2-q24	0.29149661	0.00788255	0.03272974
ESCO1	18q11.2	0.29140954	0.00790197	0.03278307
DERL1	8q24.13	0.29138777	0.00790684	0.03279642
FAM177A1	14q13.2	0.29136601	0.0079117	0.03280977
ABL2	1q25.2	0.2913007	0.00792632	0.03284302
TRAPPC6B	14q21.1	0.2913007	0.00792632	0.03284302
RUFY2	10q21.3	0.29125717	0.00793607	0.03287661
LOC652276	16p13.3	0.2912354	0.00794096	0.03288999
LMO7	13q22.2	0.29112657	0.00796541	0.0329844
USPL1	13q12.3	0.29099596	0.00799483	0.03309938
KCNQ5	6q13	0.29097419	0.00799975	0.03311284
ANKAR	2q32.2	0.29093066	0.00800958	0.03314666
PSME4	2p16.2	0.29090889	0.00801451	0.03316014
SIPA1L2	1q42.2	0.29082182	0.00803422	0.0332279
STAT2	12q13.3	0.29082182	0.00803422	0.0332279
DOCK8	9p24.3	0.29058238	0.00808865	0.03339727
CTNNA3	10q21.3	0.29058218	0.0080887	0.03339727
MAP3K1	5q11.2	0.29056062	0.00809362	0.03339727
ZNF12	7p22.1	0.29056062	0.00809362	0.03339727
GABPA	21q21.3	0.29053885	0.00809858	0.03341085
LOC440173	9q21.33	0.29047472	0.00811323	0.03345161
TMEM211	22q11.23	0.29045882	0.00811687	0.03345856
TMEM150C	4q21.22	0.29032118	0.0081484	0.03356771
KCNH1	1q32.2	0.29029941	0.0081534	0.03358135
KAT2B	3p24.3	0.29021234	0.00817341	0.03365682
CYBB	Xp21.1-p11.4	0.29019057	0.00817842	0.03367048
NLN	5q12.3	0.29014704	0.00818845	0.0337048
TRIM4	7q22.1	0.29001643	0.0082186	0.03382191
DISC2	1q42.2	0.28991212	0.00824275	0.0339046
TPP1	11p15.4	0.2899076	0.0082438	0.0339046

ZNF646	16p11.2	0.2899076	0.0082438	0.0339046
EIF3A	10q26.11	0.28988583	0.00824885	0.03391836
ENPP2	8q24.12	0.28986406	0.0082539	0.03392512
SPOCK1	5q31.2	0.28986406	0.0082539	0.03392512
TANC2	17q23.2-q23	0.28982053	0.00826401	0.03395966
TNFSF8	9q32-q33.1	0.28979876	0.00826907	0.03397344
GGA2	16p12.2	0.28977699	0.00827413	0.03398723
IGIP	5q31.3	0.28975523	0.00827919	0.03399237
FBXL17	5q21.3	0.28973346	0.00828426	0.03399237
FOXI2	10q26.2	0.28972762	0.00828562	0.03399237
TRPA1	8q21.11	0.28965885	0.00830165	0.0340441
PDGFRB	5q32	0.28951579	0.00833508	0.0341601
COL14A1	8q24.12	0.28942872	0.00835548	0.03423668
TMEM47	Xp21.1	0.28938518	0.0083657	0.0342715
PKHD1L1	8q23.1-q23.2	0.28936177	0.0083712	0.03427993
FOXO3	6q21	0.28934165	0.00837593	0.03429225
LRRC58	3q13.33	0.28923281	0.00840155	0.03438282
CHSY1	15q26.3	0.28921104	0.00840668	0.03438282
SYNM	15q26.3	0.28918928	0.00841182	0.0343897
TTPA	8q12.3	0.28913645	0.00842429	0.03443363
CCDC170	6q25.1	0.28903691	0.00844784	0.03450157
GOSR1	17q11.2	0.28903691	0.00844784	0.03450157
RNF139	8q24.13	0.2889716	0.00846333	0.03454355
ZNF772	19q13.43	0.28894984	0.00846849	0.03455755
TET3	2p13.1	0.2889063	0.00847883	0.03459266
ELF2	4q31.1	0.28881923	0.00849954	0.03464877
CD83	6p23	0.28866686	0.0085359	0.03477562
SNORA54	11p15.4	0.2885786	0.00855702	0.03483922
TEX11	Xq13.1	0.28857239	0.00855851	0.03483922
SSR1	6p24.3	0.28853626	0.00856717	0.03486023
MAP3K20	2q31.1	0.28853626	0.00856717	0.03486023
DIDO1	20q13.33	0.28838389	0.00860377	0.0349806
PPM1E	17q22	0.28836212	0.00860901	0.03498763
SMG1P5	16p11.2	0.28836212	0.00860901	0.03498763
OR2L1P	1q44	0.28831727	0.00861982	0.0350244
PCDH20	13q21.2	0.28828923	0.00862658	0.03504433
PDZD2	5p13.3	0.28827505	0.00863001	0.03504433
PGM2	4p14	0.28825328	0.00863526	0.03505852
PTPRF	1p34.2	0.28818798	0.00865104	0.03511495
OR56B1	11p15.4	0.28818119	0.00865268	0.03511495
DNAH1	3p21.1	0.28810091	0.00867212	0.03518667
MPDZ	9p23	0.28805738	0.00868268	0.03520799
TLR5	1q41	0.28790501	0.00871972	0.03532941
DLAT	11q23.1	0.28786147	0.00873033	0.03534363
DNER	2q36.3	0.28786147	0.00873033	0.03534363
SLC25A51P4	11p15.1	0.28775947	0.00875523	0.03542284
CDH19	18q22.1	0.28773935	0.00876015	0.03543554
FAM107B	10p13	0.2876438	0.00878354	0.03551574

HECA	6q24.1	0.28749143	0.00882096	0.03563087
NUP133	1q42.13	0.28742613	0.00883703	0.03568858
ZNF33A	10p11.1	0.28738259	0.00884777	0.03571744
ZNF594	17p13.2	0.28733906	0.00885851	0.03575353
TBX15	1p12	0.28731729	0.00886389	0.03575353
CWF19L2	11q22.3	0.28725199	0.00888004	0.03580416
HCFC1	Xq28	0.28701255	0.00893946	0.03601461
ATP8B1	18q21.31	0.28696901	0.0089503	0.0360437
IMPACT	18q11.2	0.28692548	0.00896116	0.03608012
WWC2	4q35.1	0.28690371	0.00896659	0.03609469
TPTE	21p11.2	0.28687042	0.0089749	0.03612085
RIT1	1q22	0.28686018	0.00897746	0.03612385
CLCN5	Xp11.23	0.28681664	0.00898834	0.03616033
WIPF1	2q31.1	0.28679488	0.00899379	0.03617493
KPNA5	6q22.1	0.28672957	0.00901014	0.03622608
GALNT10	5q33.2	0.28672957	0.00901014	0.03622608
MCTS2P	20q11.21	0.28668604	0.00902106	0.03626266
ZNF391	6p22.1	0.2865772	0.0090484	0.03635056
NXPE2	11q23.2-q23	0.28653433	0.00905919	0.03637991
TLR1	4p14	0.28629423	0.00911984	0.03658588
TMEM212	3q26.31	0.28624218	0.00913303	0.0366312
RAB3B	1p32.3	0.28623516	0.00913481	0.0366312
PSD3	8p22	0.2861543	0.00915534	0.03670614
TGDS	13q32.1	0.28601125	0.00919176	0.03682992
HEATR1	1q43	0.28598949	0.00919732	0.03684476
KRT18P55	17q11.2	0.28590821	0.00921808	0.03692051
MTO1	6q13	0.28585888	0.0092307	0.03695619
RAB33B	4q31.1	0.28575005	0.0092586	0.03706044
ST6GAL1	3q27.3	0.28557591	0.00930339	0.03720236
SEC23B	20p11.23	0.28557591	0.00930339	0.03720236
GCSAML	1q44	0.28552035	0.00931772	0.03725218
ALDH1B1	9p13.1	0.28548884	0.00932586	0.03727723
VEZT	12q22	0.28546707	0.00933148	0.03729223
ARID5B	10q21.2	0.28542354	0.00934274	0.03732973
ZNF721	4p16.3	0.28533647	0.00936529	0.03739732
RIOK3	18q11.2	0.28529293	0.00937658	0.03743491
ATP6V0A2	12q24.31	0.2852494	0.00938789	0.03747254
DAPP1	4q23	0.28479229	0.00950731	0.03791883
ST6GALNAC2	17q25.1	0.28477052	0.00951303	0.03793405
GBE1	3p12.2	0.28474875	0.00951875	0.03794927
GAREM1	18q12.1	0.28468345	0.00953594	0.03801018
FDPSP2	7q11.23	0.28455439	0.00956999	0.03813826
CD274	9p24.1	0.28448754	0.00958766	0.03820106
ZBTB34	9q33.3	0.28444401	0.00959919	0.03823934
CYP20A1	2q33.2	0.28420457	0.00966281	0.03844663
NOS1	12q24.22	0.28416045	0.00967457	0.03848575
C3ORF49	3p14.1	0.28409803	0.00969123	0.03853138
FLJ42393	3q27.3	0.28407706	0.00969684	0.03854352

E2F8	11p15.1	0.2839869	0.00972096	0.0386086
FAM98B	15q14	0.28389983	0.00974431	0.03867048
TJP2	9q21.11	0.28389983	0.00974431	0.03867048
RELN	7q22.1	0.28379099	0.00977356	0.03877112
TOPORS	9p21.1	0.28370392	0.00979702	0.0388487
HPCAL4	1p34.2	0.28364016	0.00981423	0.0389031
C14ORF178	14q24.3	0.28362934	0.00981715	0.0389053
SKP2	5p13.2	0.28355155	0.00983819	0.03898091
OTULINL	5p15.2	0.28352978	0.00984408	0.0389965
KCNB1	20q13.13	0.28349534	0.00985341	0.03902571
CCR8	3p22.1	0.28347691	0.00985841	0.03903774
RBM33	7q36.3	0.28346448	0.00986178	0.03904332
OLFML1	11p15.4	0.28331211	0.00990319	0.03919946
ZNF550	19q13.43	0.28313797	0.00995069	0.03935725
SENP5	3q29	0.28313797	0.00995069	0.03935725
SPDYE6	7q22.1	0.28313797	0.00995069	0.03935725
NIPA1	15q11.2	0.28309444	0.0099626	0.0393955
ZC3H12B	Xq11.2-q12	0.28302914	0.00998049	0.0394349
AFAP1L1	5q32	0.28296383	0.0099984	0.03948219
LAMC2	1q25.3	0.28296383	0.0099984	0.03948219
FREM2	13q13.3	0.2829357	0.01000613	0.03949704
TDGF1	3p21.31	0.28285523	0.01002825	0.03956869
TAF13	1p13.3	0.28281146	0.01004031	0.03960841
ZNF714	19p12	0.28270263	0.01007034	0.03971114
CHSY3	5q23.3	0.28268086	0.01007635	0.03971913
CDK6	7q21.2	0.28268086	0.01007635	0.03971913
RTL3	Xq21.1	0.28261463	0.01009467	0.0397756
SLC6A20	3p21.31	0.28259379	0.01010044	0.03979047
ZNF101	19p13.11	0.28255026	0.01011251	0.03982224
OR51Q1	11p15.4	0.28251693	0.01012175	0.03984617
GNG12	1p31.3	0.28250672	0.01012458	0.03984617
IPO7	11p15.4	0.28246319	0.01013667	0.03988586
LINC01124	2q31.1	0.28230469	0.01018079	0.04004365
DMGDH	5q14.1	0.28228905	0.01018516	0.0400529
KIF23	15q23	0.28224551	0.01019731	0.04009278
TMED10	14q24.3	0.28222375	0.01020339	0.04009294
GNE	9p13.3	0.28209314	0.01023994	0.04022071
CNNM4	2q11.2	0.28200607	0.01026438	0.0402849
SLF1	5q15	0.28194077	0.01028273	0.04034105
CLMN	14q32.13	0.28181017	0.01031953	0.04046152
FBXO11	2p16.3	0.28181017	0.01031953	0.04046152
ZNF483	9q31.3	0.28178993	0.01032525	0.04047594
C9ORF131	9p13.3	0.28165597	0.01036313	0.04060048
ELOVL5	6p12.1	0.28163603	0.01036878	0.04060665
ANKRD20A3	9q21.11	0.28159171	0.01038135	0.04063987
NBPF11	1q21.2	0.28154896	0.01039348	0.04067938
ZNF569	19q13.12	0.28152719	0.01039967	0.04069559
ARMCX3	Xq22.1	0.28150543	0.01040585	0.0407038

TTC3	21q22.13	0.28150543	0.01040585	0.0407038
IMPA1	8q21.13	0.28144012	0.01042443	0.04076847
SLC39A6	18q12.2	0.28128775	0.01046789	0.04091434
FKBP9P1	7p11.2	0.28126599	0.01047411	0.0409226
RTN1	14q23.1	0.28126599	0.01047411	0.0409226
CANT1	17q25.3	0.28124422	0.01048034	0.04093889
B4GALT6	18q12.1	0.28117892	0.01049903	0.0409878
NAB1	2q32.2	0.28117892	0.01049903	0.0409878
ZNF780B	19q13.2	0.28109185	0.010524	0.04106918
NEGR1	1p31.1	0.28107008	0.01053025	0.04108553
RANBP9	6p23	0.28104831	0.01053651	0.04109383
SEC14L1	17q25.2-q25	0.28104831	0.01053651	0.04109383
ADRA2A	10q25.2	0.28102655	0.01054277	0.04109409
HIST2H2BA	1p11.2	0.28102655	0.01054277	0.04109409
SPOP	17q21.33	0.28096124	0.01056156	0.0411271
DGKI	7q33	0.28096124	0.01056156	0.0411271
MSL1	17q21.1	0.28096124	0.01056156	0.0411271
ZNF788P	19p13.2	0.28089594	0.01058038	0.04119233
APPL2	12q23.3	0.28085241	0.01059294	0.04123319
PLEKHA2	8p11.22	0.28074357	0.01062441	0.04134758
ZFP3	17p13.2	0.28063473	0.01065595	0.04146226
CLCN6	1p36.22	0.28048236	0.01070026	0.04160213
DENND5B	12p11.21	0.28035176	0.01073836	0.04172583
TRIM39	6p22.1	0.28032999	0.01074472	0.04174241
USO1	4q21.1	0.28022116	0.01077658	0.04184169
RXFP3	5p13.2	0.28015749	0.01079525	0.04190603
SOGA3	6q22.33	0.28013409	0.01080212	0.04191637
MBTPS1	16q23.3-q24	0.28011232	0.01080852	0.04193301
PKD1L3	16q22.2	0.28005602	0.01082507	0.04197271
KIAA0319L	1p34.3	0.27972051	0.01092419	0.04229116
HS3ST5	6q21-q22.1	0.27965779	0.01094281	0.04235499
MIAT	22q12.1	0.27963344	0.01095004	0.04237476
MDM4	1q32.1	0.2795899	0.01096299	0.04241662
ZNF132	19q13.43	0.27955877	0.01097225	0.04244422
FGF2	4q28.1	0.27943753	0.0110084	0.04254274
XKR5	8p23.1	0.27931812	0.0110441	0.04267245
MAFG	17q25.3	0.27919809	0.01108009	0.04279948
CLEC4C	12p13.31	0.27919415	0.01108128	0.04279948
CCN4	8q24.22	0.27915456	0.01109317	0.04283711
ZNF740	12q13.13	0.27913279	0.01109972	0.04285408
RMI1	9q21.32	0.27911102	0.01110627	0.04286274
ACVR2A	2q22.3-q23.1	0.27911102	0.01110627	0.04286274
LOC645752	15q24.3	0.27903764	0.01112837	0.04293972
ELMO2	20q13.12	0.27898042	0.01114562	0.04298965
USP15	12q14.1	0.27898042	0.01114562	0.04298965
KL	13q13.1	0.27895865	0.0111522	0.04300667
CBX7	22q13.1	0.27893689	0.01115877	0.0430237
PIK3C3	18q12.3	0.27884982	0.01118511	0.04310854

NBPF15	1q21.1	0.27882805	0.0111917	0.0431256
PAPOLA	14q32.2	0.27867568	0.01123793	0.04328702
STAM	10p12.33	0.27863214	0.01125118	0.04332127
SYPL1	7q22.3	0.27861038	0.0112578	0.0433384
EPPIN	20q13.12	0.27851315	0.01128744	0.04343256
HTR2B	2q37.1	0.27850154	0.01129098	0.04343256
SLC4A4	4q13.3	0.27847977	0.01129763	0.04344973
BRWD1	21q22.2	0.27834917	0.01133758	0.04357813
ST18	8q11.23	0.27828387	0.0113576	0.04363824
PRMT9	4q31.23	0.27821857	0.01137765	0.04370685
THSD7B	2q22.1	0.27820105	0.01138303	0.04371569
C5ORF15	5q31.1	0.2781968	0.01138434	0.04371569
C1ORF74	1q32.2	0.27815326	0.01139773	0.04375024
TTC28	22q12.1	0.27815326	0.01139773	0.04375024
KIAA0556	16p12.1	0.2781315	0.01140443	0.04376753
CARD6	5p13.1	0.27797913	0.01145143	0.04390561
TDP2	6p22.3	0.27797913	0.01145143	0.04390561
SCAI	9q33.3	0.27791382	0.01147163	0.04394921
ZSCAN26	6p22.1	0.27776145	0.01151887	0.04411323
SCRN3	2q31.1	0.27769615	0.01153917	0.04417398
RANBP10	16q22.1	0.27756555	0.01157986	0.04430421
CYP7B1	8q12.3	0.27756555	0.01157986	0.04430421
ERAP1	5q15	0.27752201	0.01159345	0.04433918
NMNAT2	1q25.3	0.27734787	0.01164796	0.04453053
KCNA7	19q13.33	0.27732849	0.01165404	0.04453954
AZI2	3p24.1	0.27732611	0.01165479	0.04453954
KIF5A	12q13.3	0.27730855	0.0116603	0.04455205
GNG2	14q22.1	0.2771302	0.0117164	0.04474067
OPRK1	8q11.23	0.27701873	0.01175159	0.04485784
UBQLN2	Xp11.21	0.27689076	0.0117921	0.04500383
AKT3	1q43-q44	0.27686899	0.011799	0.04502155
EIF2AK2	2p22.2	0.27684723	0.01180591	0.04503065
IARS	9q22.31	0.27684723	0.01180591	0.04503065
RYBP	3p13	0.27671662	0.01184741	0.04515438
ZBTB5	9p13.2	0.27671662	0.01184741	0.04515438
ZDHHC23	3q13.31	0.27665132	0.01186822	0.04521636
HTR4	5q32	0.27661223	0.01188068	0.04525195
ACTR8	3p21.1	0.27660779	0.0118821	0.04525195
B3GALNT2	1q42.3	0.27656425	0.011896	0.04528757
PEAR1	1q23.1	0.27656425	0.011896	0.04528757
EIF5A2	3q26.2	0.27632481	0.0119727	0.04555345
FREM1	9p22.3	0.27632481	0.0119727	0.04555345
LMLN	3q29	0.27619421	0.01201472	0.04570459
SRGAP2	1q32.1	0.27617244	0.01202174	0.04572254
PTBP3	9q32	0.2759983	0.01207799	0.04588392
EFNB2	13q33.3	0.2758677	0.01212033	0.0460272
TBR1	2q24.2	0.27571379	0.01217039	0.04618209
ZNHIT6	1p22.3	0.27569356	0.01217698	0.0461983

ASPN	9q22.31	0.27554119	0.01222674	0.04635177
TBCCD1	3q27.3	0.27554119	0.01222674	0.04635177
CR1	1q32.2	0.27551942	0.01223386	0.04636113
FAT3	11q14.3	0.27541208	0.01226904	0.04646978
PNPLA8	7q31.1	0.27541059	0.01226953	0.04646978
GPC5	13q31.3	0.27538292	0.01227861	0.04649533
CFAP44	3q13.2	0.27534528	0.01229098	0.0465333
DMRT2	9p24.3	0.27528148	0.01231196	0.04660389
ASCL3	11p15.4	0.27521266	0.01233463	0.04668083
TPRXL	3p25.1	0.27516176	0.01235142	0.04673548
SLC25A36	3q23	0.27514938	0.01235551	0.04674207
LOC153684	5p12	0.27512761	0.0123627	0.0467515
ISM1	20p12.1	0.27510584	0.01236989	0.04676982
CCDC88A	2p16.1	0.27504054	0.01239148	0.04682481
KCTD10	12q24.11	0.27497524	0.01241311	0.04688875
CNOT6	5q35.3	0.27493171	0.01242755	0.04692549
TMEM127	2q11.2	0.27493171	0.01242755	0.04692549
TMEM217	6p21.2	0.27475757	0.01248545	0.04713518
GCNT7	20q13.31	0.27474472	0.01248973	0.04714241
CPXM2	10q26.13	0.2746705	0.01251449	0.0472269
ARL6IP6	2q23.3	0.27458343	0.01254358	0.04730981
TMEM273	10q11.23	0.2745305	0.0125613	0.04736765
MAP3K14-AS	17q21.31	0.27451813	0.01256544	0.04737431
POLR2B	4q12	0.27449636	0.01257274	0.04739284
PPP1R3E	14q11.2	0.27445282	0.01258734	0.0474389
EBF3	10q26.3	0.27423515	0.01266055	0.04768777
HINT3	6q22.32	0.27419162	0.01267524	0.04772504
SUN2	22q13.1	0.27419162	0.01267524	0.04772504
ADGRG1	16q21	0.27410455	0.01270466	0.04781774
EEF1AKNMT	1q24.3	0.27395218	0.01275629	0.04797579
PTP4A1	6q12	0.27389925	0.01277427	0.0480162
KLK11	19q13.41	0.27388902	0.01277774	0.04802021
C1ORF112	1q24.2	0.27386511	0.01278587	0.04803263
ZNF558	19p13.2	0.27386511	0.01278587	0.04803263
NCMAP	1p36.11	0.27384334	0.01279328	0.04805139
EPHA3	3p11.1	0.27379981	0.0128081	0.04809799
PPP2R1B	11q23.1	0.27375627	0.01282294	0.04814462
RBM25	14q24.2	0.2736692	0.01285265	0.0482471
TRAPPC11	4q35.1	0.27358213	0.01288243	0.04834977
ENKUR	10p12.1	0.27357274	0.01288565	0.04835273
NADK2	5p13.2	0.27351683	0.0129048	0.04840637
RACGAP1	12q13.12	0.27345153	0.01292721	0.04847216
ZNF841	19q13.41	0.27338623	0.01294965	0.04854716
RAB19	7q34	0.27308944	0.01305206	0.04885865
MICB	6p21.33	0.27308149	0.01305481	0.04885865
SPTLC1	9q22.31	0.27308149	0.01305481	0.04885865
MYO3A	10p12.1	0.27304415	0.01306775	0.0488887
STPG4	2p21	0.2730047	0.01308143	0.04893068

OXCT1	5p13.1	0.27299442	0.013085	0.04893484
DCLK1	13q13.3	0.27295088	0.01310011	0.04897297
ABI1	10p12.1	0.27290735	0.01311524	0.04901113
ANKS1B	12q23.1	0.27276734	0.013164	0.04915645
CD302	2q24.2	0.27275498	0.01316831	0.04916334
ARL8B	3p26.1	0.27264614	0.01320633	0.04925913
FILIP1L	3q12.1	0.2724067	0.01329032	0.04955383
PLCXD3	5p13.1	0.27229754	0.01332876	0.04967856
ZNF260	19q13.12	0.2722761	0.01333632	0.04969746
RCBTB1	13q14.2	0.27214549	0.01338247	0.04985075
NEXMIF	Xq13.3	0.27212373	0.01339017	0.04987012
TGFBR2	3p24.1	0.27210196	0.01339788	0.0498895
DOCK2	5q35.1	0.27205842	0.01341331	0.04993761
TM6SF1	15q25.2	0.27201489	0.01342875	0.04998576
